# Seasonal Intermittent Preventive Treatment for the Prevention of Anaemia and Malaria in Ghanaian Children: A Randomized, Placebo Controlled Trial

**DOI:** 10.1371/journal.pone.0004000

**Published:** 2008-12-22

**Authors:** Margaret Kweku, Dongmei Liu, Martin Adjuik, Fred Binka, Mahmood Seidu, Brian Greenwood, Daniel Chandramohan

**Affiliations:** 1 London School of Hygiene and Tropical Medicine, London, United Kingdom; 2 Ghana Health Service, University of Ghana, Legon, Accra, Ghana; 3 Navrongo Health Research Centre, University of Ghana, Legon, Accra, Ghana; 4 School of Public Health, University of Ghana, Legon, Accra, Ghana; 5 University of Ghana Medical School, Korle-bu, Accra, Ghana; Walter and Eliza Hall Institute of Medical Research, Australia

## Abstract

**Background:**

Malaria and anaemia are the leading causes of morbidity and mortality in children in sub-Saharan Africa. We have investigated the effect of intermittent preventive treatment with sulphadoxine-pyrimethamine or artesunate plus amodiaquine on anaemia and malaria in children in an area of intense, prolonged, seasonal malaria transmission in Ghana.

**Methods:**

2451 children aged 3–59 months from 30 villages were individually randomised to receive placebo or artesunate plus amodiaquine (AS+AQ) monthly or bimonthly, or sulphadoxine-pyrimethamine (SP) bimonthly over a period of six months. The primary outcome measures were episodes of anaemia (Hb<8.0 g/dl) or malaria detected through passive surveillance.

**Findings:**

Monthly artesunate plus amodiaquine reduced the incidence of malaria by 69% (95% CI: 63%, 74%) and anaemia by 45% (95% CI: 25%,60%), bimonthly sulphadoxine-pyrimethamine reduced the incidence of malaria by 24% (95% CI: 14%,33%) and anaemia by 30% (95% CI: 6%, 49%) and bimonthly artesunate plus amodiaquine reduced the incidence of malaria by 17% (95% CI: 6%, 27%) and anaemia by 32% (95% CI: 7%, 50%) compared to placebo. There were no statistically significant reductions in the episodes of all cause or malaria specific hospital admissions in any of the intervention groups compared to the placebo group. There was no significant increase in the incidence of clinical malaria in the post intervention period in children who were >1 year old when they received IPTc compared to the placebo group. However the incidence of malaria in the post intervention period was higher in children who were <1 year old when they received AS+AQ monthly compared to the placebo group.

**Interpretation:**

IPTc is safe and efficacious in reducing the burden of malaria in an area of Ghana with a prolonged, intense malaria transmission season.

**Trial Registration:**

ClinicalTrials.gov NCT00119132

## Introduction

In Africa, malaria causes over a million deaths and many millions of episodes of illness in children less than five years of age each year [1], [2]. Intermittent preventive treatment in children (IPTc), which involves administration of antimalarial drugs at pre-defined time intervals to children irrespective of whether or not they are infected with malaria, is a promising new approach to reducing the burden of malaria in children. IPTc using sulphadoxine-pyrimethamine (SP) alone or in combination with a single dose of artesunate (AS) has been shown to be efficacious in reducing the incidence of malaria in areas of Mali [3] and Senegal [4] with a short malaria transmission season. The ability of IPTc to reduce the burden of malaria in areas with a more prolonged transmission season is not known. Furthermore, resistance to SP is increasing and hence there is a need to find alternative drugs or drug combinations that could be used for IPTc. In this paper, we report the results of a randomized controlled trial of IPTc using SP or artesunate+amodiaquine (AS+AQ) in an area with an extended period of transmission in Ghana.

## Materials and Methods

The protocol for this trial and supporting CONSORT checklist are available as supporting information; see Checklist S1 and Protocol S1.

### Study area

This study was carried out in Hohoe district, Ghana. The transmission of malaria in the study area is intense with two seasonal peaks. The major wet season lasts from April to July and the minor one from September to November. The entomological inoculation rate during the study period was ∼65 infective bites per person per year (unpublished data). *Plasmodium falciparum* is the dominant malaria parasite in the area and remains sensitive to SP and AQ. A study undertaken in 480 children aged 3–59 months with symptomatic malaria at the time of the trial, described in another publication, showed that the drugs used in this trial of IPTc were efficacious. The polymerase chain reaction (PCR) corrected parasitological failure rate by day 28 was 2.3% for AS+AQ and 6.8% for SP. However, the re-infection rate by day 28 was slightly higher: 16% for AS+AQ and 19% for SP [5].

### Enrolment and randomization

From a sampling frame of all villages and communities in the district, the number of children living in each community was determined from the 2000 census. Thirty communities with the required number of children for the trial (2125) were randomly selected by probability proportional to the number of children living in each community. Several meetings were held with the health authorities and with community leaders and care givers in each selected community to explain the study objectives and procedures. Caretakers of all children aged 3 to 59 months in the selected communities were invited to a central point in the community to allow screening and enumeration of their children.

Each of the four treatment groups was sub-divided into three sub-groups to reduce the chance of accidental unblinding through recognition by trial staff of outcomes in one particular group. Thus, eligible children were randomized into 12 groups. The number of children to be allocated to each of the 12 subgroups was determined in each village/cluster by dividing the total number of eligible children living in each village (rounded to multiples of 12) by 12. For each village, the required number of tokens, marked with a study code, was prepared and mixed in a large container. Caretakers of eligible children who reported on the enrolment day picked a token from the container and their child was then allocated to the treatment group indicated by the token.

After obtaining written, informed consent, demographic information and data on socio-economic status, including the use of bednets, were collected. A clinical examination was conducted and anthropometric measurements were made. A study identity card was given to the parents of each child and they were advised to bring their child to a study health facility if the child became ill.

### Sample size

Sample size was estimated on the basis of the following assumptions: (1) the incidence of anaemia (Hb<8.0 g/dl) would be 0.28/child/year in the SP bimonthly group, 0.23/child/year in the AS+AQ bimonthly group and 0.18/child/year in the AS+AQ monthly group respectively (incidence rates for the placebo and SP groups are based on the findings of a pilot trial conducted in the study area previously); (2) loss to follow up would be 10%; (3) the study needed to have 90% power to detect a 33% difference between the SP bimonthly and AS+AQ monthly groups (likely to be the least and most effective interventions respectively) at a 5% significance level. The estimated sample size needed to meet these requirements was 2125 children (531 per arm). We also assumed that the incidence of clinical malaria would be 0.5 /child/year in the placebo group, 0.35/child/year in the SP bimonthly group, 0.25/child/year in the AS+AQ bimonthly group and 0.20/child/year in the AS+AQ monthly group. A sample size of 2125 children would have 99% power at a 5% level of significance to detect a 43% difference in the incidence of malaria between the SP bimonthly and AS+AQ monthly groups. Since more children were identified in the selected villages than needed to meet the required sample size and because the caretakers were eager to enroll their children in the study, a total of 2451 children (613 per arm) were enrolled.

### Study drugs and drug administration

Study drugs and matching placebos were supplied by Kinapharma Limited, Accra, Ghana. Their quality was confirmed at the London School of Hygiene & Tropical Medicine by dissolution and content assays for AQ and content assays for AS and SP. One of the investigator (MK) and six “drug-packers” packed the study drugs for each child in well labeled envelopes according to age and study group using the randomisation list for each village. MK and the “drug-packers” did not take any part in assessing adverse events or in measuring study outcomes.

All doses of the study drugs were administered to children by community-based volunteers. The volunteers were selected by the mothers/guardians of the children in each community to administer drugs and collect information on morbidity and adverse events and were paid an allowance of $10 a month for the periods during which they worked. Tablets were crushed and mixed with water. Children aged 3–5 months received a quarter of a tablet , those aged 6–11 months half a tablet, those aged 12–23 months three quarters of a tablet (3/4) and those aged 24 months and above received one tablet each of SP, co-formulated AS+AQ or placebo. Children received study drugs every 28 days on six occasions.

### Follow-up

Field workers visited study children to solicit any adverse events 7 to 10 days after administration of study drugs. Reported adverse events were investigated and managed by a study clinician. Field workers visited the children once a week during the period of drug administration to enquire about their health and completed a morbidity form if a child had any illness. If a child had a history of fever or vomiting within the past 48 hours the parents were advised to take their child to the nearest health facility for examination and treatment.

A passive surveillance system to monitor malaria and anaemia in study children throughout the study period was set up in the district hospital and in 21 health centres in the study area. If a child who presented at one of these facilities had fever or any features suggestive of malaria, a finger prick blood sample was collected for malaria parasite examination before treatment was given. Blood slides were read at the respective health facility to decide on treatment and read again in a central laboratory to confirm the diagnosis. Children with proven or presumptive malaria were treated with oral quinine according to the Ministry of Health (MOH) treatment guidelines.

Cross sectional surveys were carried out at the end of the intervention period (November 2005), at the end of the following dry season (April 2006) and at the end of the following rainy season (November 2006). During these surveys, temperature, weight, height and mid upper arm circumference were measured and a finger prick blood sample was collected for determination of malaria parasitaemia, haemoglobin (Hb), and markers of SP resistance. Venous blood samples were collected from a sub population of enrolled children for determination of G-6-PD deficiency and the presence of haemoglobin C and S. This was done by sampling children who were due for blood donation at the time the clinician arrived at the meeting place for the cross sectional survey. A household survey was carried out in August 2006 to determine ITN use.

Hb was measured for children attending a clinic with an axillary temperature of > = 37.5°C or a history of fever or vomiting during the past two days. Children found to have an Hb<6.0 g/dl were referred to the district hospital for management.

### Laboratory methods

Laboratory assistants, masked to study group, examined thick blood films for parasitaemia. A sample was considered negative only after 200 high power fields had been read. Parasite counts were converted to parasites per micro liter (µl), assuming a white blood cell count of 8000 leukocytes per µl of blood. If there was a discrepancy in the findings in a slide between the two initial technicians (positive or negative or a 50% or more difference in parasite density) a third, more senior microscopist read the slide and his reading was deemed to be the correct reading. A senior microscopist from the Noguchi Memorial Institute of Medical Research (NMIMR, Ghana) examined all the positive blood films and a 20% random sample of negative blood slides for quality control. Haemoglobin was measured using Hemocue ® Photometer (Leo Diagnostics, Sweden).

Phenotypic evidence for Glucose-6-Phosphate Dehydrogenase (G-6-PD) deficiency was determined by the methaemoglobin reduction method (products of SIGMA Diagnostics, St Louis USA). Sickle cell genotyping was done by haemoglobin electrophoresis.

### Data management

Data from participants were recorded on specified forms and were checked by field supervisors and data manager for consistency and accuracy. All data were entered twice into a database using EPI Data software. The accuracy of data input was checked and validated using customized validation programmes. The cleaned data were converted to Stata version 8 file by a statistician (Stata Corporation, Texas, USA) prior to analysis.

### Statistical analyses

The primary trial endpoints were the incidences of anaemia, defined as an Hb<8.0 g/dl and severe anaemia, defined as an Hb<5.0 g/dl. Malaria anaemia was defined as anaemia, defined as above, plus malaria parasitaemia. Clinical malaria was defined as a reported history of fever within 48 hours or a measured temperature of 37.5°C or greater with a positive blood slide showing asexual forms of *P. falciparum* at any level of parasitaemia. An additional case definition for malaria of fever or history of fever plus a parasite density of greater than 7000/µl was also used. Severe malaria was defined as a hospital admission which met the above definitions of malaria. Any symptom or sign that first appeared or increased in severity within seven days of drug administration was defined as an adverse event. Time at risk of primary and secondary endpoints started at the time of enrollment. Person time at risk for anaemia and malaria was calculated by subtracting the date of enrolment from the date of exit from the study. The date of exit for children who died was the date of death. The date of exit for children who were lost to follow up (migration, refusal or exclusion) was the last date of contact with the passive or active surveillance systems. Children who moved temporarily out of the study area but returned to take some of the courses of treatment remained as part of the study cohort. Four weeks per episode was subtracted from the person time at risk for children who had an episode of malaria that was treated with an effective antimalarial. Children who had an episode of anaemia were not sensored from the person time at risk because it is not clear how long a child will be risk free after an episode of anaemia.

Analysis of the primary and secondary outcomes was carried out on an intention-to-treat basis in which all randomised who received the first dose of IPTc children were included until completion or exit from the study due to withdrawal of consent, loss to follow up or death. Proportions of outcomes were analysed using chi-square test. Differences in means were calculated using t test for comparison of two means. All analyses were done with STATA software version 8.2. The incidence rate ratio (IRR) was estimated using the placebo group as the comparator. Protective efficacy was calculated as (1−IRR)×100. The Kaplan- Meier method was used to plot the time to the first episode of anaemia and malaria attack after the start of the intervention. Poisson regression was used to estimate the protective efficacy of the IPTc drugs adjusted for the effect of Hb type, G6PD deficiency and bednet use in a subset of children who had data on Hb type and G6PD status.

### Ethical approval

The study was approved by ethical committees of the Ghana Health Service/Ministry of Health (GHS/MOH) and the London School of Hygiene & Tropical Medicine. A Data Safety and Monitoring Board (DSMB) was established to monitor the trial. The DSMB approved the study protocol and its analytical plan.

### Role of the funding source

The sponsor had no role in the study design, data collection, data analysis, data interpretation or writing of the report. The corresponding author had full access to all the data in the study and had final responsibility for the decision to submit for publication.

## Results

**Figure 1:**
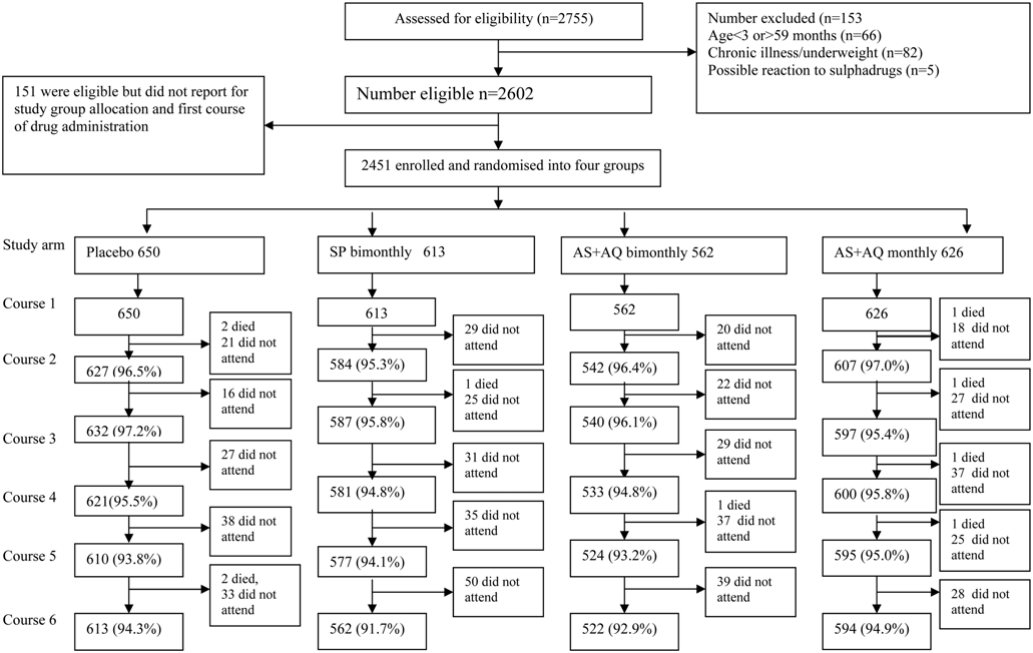
Trial Profile

Two thousand, seven hundred and fifty-five children were screened in May 2005 and 2602 (94%) were eligible for inclusion in the trial. However, only 2451 (89%) reported for enrolment and received the first IPTc dose. The number of children assigned to each group (650 to placebo, 613 to SP, 562 to AS+AQ bimonthly and 626 to AS+AQ monthly) and the follow up rates achieved in each group are shown in Figure 1. The follow up rate up to six months was high and comparable between the study groups (range 92%–95%). Mean age was slightly lower in the AS+AQ bimonthly group than in the other three groups (Table 1). There were no statistically significant differences between groups in bednet use or in anthropometric indices at enrolment. Overall, 87.7% of the children were highly adherent to treatment (received all six courses), 10.6% moderately adherent (received 3–5 courses) and 1.7% poorly adherent (received <3 courses). There was no statistically significant difference in the rate of adherence between the study groups (p>0.05).

**Table 1:** Characteristics of children in the four study groups

Characteristics	Placebo n (%)	SP bimonthly n (%)	AS+AQ bimonthly n (%)	AS+AQ monthly n (%)	p-value
Number of children enrolled	650 (26.5)	613 (25.0)	562 (23.0)	626 (25.5)	0.553
Age (in months) (mean, SD)	27.4 (14.9)	27.6 (15.8)	26.8 (15.7)†	27.0 (15.8)	0.002$
3–11	101 (15.5)	124 (20.0)	127 (22.9)	136 (21.6)†	0.007
12–23	193 (29.6)	154 (24.9)	139 (25.1)	161 (25.6)	0.197
24–35	162 (24.9)	123 (19.9)	113 (20.4)	131 (20.8)	0.113
36–47	106 (16.3)	119 (19.2)	104 (18.8)	106 (16.9)	0.453
48–59	86 (13.2)	91 (14.7)	70 (12.6)	93 (14.8)	0.617
Sex (male)	329 (50.6)	301 (49.1)	278 (50.2)	312 (49.8)	0.959
Owns bednet	186 (28.5)	178 (28.8)	169 (30.5)	193 (30.7)	0.780
Owns ITN	90 (13.8)	92 (14.9)	86 (15.5)	95 (15.1)	0.862
Slept under bednet last night	131 (20.1)	119 (19.2)	122 (21.7)	127 (20.3)	0.774
Slept under ITN last night	61 (9.4)	68 (11.0)	67 (12.1)	66 (10.5)	0.497
Stunted	0	1 (0.16)	3 (0.53)	2 (0.3)	0.281*
Wasted	80 (12.3)	61 (10.0)	71 (12.6)	85 (13.6)	0.249
Underweight	186 (28.6)	165 (26.9)	164 (29.2)	179 (28.6)	0.837
MUAC (cm) (mean, SD)	15.5 (1.634)	15.6 (1.554)	15.6 (1.521)	15.5 (1.624)	0.277$
BMI (mean, SD)	0.159 (0.024)	0.160 (0.024)	0.159 (0.026)	0.160 (0.024)	0.999$
G-6-PD Deficient	**N = 124** 18 (14.5)	**N = 139** 24 (17.3)	**N = 119** 20 (16.8)	**N = 113** 12 (10.6)	0.459
Hb genotype	**N = 110**	**N = 116**	**N = 98**	**N = 80**	
AA	89 (80.9)	70 (60.3)	58 (59.2) †	58 (72.5)	0.001
AS	15 (13.6)	25 (21.2)	16 (16.3)	10 (12.5)	0.289
AC	3 (2.7)	14 (12.1)†	20 (20.4)†	7 (8.6)	0.006*
SC	3 (2.7)	0 (0.0)	3 (3.1)	1 (1.3)	0.283*
CC	0 (0.0)	0 (0.0)	0 (0.0)	3 (3.8)	-
SS	0 (0.0)	7 (6.0)†	1 (1.0)	1 (1.3)	0.011*

† p <0.05 for the individual intervention group compared to the placebo group

* Fisher's Exact Test P values

$ ANOVA

### Anaemia and malaria during the intervention period

**Figure 2:**
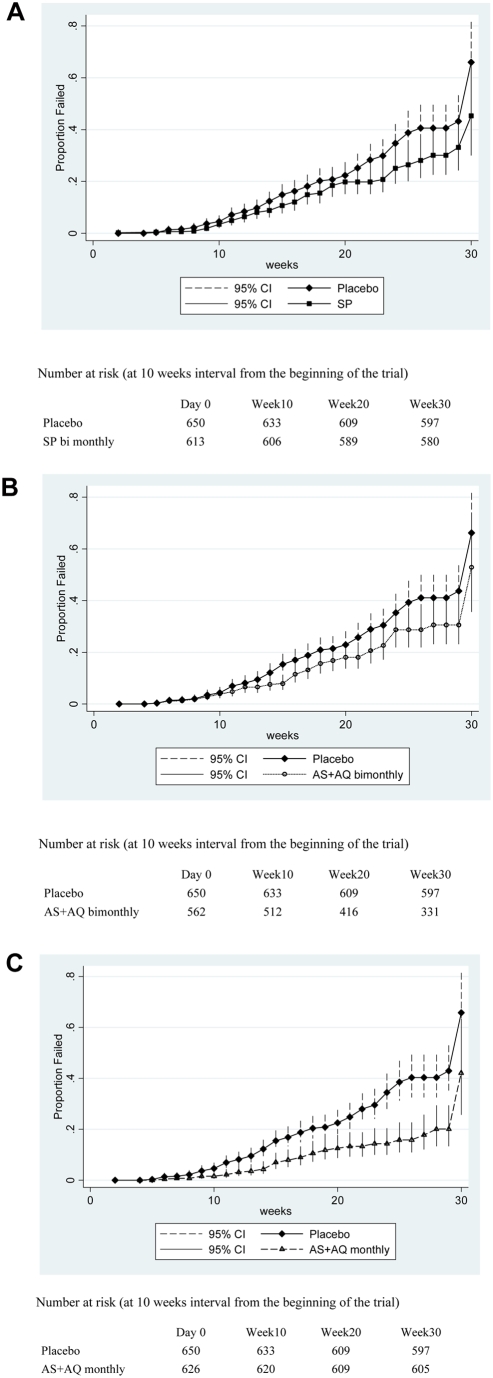
Figure 2a: Kaplan-Meier survival estimates for time to first or only episode of anaemia during the intervention period between placebo and SP groups. Figure 2b: Kaplan-Meier survival estimates for time to first or only episode of anaemia during the intervention period between placebo and AS+AQ bimonthly groups Figure 2c: Kaplan-Meier survival estimates for time to first or only episode of anaemia during the intervention period between placebo and AS+AQ monthly groups

During the six months of the intervention period, the incidence of anaemia, the primary trial endpoint, was significantly lower in all IPTc groups compared to the placebo group (Table 2). Protective efficacy against anaemia adjusted for the effect of age at enrolment and bednet use was 45% in the AS+AQ monthly group (95% CI: 25%, 60%), 31% in the SP bimonthly group (95% CI: 6%, 49%) and 32% in the AS+AQ bimonthly group (95% CI: 7%, 50%). A Kaplan-Meier survival plot (Figure 2a,b and c) shows that the time to first episode of anaemia was significantly higher in SP monthly, AS+AQ monthly and AS+AQ bimonthly groups compared to the placebo.

**Table 2:** Comparison of primary and key secondary outcomes during the six-month intervention period between study groups

Outcomes	Placebo¶	SP bimonthly¶					AS+AQ bimonthly¶				AS+AQ monthly¶			
	Episodes	Rate*	Episodes	Rate*	PE (95% CI)	p-value	Episodes	Rate*	PE (95% CI)	p-value	Episodes	Rate*	PE (95% CI)	p-value
Total OPD attendance	684	1053.8	554	974.1	1.0 (−6.3 to 7.8)	0.782	556	1003.9	3.0 (−10.5 to 4.1)	0.420	497	911.8	4.9 (−2.3 to 11.6)	0.180
Malaria with any parasitaemia	183	394.4	112	267.9	24.3 (14.1 to 33.4)	<0.001	109	282.1	17.4 (6.3 to 27.2)	0.003	44	104.0	69.1 (62.9 to 74.2)	<0.001
Malaria with high density (>7000 parasites /µl)	137	297.7	80	181.6	31.0 (19.7 to 40.8)	<0.001	77	208.9	20.4 (7.8 to 31.4)	0.002	34	73.6	70.9 (64.0 to 76.5)	<0.001
Anaemia (Hb<8.0 g/dl)	64	151.2	33	82.1	30.8 (6.4 to 48.9)	0.017	42	113.7	31.7 (7.0 to49.8)	0.016	26	63.4	45.3 (24.7 to 60.3)	<0.001
Malaria anaemia	20	10.2	15	6.1	36.2 (−16.7 to 65.2)	0.145	14	7.0	26.7 (−33.0 to 59.6)	0.307	2	1.7	82.1 (53.7 to 93.1)	<0.001
All cause admissions	25	2.4	18	2.8	−16.7 (−142 to 39.9)	0.598	9	2.7	−12.5 (−194 to 66.9)	0.980	10	2.4	0.0 (−222.0 to 53.7)	0.687
Malaria admissions	19	1.9	11	1.7	7.4 (−199 to 71.4)	0.898	7	2.1	−10.5 (−815 to 55.9)	0.368	7	1.7	15.1 (−281.8 to 81.1)	0.670

* PE: protective efficacy = (1−rate ratio)^*^100; rate ratio was **^¶^**adjusted for age at enrolment and bednet use

**Figure 3:**
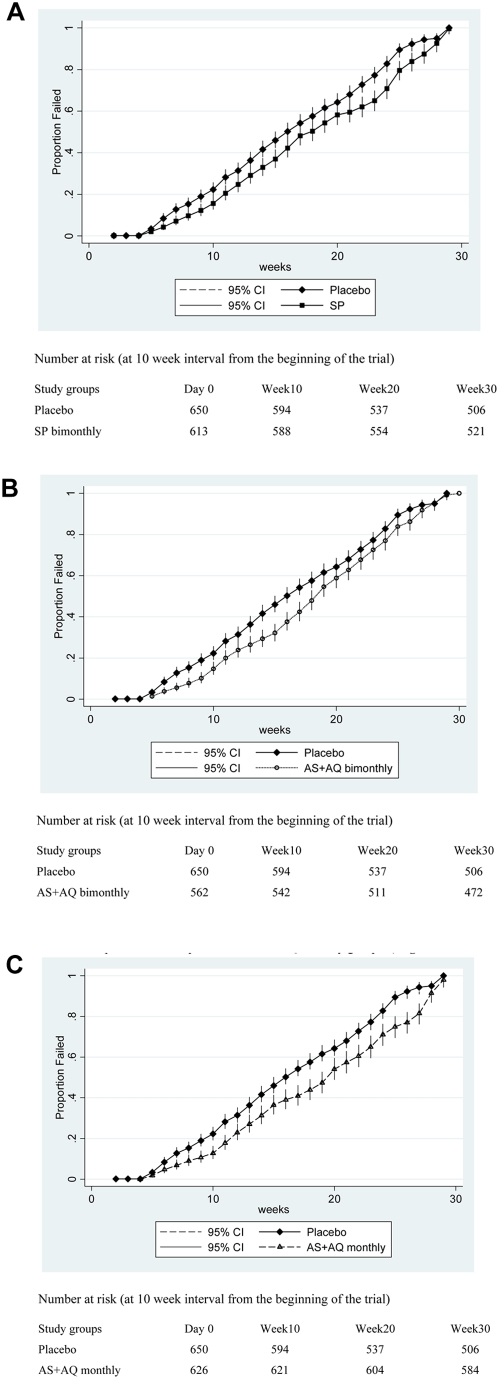
Figure 3a: Kaplan-Meier survival estimates for time to first or only episode of malaria during the intervention period between placebo and SP groups. Figure 3b: Kaplan-Meier survival estimates for time to first or only episode of malaria during the intervention period between placebo and AS+AQ bimonthly groups. Figure 3c: Kaplan-Meier survival estimates for time to first or only episode of malaria during the intervention period between placebo and AS+AQ monthly groups

The incidences of clinical malaria in children attending an OPD with any parasitaemia or high density parasitaemia during the intervention period were significantly lower in all three IPTc groups compared to the placebo group (Table 2). During the intervention period, the protective efficacy against all episodes of clinical malaria adjusted for the effect of age at enrolment and bednet use was 69% (95% CI: 63%, 74%) for the AS+AQ monthly group, 24% for the SP group (95% CI: 14%, 33%) and 17% for the AS+AQ bimonthly group (95% CI: 6%, 27%). Protective efficacy against clinical malaria with high density parasitaemia adjusted for the effect of age at enrolment and bednet use was 71% (95% CI: 64%, 76%) for the AS+AQ monthly group, 31% for the SP group (95% CI: 20%, 41%) and 20% for the AS+AQ bimonthly group (95% CI: 8%, 31%) (Table 2). The time to first or only episode of malaria unadjusted for the effect of age at enrolment was significantly higher in all three intervention groups compared to the placebo group (Figure 3a,b and c).

The differences in OPD attendance, all cause and malaria specific admissions to the hospital between the intervention groups and the placebo group were not statistically significant. In the subset of 529 children for whom there were data on Hb genotype and G6PD status, the protective effect against malaria and anaemia adjusted for the effect of age, bednet use, Hb type and G6PD status was statistically significant in the AS+AQ monthly group compared to the placebo group (Table 3).

**Table 3:** Comparison of primary and key secondary outcomes during the six-month intervention period between study groups adjusted for age, G6PD, haemeoglobin genotype and bednet use in a subset of children#

Outcomes	Placebo^$^	SP bimonthly^$^					AS+AQ bimonthly^$^				AS+AQ monthly^$^			
	Episodes	Rate*	Episodes	Rate*	PE (95% CI)	p-value	Episodes	Rate*	PE (95% CI)	p-value	Episodes	Rate*	PE (95% CI)	p-value
Total OPD attendance	64	1123.3	54	1051.4	7.5 (−44.1 to 40.70)	0.271	59	1166.1	−3.8 (−57.8 to 34.8)	0.051	49	1083.4	3.6 (−61.8 to 46.9)	0.163
Malaria with any parasitaemia	33	672.6	18	411.9	34.5 (−31.1 to 67.4)	0.232	18	398.0	29.7 (−41.8 to 65.2)	0.325	4	104.4	94.9 (61.6 to 99.3)	0.004
Malaria with high density parasitaemia (>7000 parasites /µl)	30	611.5	11	251.7	43.3 (−42.1 to 77.4)	0.226	14	309.5	20.4 (−86.4 to 66.0)	0.600	2	52.2	81.3 (17.9 to 95.7)	0.026
Anaemia (Hb<8.0 g/dl)	35	39.2	21	27.2	30.0 (−10.5 to 55.7)	0.125	20	35.4	9.7 (−58.9 to 31.7)	0.848	18	23.0	39.1 (1.3 to 62.4)	0.044
Malaria anaemia	2	40.8	2	45.8	−12.3 (−1069.0 to 81.4)	0.714	3	66.3	−43.4 (−1022.2 to 81.7)	0.731	0	0	100.0 (∼ to 100)	0.997

# Only five hundred and twenty-nine (529) children who had data on Hb genotype, G6PD status and bednet use were included in this analysis

* PE: protective efficacy = (1−relative risk)^*^100 relative risk was adjusted for Hb genotype, G6PD and net use; since there were zero observations for all cause and malaria admission data are not presented

### Anaemia and malaria during the post-intervention period

There were very few cases of anaemia or malaria in children in any of the groups during the post-intervention dry season and there were no statistically significant differences between the groups (Table 4). During the post intervention rainy season, the incidence of clinical malaria with any parasitaemia or high density parasitaemia was slightly higher in the three IPTc groups than in the placebo group but these differences were not statistically significant (Table 5). However there was a statistically significant increased risk of malaria in the post intervention period in children in the AS+AQ monthly group, the most effective protective regimen, who were under the age of one year when they received IPTc (Table 6).

**Table 4:** Comparison of primary and key secondary outcomes during the post intervention period dry season between study groups

Outcomes	Placebo¶	SP bimonthly¶					AS+AQ bimonthly¶				AS+AQ monthly¶			
	Episodes	Rate*	Episodes	Rate*	PE (95% CI)	p-value	Episodes	Rate*	PE (95% CI)	p-value	Episodes	Rate*	PE (95% CI)	p-value
Total OPD attendance	57	467.6	64	466.4	0.3 (−178.9 to 34.9)	0.422	47	362.8	22.4 (−124.2 to 50.6)	0.885	76	516.4	−10.4 (−301.7 to −4.3)	0.037
Malaria with any parasitaemia	10	3.7	14	5.0	−35.1 (−237.3 to 14.4)	0.870	15	5.8	−56.8 (−201.6 to 27.3)	0.720	7	2.5	32.4 (−125.9 to 55.8)	0.257
Malaria with high density (>7000 parasites /µl)	8	2.7	13	4.6	−70.0 (−574.9 to −4.6)	0.961	9	3.5	−29.6 (−395.5 to 15.4)	0.888	6	2.1	3.2 (−226.8 to 48.5)	0.435
Anaemia (Hb<8.0 g/dl)	9	4.6	6	3.5	23.4 (−105.6 to 75.4)	0.472	5	3.2	30.4 (−125.1 to 76.0)	0.410	3	1.6	65.2 (−263.0 to 91.0)	0.893
Malaria anaemia	7	2.4	5	1.8	25.0 (−133.1 to 77.2)	0.407	6	2.3	4.2 (−169.8 to 71.0)	0.171	3	1.1	54.4 (−606.0 to 89.5)	0.799
All cause admissions	3	2.3	3	2.5	−6.4 (−107.1 to 90.3)	0.961	6	2.7	−15.8 (−442.5 to 97.7)	0.938	5	2.4	− 3.8 (−155.9 to 93.5)	0.979
Malaria admissions	2	1.56	2	1.66	−6.4 (−159.8 to 93.4)	0.996	3	1.35	13.5 (−174.9 to 92.8)	0.918	2	0.97	37.8 (−513.3 to 97.9)	0.985

* PE: protective efficacy = (1−rate ratio)^*^100; rate ratio was **^¶^**adjusted for age at enrolment and bednet use

**Table 5:** Comparison of primary and key secondary outcomes during the post intervention period rainy season between study groups

Outcomes	Placebo¶	SP bimonthly¶					AS+AQ bimonthly¶				AS+AQ monthly¶			
	Episodes	Rate*	Episodes	Rate*	PE (95% CI)	p-value	Episodes	Rate*	PE (95% CI)	p-value	Episodes	Rate*	PE (95% CI)	p-value
Total OPD attendance	96	632.5	88	557.2	11.9 (−31.2 to −26.7)	0.895	68	473.7	6.1 (−28.6 to 31.4)	0.696	101	596.26	−6.8 (−41.7 to 19.4)	0.646
Malaria with any parasitaemia	46	15.2	59	21.1	−38.8 (−108.6 to 5.6)	0.094	41	16.0	−5.3 (−65.3 to 30.0)	0.740	60	21.0	−38.2 (−115.8 to 1.4)	0.059)
Malaria with high density (>7000 parasites /µl)	29	9.8	36	12.9	−28.7 (−112.9 to 22.2)	0.326	28	10.9	−11.2 (−88.4 to 34.4)	0.693	40	14.0	−42.9 (−145.4 to 7.0)	0.095
Anaemia (Hb<8.0 g/dl)	25	42.3	25	37.6	7.3 (−71.0 to 49.8)	0.193	13	31.0	21.7 (−61.6 to 62.0)	0.491	20	32.7	21.0 (−51.1 to 58.7)	0.523
Malaria anaemia	14	4.7	21	7.5	−51.9 (−204.0 to 24.1)	0.238	11	4.3	8.5 (−88.3 to 61.9)	0.684	16	5.6	−19.1 (−156.5 to 39.9)	0.559
All cause admissions	11	3.2	14	3.9	−21.9 (−592 to 83.6)	0.950	10	3.0	6.3 (−567.6 to 79.9)	0.871	10	2.7	15.6 (−391.1 to 78.0)	0.962
Malaria admissions	7	2.0	9	2.5	−6.2 (−463.8 to 79.9)	0.943	6	1.8	10.0 (−783.4 to 84.9)	0.888	6	1.6	20.0 (−542.9 to 83.2)	0.968

* PE: protective efficacy = (1−rate ratio)^*^100; rate ratio was **^¶^**adjusted for age at enrolment and bednet use

**Table 6:** Comparison of clinical malaria with any parasitaemia during the rainy season following the IPTc intervention between infants and older children

Age group	Treatment group	Events	PWAR^1^	Rate*	Protective Efficacy (95% CI)	p-value
15–23 months (Formerly 3–11 moths)	Placebo	6	1629	0.37		
	SP bimonthly	16	2156	0.74	−50% (−34%, 84%)	0.070
	AS+AQ bimonthly	13	2108	0.62	−40% (−68%, 81%)	0.152
	AS+AQ monthly	21	2152	0.98	−62% (−3%, 88%)	0.014
24–71 months (Formerly 12–59 months)	Placebo	40	10038	0.40		
	SP bimonthly	43	8428	0.51	−22% (−23%, 51%)	0.131
	AS+AQ bimonthly	28	7470	0.37	6% (−56%, 44%)	0.405
	AS+AQ monthly	39	9076	0.43	−7% (−48%, 42%)	0.369

PWAR^1^- person weeks at risk; ^*^Per 100 child years at risk; Protective efficacy calculated on the basis of incidence rates (1−IRR)×100.

### Cross-sectional surveys

The prevalence of malaria parasitaemia was significantly lower in the AS+AQ monthly group at the end of the intervention period but it was significantly higher at the end of the dry season following the intervention period compared to the placebo group (Table 7). There was no significant difference in the prevalences of anaemia or gametocytaemia between the study groups at the end of the intervention period or at the end of the one year post intervention period.

**Table 7:** Findings at cross-sectional surveys at one, six and twelve months following IPTc intervention (per protocol analysis)

Outcome	Placebo n (%)	SP bimonthly n (%)	p-value	AQ+AS bimonthly n (%)	p-value	AQ+AS monthly n (%)	p-value
**End of intervention period**							
Anaemia Hb<8.0 g/dl	60 (10.2)	69 (12.5)	0.196	67 (14.4)	0.143	52 (9.3)	0.491
Mean Hb (SD)	9.3 (1.33)	9.2 (1.33)	0.459†	9.2 (1.33)	0.186†	9.4 (1.22)	0.289†
Any parasitaemia	114 (19.6)	88 (16.3)	0.152	102 (20.0)	0.852	27 (4.7)	<0.001
Parasite density >7000/µl	40 (6.9)	41 (7.6)	0.642	41 (8.1)	0.458	8 (1.4)	<0.001
Gametocytaemia	6 (1.0)	1 (0.2)	0.072	2 (0.4)	0.218	4 (0.7)	0.545
**End of dry season post intervention**							
Anaemia Hb<8.0 g/dl	34 (7.1 )	33 (7.5 )	0.868	30 (7.5 )	0.905	43 (9.3)	0.243
Mean Hb (SD)	9.26 (1.04)	9.37 (1.96)	0.338†	9.18 (0.99)	0.231†	9.26 (1.15)	0.969†
Any parasitaemia	30 (6.2)	40 (0.0)	0.365	38 (9.5)	0.067	48 (10.4)	0.035
High density parasitaemia	10 (2.1)	16 (3.6 )	0.158	10 (2.5)	0.685	14 (3.0)	0.357
Gametocytaemia	5 (1.0)	3(0.7)	0.481	1 (0.2 )	0.211	5 (1.1)	0.745
**End of the rainy post intervention**							
Anaemia Hb<8.0 g/dl	47 (12.0)	42 (11.9)	0.987	47 (14.7)	0.405	36 (9.57)	0.281
Mean Hb (SD)	9.29 (1.22)	9.35 (1.33)	0.330†	9.48 (4.88)	0.327†	9.32 (3.10)	0.805†
Any parasitaemia density	155 (39.7)	138 (39.2)	0.532	129 (40.4)	0.856	158 (42.0)	0.579
High density parasitaemia	42 (10.7)	31 (8.8)	0.272	32 (10.0)	0.637	46 (12.2)	0.538
Gametocytaemia	4 (1.0)	0	0.051	4 (1.25)	0.815	4 (1.1)	0.959

† p-values from t test comparing two means

### Adverse events

Adverse events were reported slightly less frequently in each of the three IPTc groups compared to the placebo group throughout the intervention period (5.6 % vs 5.9%). Diarrhoea, vomiting, drowsiness and abdominal pains were the most frequently reported symptoms in both IPTc and placebo groups. The number of children who reported at least one adverse event or any specific adverse event did not differ significantly between the study groups. Ten deaths (6 deaths due to malaria) occurred during the intervention period and 16 deaths (11 deaths due to malaria) occurred during the 12 month post intervention period. There was no significant difference in the numbers of deaths between the IPTc and placebo groups (8 in the placebo, 8 in the AS+AQ monthly, 5 in the AS+AQ bimonthly and 5 in the SP bimonthly groups) respectively.

## Discussion

The protective effect of IPTc against malaria and anaemia observed in our study is consistent with findings from Senegal and Mali. Monthly SP+AS IPTc had a protective efficacy against clinical attacks of malaria of 86% (95% CI: 81%–90%) in Senegal [4] and bimonthly SP IPTc had a protective efficacy against clinical attacks of malaria of 40% (95% CI: 25–51%) among 6 months to 10 year old children in Mali [3]. We chose a two-monthly regimen because we were concerned about the practicality of administering SP monthly for six months in a routine national malaria control programme setting and because we have evidence from a SP IPTi trial in Ghana that SP provides about six weeks prophylaxis when used for IPT [6] We include an artemisinin in the drug combination for IPTc for two reasons. Firstly, an artemisinin will reduce the parasite load rapidly in children with parasitaemia and thus reduce the likelihood of resistance emerging to the partner drug and secondly it will clear gamtocytes and reduce the likelihood of transmission of any resistant parasites selected by the drug administration. Subsequent studies in Senegal [7] have shown that amodiaquine+SP is a more efficacious combination than AS+AQ but these data were not available when the Hohoe study was planned.

Our study has shown that even in an area with up to six months of intense transmission, monthly IPTc with AS+AQ has substantial benefits in reducing the incidence of malaria and anaemia. The combined results of studies of IPTc suggests that for drugs such as SP and AQ monthly treatment is probably necessary to achieve a high level of protection. Although IPTc was effective in reducing episodes of anaemia detected at a clinic we did not find a significant reduction in the prevalence of anaemia at the end of the IPTc intervention period. This is consistent with the observation in Senegal and with the findings of IPTi studies in Tanzania [8] and in Ghana [9]. This could be due to the fact that the study children were closely monitored and all episodes of anaemia were promptly treated with iron plus folic acid or blood transfusion in the case of severe anaemia.

We found that the prevalences of G-6-PD deficiency and haemoglobin S and C in the study area were similar to those reported from the northern and central parts of Ghana [10], [11]. IPTc was well tolerated by children with G-6-PD deficiency or haemoglobin S or C and we did not observe any episode of haemolysis following the administration of SP to children with G-6-PD deficiency. The study has shown that IPTc provides protection against clinical malaria and anaemia for both children with normal or deficient G-6-PD and those carrying haemoglobin S or C.

**Figure 4:**
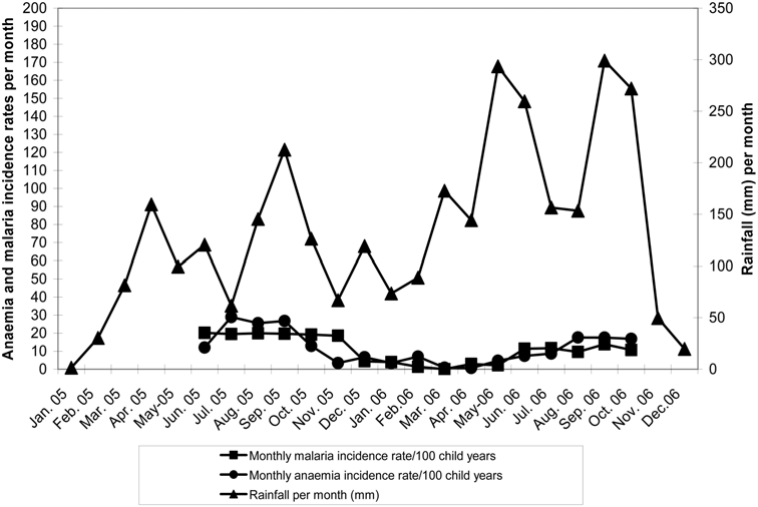
Trend of rainfall and the incidence of anaemia and malaria during the intervention and follow-up period of the study

We did not find any significant increase in the incidence of clinical episodes of malaria associated with parasitaemia at any density or at high density parasitaemia in the rainy season following IPTc intervention period in older children (12–59 months), consistent with the results of studies done in Mali and Senegal. However, we found a slight increase in the incidence of clinical malaria associated with parasitaemia of any density or high density parasitaemia in infants aged 3–11 months in the IPTc treatment groups during the rainy season following the IPTc intervention period. The overall incidence of malaria was lower during the rainy season following the intervention than during the intervention period (Figure 4). The reason for this reduction in the incidence of malaria during the post intervention period is not clear. We speculate that the high coverage of IPTc, effective treatment and an increase in the use of ITNs in the study area may have led to a reduction in the transmission of malaria. Overall, our study does not suggest that there is a significant risk of rebound of malaria if IPTc is given for just one year. It will be important to determine whether this is also the case if IPTc is given for a longer period.

SP, AS and AQ were well tolerated and no serious adverse events attributable to the study drugs were reported during the study period. The incidence of mild adverse events such as fever, general weakness, vomiting, diarrhea, abdominal pain and cough were similar in the placebo and IPTc groups. These symptoms and signs were probably due to in a large part to common childhood illnesses and not related to the administration of the IPTc drugs. Nevertheless, some mothers/guardians withdrew their children because of perceived adverse events associated with drug administration. This is a concern if IPTc is to be rolled out on a national scale. Mothers' main concerns were vomiting and drowsiness after drug administration. Clear health education messages, including information on potential adverse events and the means to overcome these events, would need to be delivered to IPTc providers and the target population in order to achieve an effective coverage of IPTc in a routine programme setting.

A recent multicentre pharmacokinetics study showed that children aged 2–5 years should be treated with 1 g sulphadoxine and 50 mg pyrimethamine to achieve drug concentrations equivalent to those needed in adults for treatment of uncomplicated malaria [12]. The doses of SP, AS, and AQ in our study were based on age not on exact weight. This could have resulted in some children receiving lower than recommended doses of SP reducing the protective efficacy of IPTc and some children receiving more than recommended dose of SP. However, in a community volunteer based delivery system an age based dose regimen is easier to implement than a weight-based dose regimen. Thus our study reflects the protective efficacy of age based dose of IPTc in a community based delivery system

Potential weaknesses of this study are that there was an imbalance in the number of children allocated to one study group for reasons that we have not been able to explain. We have explored all possible reasons as to why this might have occurred but have not been able to find any rational explanation other than chance. Since we have adjusted the protective efficacies for the effect of age at enrolment and bednet use, the potential effect modifiers, it is unlikely that this imbalance in the number of children between the groups would have biased our observation. During the intervention period, losses to follow up were very low. Because there was no active follow up during the post intervention period we cannot be sure how many children had moved out for substantial periods during this period and we had, therefore, to assume that those who took part in the last cross sectional survey had remained in the study area throughout the follow up period. Approximately 40% of children enrolled in each group did not attend the last cross sectional survey but there was no difference in the response rate between the four study groups and this loss to follow up is unlikely to have biased our observation.

We found that adherence to IPTc drugs delivered through community-based volunteers was similar to that achieved by project staff in Senegal (88% versus 91%). Delivering IPTc through community-based volunteers has advantages. They are resident in the community, making drug administration easy, they can give a repeat dose if a child vomits and can remind mothers/guardians to complete the course of treatment. However, sustaining the motivation of the volunteers for long periods is difficult. Delivering IPTc through the routine health delivery system is an attractive option because medically qualified staff are available to monitor any adverse events. However, in Ghana, only 50% of children aged between 12 and 23 months use growth monitoring services at established health posts. This means that it would be difficult to achieve a high coverage of IPTc using this route. A comparison these two modes of delivery has recently been undertaken in the Jasikan region of Ghana, an area adjacent to the one where the current study was done, and the results of this trial will be reported shortly.

## Supporting Information

Checklist S1 CONSORT Checklist
(0.05 MB DOC)

Protocol S1 Trial Protocol
(0.93 MB DOC)
