# Supplementary material for: Seasonal Intermittent Preventive Treatment for the Prevention of Anaemia and Malaria in Ghanaian Children: A Randomized, Placebo Controlled Trial
Source: PLoS One. 2008 Dec 22;3(12):e4000. doi: 10.1371/journal.pone.0004000 (PMC2602973; doi:10.1371/journal.pone.0004000)
Supplement: Protocol S1 — Trial Protocol (0.93 MB DOC) [file pone.0004000.s002.doc]

**STUDY SYNOPSIS**

**Short Title:** Impact of intermittent preventive treatment in children (IPTc) with amodiaquine (AQ) plus artesunate (AS) versus sulphadoxine-pyrimethamine (SP) alone on haemoglobin levels and malaria morbidity in the Hohoe District of Ghana

**Aim:**

- To examine how intermittent preventive treatment in children (IPTc) can improve haemoglobin (Hb) level and prevent malaria.

**Objectives:**

- To investigate whether IPTc using AQ+AS can improve Hb level and decrease malaria morbidity in young children in an area of intense and prolonged seasonal malaria transmission;
- To investigate the efficacy of IPTc using a combination of one short and one long acting drugs (AQ+AS) compared with a long acting anti-malaria drug (SP);
- To determine the optimum number of doses of IPTc for an endemic area with at least 6 months of high transmission of malaria per year;
- To investigate the potential of a rebound effect in the incidence of malaria in the year following the administration of IPTc.

**Study site:**

- Hohoe district, Volta Region, Ghana.

**Trial Design**:

- An open label randomised controlled, trial of AQ+AS, given at two different intervals (one month and two months) versus SP and placebo in children. The intervention will be carried out in the high malaria transmission season. There will also be two ancillary studies:
- An efficacy study of AQ+AS and SP which will be carried out along side the intervention at the district hospital and six health centres with laboratory facilities;
- A follow up of children who participated in the intervention to determine if any of the trial dose regimens will lead to a rebound effect in the incidence of severe malaria and anaemia.

**Sample size for intervention study:**

- 2020 children aged 3-59 months (505 per arm)

**Sample size for drug sensitivity study:**

- 436 children aged 3-59 months (218 per arm).

**Drugs for the intervention and efficacy study:**

- Artesunate in combination with amodiaquine, SP and placebo.

**Cross-sectional surveys**

Two cross-sectional surveys will be undertaken in April and November 2005. During the first survey information will be collected on the background of the child, hypersensitivity to study drugs, use of insecticide treated nets and measurement of anthropometric indices. The second survey will include the above mentioned indicators, measurement of temperature and blood samples will be collected by finger prick for determination of asexual parasitaemia, Hb and filter paper for resistance markers to SP**.**

**Follow-up:**

- From May to October 2005, participants will be visited at home 7 to10 days after every drug administration by 12 field workers. During these visits, mothers will be interviewed, and asked about any adverse event after drug administration or any episode of illness during the past one week. They will then be reminded to report at the nearest health facility if the child is unwell after the visit. The coordinators will visit participants once a week to collect information on history of fever and vomiting or any morbid episode. They coordinators will refer those who complain to them of fever or vomiting within the past 48 hours or any participants who feels unwell to the nearest health facility.
- Passive case detection will be organised in selected health centres with laboratory facilities and trained staff.
- Children who present with fever or any other symptoms potentially related to malaria at any health center within the catchment area will be examined and treated with oral quinine.
- Malaria clinical attacks will be documented and treatment with oral quinine. Treatment will be provided free of charge in all the health centres in the catchment area of the selected communities by medical assistants and qualified nurses.

**Ethical considerations:**

- Approval will be sought from the Ethics Review Board of the Ghana Health Service and from the Ethics Committee of the London School of Hygiene and Tropical Medicine.
- A written informed consent will be obtained from the parents or guardians to cover two years of intervention and follow up before any child is included in the study.

1. **BACKGROUND INFORMATION**
   1. **Burden of Malaria in Sub Saharan Africa**

Malaria is a major cause of morbidity and mortality in children in sub-Saharan Africa. In Africa, malaria causes over a million deaths and many million episodes of illness each year in children less than five years-old (Murphy et al. 2001). Despite considerable progress in malaria control over the past decade, malaria remains a serious problem, particularly in sub-Saharan Africa where about 90% **(**World Health Organisation (WHO)), 2000**)** of the global burden of morbidity and deaths from the disease occur. Infection with malaria in children is a major public health problem in tropical and sub-tropical regions throughout the world. In endemic areas, children under five years are the main group at risk for malaria.

Efforts to control progression of the disease have been hindered by the spread of resistance to chloroquine. This phenomenon, noted for the first time in Eastern Africa in 1979, is now prevalent in almost all endemic regions of the continent. Until recently, it seemed likely that sulfadoxine-pyrimethamine (SP) would replace chloroquine (CQ). However, resistance to this anti-malarial is growing quickly, even under its present controlled conditions of use. The possible loss of these two cheap and effective drugs has focused attention on the development of new tools for controlling the disease, especially in children under five years of age. Malaria preventive treatment has the potential to become in the near future a major tool for national malaria control programmes.

- 1. **Malaria as a cause of anaemia**

Anaemia is one of the leading causes of morbidity and mortality in children worldwide (WHO, 1989) and is one of the main disease burdens in developing countries.

Anaemia is said to be present when there is a decrease in haemoglobin (Hb) in the blood below the reference value for the age of the individual. Anaemia is found in all countries, rich and poor and it affects women and children particularly. Severe anaemia is often fatal, while moderate anaemia in childhood is associated with impaired physical and mental development (Geerlings et al. 2003). The prevalence and adverse effects of anaemia are most marked in the first 2 years of life (Smirth et al., 1989; Binka et al. 1994; Koram et al. 2000).

It has been observed that, while there may be several causes of anaemia, *Plasmodium falciparum* (*P. falciparum)* malaria is the primary cause of severe anaemia and mortality in children in malaria endemic areas. It has been observed that in malaria-endemic areas, the incidence of severe anemia and age-specific rates of anemia strongly correlate with the intensity of *P. falciparum* transmission (Koram et al. 2000), and that significant improvements in haematological indices have been seen after malaria control (Kurtzhals et al. 1997; Menendez et al. 1997).

Asymptomatic malaria also causes significant reduction in Hb level in children. This may be the result of direct destruction of parasitized red blood cells through immune mechanisms that destroy both parasitized and non-parasitized red cells or due to suppression of the bone marrow as a result of the infection (Kurtzhals et al. 1997). Kurtzhals and colleagues also observed that early treatment and elimination of malaria parasites results in the recovery of Hb levels in children with acute malaria and these mechanisms may operate in asymptomatic infections in Ghanaian school children (Kurtzhals et al. 1997). It has been reported that around 75% of preschool children in East Africa are anaemic (Snow et al. 1997) and anaemia in childhood is associated with impaired physical and mental development (Lozoff et al. 1991).

Several studies have shown that iron supplementation increases the haemoglobin level in young children resident in malaria endemic areas (Smith et al.1989; Menendez et al. 1997) However, concerns have been expressed about the effect of iron supplementation on malaria (Oppenheimer et al.1989). An increased incidence of malaria as a result of iron supplementation can occur under some circumstances, but there is a general consensus that the effect is not marked and is out-balanced by the beneficial effect of iron on anaemia. Daily iron supplementation given by mothers reduced the incidence of severe anaemia by 32% in infants in a highly malaria endemic area of Tanzania without increasing clinical episodes of malaria in children who received iron (Menendez et al. 1997). In this study, additional administration of malaria chemoprophylaxis (weekly Deltaprim (pyrimethamine/dapsone)) led to a 57% reduction in severe anaemia and to a 60% reduction in episodes of malaria. These results support iron supplementation for infants, even in malaria endemic areas, and suggest an important role for chemoprophylaxis.

Chronic parasitaemia in infants is associated with anaemia (Kitua et al. 1997). A study in Tanzania showed that at any point in time, the mean packed cell volume (PCV) in infants with low parasitaemia (<1000 parasite/µl) was higher than that of infants with intermediate (1000-9999 parasite/µl) or high parasite densities (≥10000 parasite/µl). This suggests that interventions which lower parasite densities in infants may reduce severe anaemia and its associated morbidity and mortality. On the other hand, it has been suggested that the life-time risk of severe malaria is influenced by an ability to develop immunity early in life as a consequence of repeated infections during a period when other protective mechanisms, such as maternal antibodies and breast feeding, are operating (Snow et al. 1997). Thus, it is necessary to achieve a balance in which chemoprophylaxis or intermittent treatment reduces substantially the risk of severe disease during first year of life without markedly affecting the development of immunity.

2.0 PREVENTION OF MALARIA AND ANAEMIA IN CHILDREN

Strategies for preventing malaria in children, the most vulnerable group, include provision of prompt access to diagnosis and effective treatment of clinical malaria. Other options are the use of effective anti-malarial drugs as chemoprophylaxis to treat or suppress parasitaemia, or as intermittent preventive treatment, and reducing contact with the vector by the use of insecticide treated bednets or insect repellents.

**2.1 Use of insecticide treated materials (ITMs)**

Community-based strategies to prevent malaria infection and reduce the intensity of exposure in young children are needed in sub-Saharan Africa. Insecticide treated materials (ITMs) such as bednets and curtains have been shown to reduce all cause mortality from 15% to 25% (Alonso et al, 1991; D’Alessandro et al. 1995; Nevill et al, 1996; Binka et al, 1996, Habluetzel et al. 1997)), they can also reduce malaria specific mortality (D’Alessandro et al. 1995; Binka et al, 1996), uncomplicated malaria episodes (Sexton et al. 1990; Beach et al. 1993), rates of high density parasitaemia (Habluetzel et al. 1999), and the prevalence of malaria parasitaemia (Moyou-Somo et al. 1995) and anaemia (Marbiah et al. 1998).

Holtz and his colleagues in Malawi recently reported a higher mean Hb level (10.6g/dl (95% CI; 10.2, 11.0) in children under five years of age in households with one or more insecticide treated nets (ITNs), than in children in households with no ITNs (Hb=9.8g/dl 95% CI; 9.5, 10.1). Anaemia (Hb<11.0 g/dl) was more prevalent in children in households without ITNs compared with those with ITNs, 72.4% vs 63.3% respectively (Holtz et al. 2002).

The use of ITNs has shown some benefit on maternal health in low transmission areas (Dolan et al. 1993; D’Alessandro et al. 1996), but ITN failed to show any appreciable benefit in two high transmission areas (Shulman et al. 1998; Browne at al. 2001). However, the study in The Gambia showed that pregnant women did obtain some protection from ITNs. These findings have subsequently been confirmed in a large study in western Kenya with intense perennial transmission which showed that women who were protected by ITNs during their first four pregnancies had a significant reduction in parasitaemia by 38% (95%CI, 17-54) and a 28% (95%CI, 2-47) reduction in the incidence of low birth weight babies (Kuile et al. 2003*).*

The WHO (2000) Cabinet Project, Roll Back Malaria (RBM), includes wide spread use of ITMs in its strategic plan to eliminate 50% of malaria-related mortality over the next 10 years. In the Abuja Declaration on RBM in Africa on 25^th^ April 2000, African heads of state and government delegations resolved that by the year 2005, “at least 60% of those at risk of malaria, particularly pregnant women and children under five, benefit from the most suitable combination of personal and community protective measures such as ITNs and other materials to prevent infection and suffering”.

Since ITNs reduce malaria morbidity and mortality in children, it has been suggested that childhood disease episodes may be triggered by new infections rather than pre-existing infections. The pathology of malaria during pregnancy on the other hand, could result from pre-existing parasitaemia (Mutabingwa et al. 2001). ITNs have been found to be protective and cost effective. A study in West Kenya analysed the cost and sustainability of ITNs delivery through employer-based malaria control strategy. It was found that, the cost of distributing ITNs to workers which was estimated to be US$15.8 could be reduced to US$0.5 if the remaining stocks were liquidated and by another US$1.3 if the salvage of capital items is considered (Njugi et al. 2004).

Alternatively, a single infection alone could adversely affect pregnancy, as ITNs may prevent some, but not all infective bites (Nosten et al.1999). A study in northern Ghana showed that transmission peaked between the hours of 0400hrs to 0600hrs, a time when most adults, including pregnant women may be up and working (Appawu et al. 2004). It is thus probable that effective parasite clearance with an antimalarial in addition to the use of insecticide treated bed nets would have an additional impact on malaria in pregnancy.

Studies on the use of ITNs on childhood morbidity and mortality have presented varied results from different parts of sub-Saharan Africa, although all have shown some protective efficacy against mortality in children less than five years of age. The differences are shown in table 1. These might be related to the proportion of people using the nets correctly or differences in epidemiology of malaria such as the number of infective bites per person per year. In some areas there has been a long tradition of use of bednets. Also sleeping habits of the people, such as sleeping outside the room when the nights are warm are likely to vary in different areas. ITNs therefore, need to be used together with other preventive strategies in order to get maximum protective effect against malaria morbidity and mortality in children.

**Table 1:** Summary results from four TDR supported trials in Africa to measure the impact of insecticide-treated nets on overall child mortality in children aged 1-59 months.

EIR= Entomological Inoculation Rate. Protective efficacy calculated as 1-relative rate (incidence in the intervention group/incidence in the control group).

| Country (reference) | Intervention | Transmission intensity  EIR | Intervention group death rate (/1000/year | Control group death rate (/1000/year | Unadjusted protective efficacy | Rate  Difference  (deaths  averted  /1000  /year) |
| --- | --- | --- | --- | --- | --- | --- |
| Gambia  D’Alessandro et al. 1995 | Treatment of existing nets | 1-30 | 18.7 | 24.3 | 23%  (1-41%) | 5.6  (2.0-9.2) |
| Kenya  (Nevil et al. 1996 | Bednets plus insecticide | 10.30 | 9.4 | 13.2 | 29%  (3-47%) | 3.8  (1.1-6.6) |
| Ghana  (Binka et al. 1996) | Bednets plus insecticide | 100-300 | 28.2 | 34.2 | 18%  (1-30%) | 6.0  (2.3-9.5) |
| Burkina Faso(Habluetzel et al. 1997) | Curtains plus insecticide | 300-500 | 41.8 | 48.7 | 14%  (-8-30%) | 6.9  (2.0-11.8 |

- 1. **Anti-malarial drugs and reduction of malaria transmission**

Anti-malarial drugs have been used in various ways to prevent malaria in the residents of endemic areas for many decades. The primary objective of most early studies of widespread distribution of anti-malarial drugs to the population of malaria endemic areas was interruption of malaria transmission. Although this was not achieved, administration of anti-malarial drugs led to a marked reduction in the prevalence of malaria infection and in the incidence of clinical attacks. Table 2 shows the various approaches in which anti-malarial drugs have been used in the past and currently being used.

**Table 2:** Approaches to the administration of anti-malarial drugs as a means of preventing malaria in the population of malaria endemic communities.

| Approach | Comments |
| --- | --- |
| Treatment of clinical cases | Requires that a high proportion of infections are symptomatic and that patients have easy access to treatment.  Likely to be more effective if treatment include a gametocidal drug. |
| Medication of salt | Produces sub-therapeutic but cumulative blood levels.  Highly likely to induce resistance |
| Mass drug treatment | Involves administration of drugs usually in therapeutic doses, to infected and non-infected subjects.  Usually has only a transitory effect on levels of parasitaemia and clinical malaria |
| Chemoprophylaxis | Involves the repeated administration of drug, usually at sub-therapeutic doses, over a sustained period so as to obtain persistent protective blood levels |
| Intermittent preventive treatment | Involves the administration of a therapeutic dose of drug over an intermittent and defined period |

Greenwood B. 2004.The use of anti-malaria drugs to prevent malaria in the population of malaria endemic areas *Am J Trop Med Hyg* Vol. 70, No 1.

- 1. **Treatment of symptomatic cases**

The strategy for preventing malaria through prompt access to diagnosis and effective treatment of clinical malaria started in the early part of the 20^th^ century (Harrison et al. 1978). This was the time when quinine was extensively used in Italy and other places. The idea was that treatment of symptomatic cases with an effective anti-malarial would reduce transmission and thus provide indirect protection to the population as a whole.

Quinine which was the drug used has, however, very little or no effect on gametocytes. Thus, it is unlikely that treatment of cases with quinine had much impact on the overall transmission of the infection. Recent studies in South East Asia and South Africa have shown that treatment of clinical cases with drugs, such as an artemisinins, which kill gametocytes as well as asexual parasites, is more likely to be effective.

**2.4 Mass drug administration (MDA)**

Mass drug administration (MDA) involves administration of drugs in therapeutic doses, to infected and non-infected subjects. In the highly endemic areas in Africa, in contrast to the situation in southeast Asia and South Africa, most malaria infections are asymptomatic and go untreated, even when quite high levels of parasitaemia are present (Von Seidlein et al. 2002). In such situations, a significant reduction in the transmission of malaria by administration of a gametocidal drug is likely to be achieved only if asymptomatic subjects are treated. Detection of asymptomatic subjects requires a major investment in surveillance so the concept of MDA was developed. During an MDA, the whole population of a community known to contain a number of asymptomatic subjects is treated without determining who is infected. Anti-malarials were distributed widely in highly endemic areas of Italy during the 1920s and the 1930s, partly as personal prophylaxis but also to reduce transmission. Quinine was use extensively by Gorgas during the construction of the Panaman Canal, in part as personal prophylaxis for canal workers, but also to reduce transmission. The use of MDAs to reduce transmission became a more promising approach following the discovery of 8-aminoquinolines, which are gametocidal. One of the first studies to investigate the potential role of this drug in interrupting transmission was carried out in a rubber plantation in Liberia in 1930 where mass treatment of the population of two camps with Plasmoquine led to a marked decrease in parasite prevalence and reduction in the number of infected mosquitoes in the treated camps (Barber et al. 1932).

During the 1960s and 1970s, a number of trials of MDA were under taken in Africa and Asia with the primary aim of interrupting transmission. In nearly all cases transmission was not interrupted although there was a marked reduction in prevalence. The most ambitious attempt to interrupt malaria transmission in a high transmission area of Africa using the MDA approach was undertaken in Garki in northern Nigeria in the 1970s (Molineaux et al. 1980). Household spraying with residual insecticide was combined with several rounds of MDA with sulfalene-pyrimethamine. Parasites and spleen rates decreased substantially in the intervention communities, but transmission was not interrupted. However, episodes of clinical malaria, and probably deaths, were reduced during the period of the intervention. The largest MDA reported was conducted in Nicaragua in 1980. A single round of treatment with CQ plus Primaquine was given to approximately eight million people (Garfield et al. 1983). There was a massive decrease in the incidence of malaria immediately after the drug administration, but transmission was not interrupted and the incidence of malaria soon returned to its previous levels. A recent study of MDA was undertaken in the Gambia with the aim of delaying the onset of transmission in the area where malaria is highly seasonal and transmission limited to a few months of the year (Von Seidlein et al. 2003). Sulphadoxine-pyrimethamine and AS were given to the whole of the population of 33 villages at the end of the dry season when gametocyte levels are at their lowest. A reduction in the incidence of malaria compared with that seen in nine control villages where placebo was distributed was observed during the month after the administration of the drug combination, but no overall effect on the incidence of malaria throughout the course of the malaria transmission season was seen.

One success for MDA in achieving the goal of interruption of transmission has been reported recently from the island of Aneityum, Vanuatu. Eight rounds of MDA with chloroquine (CQ)-SP-primaquine combined with the introduction of ITNs and environmental control measures led to the elimination of falciparum infection from the island (Kaneko et al. 2000).

In most MDAs, medication has been given in the form of tablets, sometimes under supervision. This ensures that an effective dose is given, but if large populations are to be covered on a repeated basis, this approach to drug delivery is demanding.

During the 1950s and 1960s a number of trials of medicated salt were undertaken in malaria endemic areas (Payne et al. 1988). These generally achieved their objective of reducing the incidence of clinical episode of malaria, but only at a cost of rapid emergence of resistance (Meuwissen et al. 1964; Giglioli et al. 1967). Studies in experimental systems have shown that exposure of malaria parasites to a sub-therapeutic dose of an anti-malarial over a prolonged period of time is the optimum way of inducing resistance. Use of medicated salt is likely to have a similar effect and has no place in malaria control.

**2.5 Chemoprophylaxis**

**2.5.1 Prevention of malaria by chemoprophylaxis in children**

Chemoprophylaxis is highly effective in reducing mortality and morbidity from malaria in young children and pregnant women living in endemic areas, but is difficult to sustain and, in some studies, has impaired the development of naturally acquired immunity. Studies have shown that children who received chemoprophylaxis had fewer episodes of malaria, grew better, had a higher mean Hb and a lower gamma globulin concentration than those in the control group (Mcgregor et al.1956). Other studies conducted subsequently in other countries in Africa have supported these results (Prinsen et al. 2003).

A large trial of chemoprophylaxis in children less than five years of age was conducted in The Gambia in the early 1980s (Greenwood et al. 1988) using pyrimethamine plus dapsone given by village health workers throughout the rainy season for a period of five years. The result showed that overall mortality in children who received prophylaxis was reduced by approximately 35%. Protected children had fewer clinical attacks of malaria and a high mean packed cell volume than control children. These results were sustained during several years of observation (Allen et al. 1990) and are at least as impressive as those obtained form ITNs. A recent study in Tanzania using the same anti-malarial also showed a marked reduction in clinical attacks of malaria and the incidence of severe anaemia (Menendez et al. 1997).

Chemoprophylaxis is now largely discouraged due to the threat of resistance and logistic difficulties in sustaining it as a control strategy on a long-term basis. In children, this strategy has been found to be associated with impairment of the development of natural immunity as children experience an increase in clinical attacks of malaria during the year after chemoprophylaxis was stopped (Greenwood et al. 1995). New strategies are being sought, which mitigate some of the disadvantages of sustained chemoprophylaxis but retain some of its benefits. Among these is drug administration given on an intermittent basis during periods of high transmission.

- - 1. **Chemoprophylaxis in pregnant women**

Until recently, WHO recommended that pregnant women resident in areas of moderate or high malaria transmission should receive chemoprophylaxis throughout the second and third trimesters of pregnancy (WHO, 1986). Traditionally, this has been with CQ but the potential usefulness of this intervention has been nearly eliminated by the emergence and spread of CQ resistance in much of the world and poor compliance with the weekly regimen, necessitating the need for other strategies (Steketee, 1989; Heymann et al. 1990; Bloland et al. 1993; White, 1999). Other drugs that have been used for chemoprophylaxis include pyrimethamine, which was used effectively on a large scale in Nigeria (Morley et al. 1964; Fleming et al. 1986). However another study conducted in Nigeria in which 67% (59/88) of *in vivo* and 60% (6/10) of *in vitro* tests showed pyrimethamine resistance, rendering this approach ineffective (Nahlen et al.1989). Pyrimethamine-dapsone given fortnightly was associated with higher mean birth weights and packed cell volumes in The Gambia (Greenwood et al.1989). Daily administration of proguanil to primigravidae significantly reduced parasitaemia and severe anaemia in Nigeria (Fleming et al. 1986). It was also associated with a reduction of parasitaemia and clinical episodes of malaria when combined with CQ in Tanzania (Mutabingwa et al. 1993). In Malawi and southeast Asia, weekly prophylaxis with mefloquine has been used to prevent malaria in pregnancy (Nosten et al. 1994; Steketee et al. 1996).

Substantial efforts are made to ensure drug safety, availability and a high level of compliance during studies. However, when drugs are introduced into routine practice, these optimal conditions are never met, resulting in low compliance. The poor compliance associated with this strategy and the emergence of drug resistance has contributed to the ineffectiveness of malaria chemoprophylaxis programmes in Africa (Laing, 1984).

- 1. **Intermittent preventive treatment in pregnancy (IPTp)**

Intermittent preventive treatment (IPT) is the administration of a full therapeutic course of anti-malarial drugs to at risk subjects such as pregnant women and children at specified times regardless of whether they are infected. This approach which does not involve continuous drug administration, is less likely to contribute to the emergence of drug resistance than chemoprophylaxis.

Intermittent preventive treatment in pregnancy (IPT_p_) involves the administration of full curative doses of an effective anti-malarial drug at specified intervals during pregnancy. Its operative advantage is that it can be provided as part of routine antenatal care, as it is estimated that an average of more than 70% of African women attend ANC at least once during pregnancy. A study in Malawi, where chemoprophylaxis with weekly CQ was compared with a full course of SP given twice showed a lower prevalence of low birth weight (Shultz et al. 1994). This has been confirmed in larger effectiveness studies even when only one dose was given (Rogerson et al. 2000). In Kilifi, Kenya, Shulman and others (1999) have also demonstrated that women who received a single dose of IPTp with SP benefited significantly; the protective efficacy of a single dose in reducing severe anaemia was 50% (95% CI, 6-73, p-value 0.027).

The emergence and spread of SP resistance will increasingly undermine the use of SP for IPTp strategy, depleting the currently available and affordable drugs that can be used for interventions during pregnancy (White, 1999). It is, therefore, important that alternative anti-malarial drugs that are safe and effective in pregnancy are identified.

**2.6.1 Intermittent preventive treatment in infants (IPTi)**

Several studies carried out in tropical Africa have shown that regular administration of anti-malarial drugs throughout early childhood increases haemoglobin levels substantially (Geerlings et al. 2003; Bradley-Moore et al. 1985). However, when chemoprophylaxis is stopped there may be a rebound in the incidence of clinical malaria (Greenwood et al.1995) or severe anaemia during the following months (Menendez et al.1997). Intermittent preventive treatment in infants (IPTi) in which antimalaria are given intermittently throughout early life provides a possible way of providing some protection against the serious clinical consequences of malaria infection without impairing the development of immunity to the infection.

In Tanzania, Schellenberg and colleagues showed that IPTi with SP given at 2, 3 and 9 months of age together with daily iron supplementation from 2-6 months of age, reduced the incidence of severe anaemia (PCV <25%) by 50% and episodes of malaria by 59% without significant rebound infection in to severe malaria or anaemia during the second year of life (Schellenberg et al. 2000). Another study in northern Tanzania, showed that IPTi with amodiaquine (AQ) given at 3, 5 and 7 months of age plus daily iron supplementation reduced malaria fever and anaemia by 65% and 67% respectively without any significant rebound effect after discontinuing (Massaga et al. 2002). Although these results suggest that IPTi with SP or AQ is an efficacious and safe intervention for reducing the burden of malaria and anaemia in infants, these results can not be generalised all the to various ecological and transmission settings in Africa. In the study in Ifakara, Tanzania the prevalence of parasitaemia in 12 month-old infants (4%), the incidence of clinical malaria (36/100/year) and proportion of febrile episodes attributable to malaria (14.5%) were low in the placebo group compared to the expected values in high transmission settings in Africa. The low burden of malaria in the study area in Ifakara, Tanzania is likely to be due to the fact that there was a high use of bednets in the study population (67%) and that the study infants lived within 5km from a relatively well equipped mission hospital. In contrast, the prevalence of parasitaemia in <12 month old infants in Navrongo, Ghana during the high transmission season was 60% (Owusu et al. 2002). The proportion of febrile episodes attributable to malaria ranges from 7% to 60% in sub-Saharan Africa (Chandramohan et al. 2002). Thus, further evidence is needed before recommending the use of IPTi as a means of reducing burden of malaria in high transmission areas in rural Africa. So far, few community trials have evaluated the effect of IPTi in preventing malaria (Kanyok, 2001).

**2.6.2 IPTi consortium**

With the need for a comprehensive assessment of this strategy, WHO, UNICEF and several leading centres of malaria research, including the LSHTM and the CDC have formed a consortium, which has developed a research programme that will answer the important scientific questions about this promising and innovative form of malaria control, and move the intervention into policy and practice. By the end of 2008, the consortium will have generated additional information on issues surrounding the choice of IPTi, the relationship between IPTi and the development of drug resistance, the cost-effectiveness, acceptability, mortality impact, community effectiveness and impact on the immunological response to *P. falciparum* infection. Studies to generate the information are being carried out across several types of settings, including Ghana, Tanzania, Kenya, Gabon and Mozambique where malaria transmission is intense and the burden of the disease is not only among infants.

**2.6.3 Intermittent preventive treatment in children (IPTc)**

Intermittent preventive treatment in children (IPTc) is a promising new approach to malaria control, which targets children less than five years of age. In high transmission areas, where a major proportion of deaths and severe morbidity from malaria occurs during the first year of life IPTi could make a major contribution to reducing the burden of malaria as a whole. However the IPTi strategy cannot be the solution for large-scale malaria control plans in parts of Africa where the burden of the disease is not predominantly in infants as is the case in Ghana. In these settings malaria transmission is throughout the year but peaks during the rainy season between May and October. During this period, mortality and morbidity from malaria in children is very high. IPTi even if highly effective would have only a limited impact on the overall burden of malaria in children. IPTc, which targets children aged less than five years old, should be considered for such areas.

So far, few community trials have evaluated the effect of IPTc in the prevention of malaria.

Dicko and his colleagues in Mali, an area of seasonal malaria transmission gave two doses of SP to children aged 6 months to 9 years at an interval of two months. They found a protective efficacy of 40% among children in the intervention group compared to the placebo group (Dicko et al. 2004).

A similar study was carried out in 2002 in the Hohoe district (Kweku et al. unpublished). In this study, monthly SP was given to children aged between 9 and 23 months every month for five months in the high transmission season. The results showed an improvement of mean Hb by 0.5g/dl (9.8g/dl to 10.3g/dl) (SD, 1.23) at the peak of the high transmission season in the intervention group. This intervention also resulted in a protective efficacy against malaria parasitaemia of 39% and against anaemia (Hb<11.0g/dl) of 26% at the end of the high transmission season.

Recent work in Niakhar, Senegal has assessed the use of IPTc in an area of intense but strongly seasonal malaria transmission and has given promising results. The study used a combination of a long and short acting drug. Intermittent AS+SP was given every month for three months during the rainy season which lasted only for four months.

The study showed that IPTc using AS+SP was well tolerated without any drug-attributable adverse events. There was a dramatic reduction in the parasite density even when a clinical episode occurred in children under five years. More importantly, the rate of clinical malaria was markedly lower in the intervention compared to the placebo group, giving a protective efficacy of 86% and there was no rebound effect in the year following the intervention (Cisse et al. 2004).

Although these results suggest that IPTc with AS+ SP is an efficacious and safe intervention for reducing the burden of malaria and anaemia in children, there is very little information on the use of combination therapy in IPTc. There are important questions to be answered about IPTc before any implementation on a large scale takes place as shown in table 3 below.

**Table 3:** Outstanding issues on the implementation and drug to be used for IPTc in an area of intense and prolonged high transmission season.

| 1 | What drug could be used to replace SP for IPTc in areas of high SP resistance |
| --- | --- |
| 2 | Is IPTc using drug combination efficacious in an area of perennial transmission with a prolonged high transmission season? |
| 3 | What will be the efficacy of IPTc using a long acting (SP) plus a short acting (AS) anti-malaria drug compared with a combination of along acting (AQ) plus a short (AS) acting anti-malaria drugs? |
| 4 | What is the optimum number of times to give a combination of the long plus short acting anti-malaria drugs in an area with prolonged high transmission season? |
| 5 | What will be the extent of a rebound effect in the incidence of malaria in the following year using AQ in place of SP. |

**2.7 Pharmacological characteristics of drugs that have been used for IPTi**

**2.7.1 Sulphadoxine-pyrimethamine (SP)**

Sulphadoxine-pyrimethamine and AQ are the only two drugs studied so far for IPT. Pyrimethamine is a dihydrofolate reductase inhibitor. It is a tissue schizontocide and a slow-acting blood schizontocide with some sporontocidal activity. It is almost completely absorbed from the gastrointestinal tract and metabolized in the liver. Its combination with sulphadoxine, a long-acting sulphonamide produces enhanced antimalarial activity. Sulphadoxine and pyrimethamine are highly protein bound with relatively long mean elimination half-lives of around 180 hours and 95 hours respectively. Pyrimethamine is extensively metabolized whereas only a small proportion of sulphadoxine is metabolized to acetyl and glucuronide derivatives. Excretion is mainly in the urine, although both compounds cross the placental barrier and are also detected in breast milk.

The combination is generally well tolerated when used at recommended doses for malaria therapy. The most serious events are associated with hypersensitivity to the sulpha component, involving the skin and mucous membranes, and normally occur after repeated administration. Serious cutaneous reactions following single dose treatment with SP are rare. The combination is no longer recommended for prophylaxis, this follows reports of erythema multiforme and toxic epidermal necrolysis in 1:5000 to 1:8000 Caucasian subjects who took the drug for weekly prophylaxis. Cutaneous drug reactions are more common in HIV positive patients. Haematological changes including thrombocytopenia, megaloblastic anaemia and leucopenia, have also been observed; these are usually asymptomatic and regress after withdrawal of the drug.

The mechanism of the protective effect of SP or AQ when used for IPTi is not clear and this needs to be explored. If it is mainly mediated by treatment, then all effective short-acting anti-malarial drugs could be used for IPTi, and the range of appropriate drugs is the same as that for treatment of malaria, with the expectation that local patters of resistance can accurately predict which drugs will work for ITPi. If, on the other hand, the predominant effect is mediated through prophylaxis then only long-acting drugs need be considered. This means that IPTi with these drugs given three times in a year would provide chemoprophylactic protection for three months in addition to clearing parasites present at the time of their administration. If the protective effect of IPTi on the incidence of severe anaemia is primarily due to its chemoprophylactic effect, then IPT with an anti-malarial drug with a short plasma half life may not have a significant effect on the incidence of anaemia.

The effectiveness of short and long acting anti-malarial drugs for use in IPTi may vary according to transmission intensity. In highly endemic areas, where infants receive at least one infective mosquito bite every week (EIR >50/year), the chemoprophylactic effect of IPTi may be essential as it reduces the number of infections during infancy and thus reduces the risk of severe anaemia. However, in low endemic settings where the number of infective bites is less than one per month, the chemoprophylactic effect of IPTi may not be essential and IPTi with short acting antimalarial drugs, which results in clearance of all parasites present at the time that the drug is given, may be as effective as IPT with long acting ones in reducing the incidence of anaemia. Studies of IPT in areas with different, and well defined, levels of transmission are needed to clarify this point and are on-going.

**Table 4: Plasma half life and duration of chemoprophylactic effect of antimalarial drugs that might be used for IPT**

| Drug | Plasma half life  Mean (range) | Duration of chemoprophylaxis |
| --- | --- | --- |
| Sulfadoxine-Pyrimethamine | 7 days (4-10 days)  4 days (2-6 days) | 4 weeks |
| Amodiaquine | 4 days | 3 weeks |
| Mefloquine | 14 days | 4 weeks |
| Chlorproguanil/ Dapsone | 12 hrs (6-18 hrs)  24 hrs (18-30 hrs) | <1 week |
| Artesunate | 6 hrs (4-11 hrs | < 1 week |

Source of plasma half life data: Compodium of data sheets and summaries of product characteristics 1999-2000. Datapharm publications Ltd 1999.

**2.7.2 Amodiaquine**

Amodiaquine is the only drug that has been used so far for IPTi apart from SP. Amodiaquine resistance is variable across Africa and does not appear to have progressed as fast as resistance to SP (Olliaro et al. 1996). Amodiaquine, a 4-aminoquinolone has been used widely in the past to treat and prevent malaria. Though the potential cross resistance between the 4-aminoquinolones has led to arguments against replacing CQ with AQ, it is still effective in some areas where CQ resistance is prevalent (Watkins et al. 1984; Muller et al. 1996; Bloland et al. 1996; White, 1996; Brasseur et al. 1999; Sowunmi et al. 2001; Ndounga et al. 2003). Treatment with AQ is safer than AQ prophylaxis (Winstanley et al. 2002). Amodiaquine hydrochloride is readily absorbed from the gastrointestinal tract. It is rapidly converted in the liver to the active metabolite desethylamodiaquine, the parent compound being detectable for no longer than 8hours. The terminal elimination half-life of desethylamodiaquine is up to 18days as shown in table 4. Adverse reactions to standard doses of AQ used for treatment are generally similar to CQ, the most common being nausea, vomiting, abdominal pain, diarrhoea and itching. There is some evidence that itching may be less common with AQ than CQ.

Amodiaquine can induce toxic hepatitis and fatal agranulocytosis following its use for prophylaxis. Twenty-three (23) cases of agranulocytosis were reported in UK, USA and Switzerland in 1986, most of whom were males over 40yrs. Nearly all these patients had used AQ at a dosage of 400mg weekly and the periods of exposure ranged from 3-24weeks. In the UK, the incidence of serious adverse reactions was 1:1700 following malaria prophylaxis, blood disorders occurred in 1:2200 travelers and serious hepatic disorders in 1:15650 travelers. However, the toxicity of AQ appears to be less when the drug is used for the treatment of malaria in malaria endemic countries (Olliaro et al. 2002). The toxicity seems to be related to the immunogenic properties of the quinone imine produced by autoxidation of the parent drug. There have been no reports of cardiovascular symptoms following overdosage with AQ as occurs with CQ. However large doses of AQ have been reported to produce syncope, spasticity, convulsions and involuntary movements. A systematic review of trials of AQ collated convincing evidence that it is superior to CQ, even in areas with CQ resistance (Olliaro et al. 2002). No haematological or clinical adverse effects were noted in this study in which AQ was used for IPTi (Massaga et al. 2003).

**2.8 Possible alternative drugs or drug combinations for IPT**

- - 1. **Amodiaquine and sulphadoxine-pyrimethamine combination**

Amodiaquine and SP have reasonably similar pharmacokinetic profiles with varied modes of action on different biochemical targets in the parasite and are technically suitable candidates for combination therapy. Their high but not total clinical efficacy when used separately has been demonstrated in studies conducted in CQ resistant zones of Africa (Muller et al. 1996; Ringwald et al. 2000), and their side effects are well documented. The efficacy of a combination of a 4-aminoquinolone drug, CQ or AQ to SP has been reviewed. The results of this review suggested that the addition of CQ or AQ+SP marginally improved parasitological clearance as compared to SP alone, the small numbers and size of the trials undertaken however means this conclusion should be treated with caution (McIntosh and Greenwood, 1998). Later studies carried out in Uganda and Cameroon demonstrated that a combination of SP+AQ significantly reduced the rate of clinical and parasitological failures when used for treatment of uncomplicated malaria in children as compared to monotherapy with either drug (Staedke et al. 2001; Basco et al. 2002); children treated with this combination also had a marked reduction in subsequent episodes of malaria, as opposed to those treated with SP alone or a combination of SP+AS (Dorsey et al. 2002). Schellenberg and others (2002) also found the AQ+SP combination to be safe and to be associated with a reduced incidence of gametocytemia (AQ+SP 12.6% vs SP 29.9%, p=0.001). In a study to compare the efficacy of SP monotherapy with combinations of SP and either CQ or AQ for the treatment of uncomplicated malaria in patients over 6 months of age in Uganda, Gasasira and colleagues (2003) found the SP+AQ combination to be most efficacious: clinical treatment failure after 14 days occurred in 21/140 (15%, 95%CI, 9.5-22) among SP treated patients, in 11/152 (7.2%, 95%CI, 3.7-12.6) in SP+CQ treated patients but in 0/136 (0%, 95%CI 0-2.7) patients treated with SP+AQ. In a recent review by McIntosh (2002), it was concluded that in areas where CQ or AQ are still effective, despite some degree of resistance, using these drugs in addition to SP will improve sustained parasite clearance. Apart from the clinical benefits of this combination, it is available, affordable and also has the prospect of delaying resistance to either drug. Thus AQ+SP is a potential candidate for use for IPTi.

**Table 5: Advantages and disadvantages of alternative drugs or combinations**

| **Drugs** | **Disadvantages** | **Advantages** |
| --- | --- | --- |
| Amodiaquine plus sulphadoxine | Has some degree of resistance to *P. falciparum.* | Significantly reduce rate of clinical and parasitological failure;  Reduce subsequent episodes of malaria;  It is available and affordable. |
| Chlorproguanil plus dapsone (Lapdap) | Full therapeutic course requires drug administration over a period of 3 days; fall in Hb of children G6PD deficiency. | Cheap;  Its short action will retard the chance of inducing resistance. |
| Pyrimethamine plus dapsone (Maloprim) | Can cause agranulocytosis;  Upper respiratory tract infection. | Its short action will retard the chance of inducing resistance. |
| Proguanil plus dapsone | Not registered in most countries in Africa. | Its short action will retard the chance of inducing resistance;  Has high in vitro activity against *P. falciparum.* |
| Artemisinins-based combinations | Full therapeutic course requires drug administration over a period of 3 days;  Expensive. | Its gametocidal activity has the potential of reducing transmission of malaria; Artemicinin-based combinations can retard development of resistance to partner drug. |
| Mefloquine | Isolates of in vitro resistance in areas where it has not been used previously;  Provides a prolonged period of prophylaxis therefore, failure to  *P. falciparum* can easily emerge; Induces vomiting and neuropschiatric complications. | Active against *P. falciparum* that are resistant to SP, CQ and AQ;  Required only a single dose. |

- - 1. **Mefloquine**

Mefloquine, a blood schizontocide, has proven to be effective in anti-malarial chemotherapy. It is active against strains of *P. falciparum* that are resistant to SP, CQ or AQ and it is likely to be highly effective in areas with high resistance to SP. Mefloquine has not been adopted by public health systems in Africa because of its cost, but it is used widely in the private sector in many African countries. There have been relatively few formal trials of mefloquine in African children. In Nigeria, a 100% parasitological cure rate was found in children with clinical malaria treated with mefloquine at a dose of 25 mg/kg (Sowunmi et al. 1995). However, isolates showing in vitro resistance have been detected in some areas of Africa where mefloquine had not been used previously (Mockenhaupt et al. 1995) and some treatment failures were observed when a dose of 15 mg/kg was used to treat children with malaria in Malawi (MacArthur et al. 1998). Potential advantages of mefloquine as a drug for IPT are the requirement for only a single dose and the fact that it provides a prolonged period of prophylaxis. The main side effects of mefloquine are vomiting and neuropsychiatric complications; the prevalence of the latter varies substantially from situation to situation but is generally believed to be in the region of 1:200 - 1000 when mefloquine is used for treatment (WHO/CDS/RBM, 2001). Most information on the side effects of mefloquine has come from studies of its use for chemoprophylaxis in travelers. In a study of 500 children under 5 years of age in Thailand, it was observed that the dose of mefloquine 25 mg/kg was well tolerated (Luxemburger et al. 1996). These investigators also reported no serious toxicity and no neuropsychiatric side-effects in the study children (Luxemburger et al. 1996). Mefloquine is licensed for treatment of infants above the age of two months; there are no specific contra-indications for its use in children younger than this.

- - 1. **Chlorproguanil-dapsone (Lapdap)**

Chlorproguanil in combination with dapsone, acts sequentially and synergistically to inhibit the enzymes dihydropteroate synthetase and dihydrofolate reductase. Its efficacy has consistently been demonstrated (Keuter et al. 1990; Trigg et al. 1997; Amukoye et al. 1997; Winstanley et al. 1993; Curtis et al. 1998; Maxwell et al. 1999), even in cases of SP-resistant infections (Watkins et al. 1997; Nzila et al. 2000). A rapidly eliminated combination like Lapdap (half lives of 13hrs and 26hrs respectively), is expected to be less likely to induce antifolate resistance than SP, though it may have little or no chemoprophylactic effect.

Lapdap (chloroproguanil/dapsone) is a new anti-folate anti-malarial which has recently been through a full clinical evaluation. This product was however only recently registered in the UK. Studies conducted in Kenya in the 1980s and 1990s showed that this short acting drug combination was safe and effective. Clinical trials of Lapdap have recently been conducted in 1480 children in five African countries as part of the regulatory process. In each country, Lapdap was more effective than SP and in Kenya, Gabon and Malawi the difference between groups was statistically significant (Kanyok et al. 2001; Sulo et al. 2002). Detailed safety observations were made; minor side effects were recorded frequently (some of these may have been due to the underlying malaria infection). Falls in Hb in children who were glucose-6-phosphate dehydrogenase (G6PD) deficient were recorded more frequently in children who received Lapdap than in those who received SP but no other significant side effects were recorded. In a separate open label study conducted in infants aged 3 – 12 months the drug was again shown to be safe and effective (Kanyok et al. 2001).

A number of trials with Lapdap have been conducted in Tanzania (Trigg et al. 1997; Mutabingwa et al. 2001). One of the most important trials conducted, showed that 41/43 children who had failed treatment with SP responded fully to treatment with Lapdap (Trigg et al. 1997) Thus, Lapdap is likely to be effective when used for IPT in areas of Africa where levels of SP and AQ resistance are high.

Advantages of Lapdap as a potential drug for use in IPT are that it is likely to be cheap and that its short action will diminish the chances of inducing resistance; its disadvantage is that a full therapeutic course requires drug administration over a period of three days and there will be no chemoprophylactic effect.

- - 1. **Pyrimethamine-dapsone (Maloprim)**

Pyrimethamine acts synergistically with dapsone to inhibit folate synthesis. Use of Maloprim for chemoprophylaxis among travelers has been associated with agranulocytosis, especially when taken in high doses (Friman et al. 1983; Bruce-Chwatt, 1984). A review of 21 cases of agranulocytosis associated with pyrimethamine-dapsone concluded that, although agranulocytosis can occur very rarely in patients taking pyrimethamine or dapsone alone, agranulocytosis due to the combination appears to be caused by an idiosyncratic reaction to dapsone, exacerbated by the concurrent administration of pyrimethamine (Hutchison et al. 1986). A higher incidence of non-specific upper respiratory tract infection was also observed in military recruits receiving the combination as prophylaxis (Lee et al. 1988). Chemoprophylaxis with Maloprim was highly effective in Gambian children and resulted in favourable pregnancy outcomes, with no evidence of toxicity (Greenwood et al. 1989; Menendez et al. 1994). However, dapsone has a short elimination half-life and this combination may have little chemoprophylactic effect in areas where there is a high level of resistance to pyrimethamine since chemoprophylatic effect would be due to pyrimethamine alone.

**2.8.5 Proguanil-dapsone**

This antifolate-sulphone combination acts sequentially and synergistically to inhibit the enzymes dihydropteroate synthetase and dihydrofolate reductase. Proguanil-dapsone was used extensively as prophylaxis by Australian soldiers during the Vietnam conflict and was then demonstrated to be an effective chemoprophylactic (Black, 1973). Later studies combining proguanil with short-acting sulfonamides showed that the combination was effective in preventing malaria infections (Edstein et al. 1997). Shanks and others (1992) however did not find the combination to be effective in Thai soldiers and this has been attributed to both parasite resistances to cycloguanil and to low cycloguanil concentrations in Thai soldiers (Edstein et al. 1997). It has been reported that cycloguanil still exerts high in vitro activity against most *P.falciparum* isolates from Western and Central Africa (Basco et al. 1995), and will retain efficacy as SP failure emerges. In a trial to test the therapeutic efficacy of proguanil-dapsone in treating asymptomatic *P. falciparum* infections in north-east Tanzania, Mutabingwa and others (2001) found the combination to be well tolerated and to achieve parasite clearance in 93.5% of the children. Being short-acting, it is likely to have little or no chemoprophylactic effect.

**2.8.5 Artemisinin combinations**

The artemisinin derivatives are the most potent and rapidly acting anti-malarials by reducing parasite biomass and resolution of symptoms (White et al. 1999). They reduce the risk of transmission through their gametocytocidal activity. They are active against multi-drug resistant *P. falciparum* malaria, are well tolerated by the patients and reduce gametocyte carriage thus have the potential to reduce transmission of malaria (Price et al. 1996; Targett et al. 2001). There is currently no evidence for clinically relevant artemisinin resistance (Meshnick, 2002). If used alone, the artemisinin derivatives will cure falciparum malaria in seven days, but studies have shown in south-east Asia that combination of the drug with certain synthetic drugs produce high cure rates in just three days of treatment (Allin et al. 1996; von Seidlein et al. 2000; Adjuik et al. 2002). There is also evidence that use of such combinations can greatly retard development of resistance to the partner drug. However, the artemisinins are relatively expensive.

Currently, there is a strong move towards the use of combination therapy for the treatment of clinical malaria, in particular combinations involving artemisinins (Nosten et al. 2002).

This can also be considered as an alternative to SP for IPT. Mefloquine has already been used widely in combination with artesunate in South Eastern Asia and shown to be effective and safe and the use of this combination may have slowed down the rate of development of mefloquine resistance. Amodiaquine is still superior to chloroquine even in areas of CQ resistant (Olliaro et al. 2002), no haematological or clinical adverse effects have been noted (Massaga et al. 2002). Thus introduction of AQ+AS or mefloquine+AS for IPTc would be unlikely to raise any major safety issues. Lapdap has not yet been studied in combination with artmisinins and so this combination could not be used for IPTc at this time.

## 3.0 STUDY OBJECTIVES

- 1. **Hypothesis**

The hypothesis for this study is that the combinations of AQ+AS for intermittent treatment of children under five years will be safe and improve Hb level as compared to mono therapy using SP or no therapy at all and that this is an effective malaria control tool for use in Ghana.

- 1. **Primary objective**

To investigate whether IPTc using AQ+AS can improve mean Hb level and decrease anemia and malaria morbidity in young children in an area of intense and prolonged seasonal malaria transmission.

- 1. **Secondary objectives**

1. To compare the prevalence of anaemia (Hb<11.0g/dl, moderate (Hb<8.0g/dl>5.0g/dl) and severe (Hb<5.0g/dl) anaemia among children aged 3-59 months using the four treatment dose regimen.
2. To compare the incidence of severe malaria [(presence of asexual parasitaemia + repeated generalized convulsions or impaired consciousness or hyperpyrexia (axillary temperature >39.5°C) and the incidence of clinical malaria (history of fever during previous 2 days or axillary temperature >37.5ºC) + parasitaemia of any density + absence of any other obvious causes of fever] among children aged 3-59 months using the four treatment dose regimen.
3. To determine the prevalence of molecular markers of resistance to SP among children who have malaria at the end of the intervention.
4. To compare the prevalence of malaria parasitaemia and gametocytes levels among children aged 3-59 months using the four treatment dose regimen.

## 4.0 MATERIALS AND METHODS

## 4.1 Study site and characteristics

Malaria is hyperendemic in Ghana with crude parasite rates ranging between 10%-80%.

In Ghana the main malaria parasite species are *P. falciparum* (80%-90%), *P. malariae* (~20%) and *P. ovale* (<1%). The principal vectors of malaria are *Anopheles gambiae (An. gambiae)* complex and *An. melas* found in swampy areas, *An. funestus* and *An. arabiensis* found in the northern savannah regions. *An arabiensis* and g*ambiae* are found in the tropical rain forest areas. In Ghana, Armarh et al. (unpublished) have estimated the transmission intensity is 100-1000 infective bites per person per year.

Malaria is a focal problem in Ghana as in the whole of sub-Saharan Africa. It is the most frequent cause of morbidity and mortality according to hospital data and forms 45% of all out patient attendances. Malaria has accounted for about 36% of patients admitted to hospital for the past ten years. It is the leading cause of childhood mortality and is responsible for approximate 25% of all childhood deaths. Children under five years of age suffer the most from the morbidity and mortality predominantly from *P. falciparum* species.

## Recently, a rapid spread of, P. falciparum parasites resistant to the most widely available, and cheapest anti-malarial, CQ has occurred in Ghana with a current treatment failure rate of 20%-25% (Koram et al. 2000). As a result it’s effectiveness as the first line drug has been markedly reduced in most malaria endemic areas.

The study will be carried out in Hohoe district, one of the twelve administrative districts in the Volta region. The district is located in the central part of the Volta Region with a population of 147,291 people according to the year 2000 population census. It covers an area of 1172sq. Km. and it has been divided into six sub districts.

The district is made up of both high and lowland areas. The highland areas where the highest mountain in the country is found, makes some settlements hard-to-reach. The vegetation of the district is mainly forest and forest savanna. There are two main seasons, the wet and dry seasons. The major wet season lasts from April to July and the minor one from September to November. The rest of the year is relatively dry. The average recorded annual rainfall in the district is 1,548-mm with approximately 1,296-mm rain recorded from April to October.

The district is drained by rivers Dayi and Kloloenu and also has three water falls namely: Wli, Agumatsa and Tsatsadu. The south Western part of the district is bounded by the Volta lake. The southern part of the district has a monkey sanctuary in a forest reserve. The major economic activities are farming and trading. However, there are also minor economic activities like pottery, wood carving and cattle rearing. There are six main ethnic groups; the Ewes, Likpes, Akpafus, Lolobis, Santrokofis and Logbas who speak different languages.

#### Figure 1: Map of Ghana showing Hohoe District

**
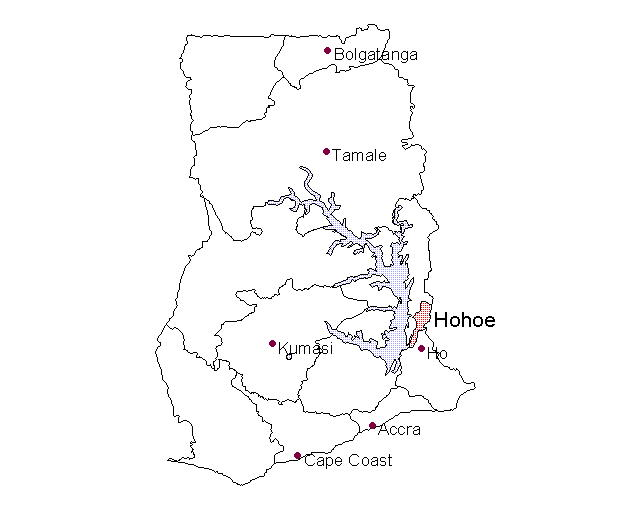
**

Malaria remains a major disease problem in the district and it is the first among the top ten diseases. For the past four years malaria has accounted for 32% of the total Out Patient Department (OPD) attendances out of which 45% were children under five years. Twenty five percent (25%) of malaria cases required inpatient care and 37% of the cases were children under five years and 57% of them report with anaemia. The peak of OPD malaria cases occurs between June and October. Figure 2 shows the correlation between reported malaria, anaemia cases and rainfall pattern in the study area.

**Figure 2:**

In the study area, malaria transmission occurs throughout the year with seasonal peaks, coinciding with the period of the rains as shown in figure 2.

The district has been one of the 20 RBM selected districts for malaria control programmes and has been involved in ITNs distribution to pregnant women and mothers with children under five years since 2002. A survey involving pregnant women and mothers with children under five year carried out (Kweku et al. unpublished) in the district in March 2004 showed that about 13.3% of households surveyed own at least one bednet out of which only 2.8% were ITNs. Only 7.4% of children under five years slept under a bednet the previous night and 4.2% slept under ITNs. The survey also found that bednets are used mainly in the rainy season when mosquitoes bother people most and nets are packed when the weather is warm or in the dry seasons when they do not feel the presence of mosquitoes.

**4.1.1 Health Infrastructure**

The district has one hospital that is situated at Hohoe, the district capital. It provides the entire district with secondary care as well as being a referral point for the health centres. The health centres are geographically located so as to provide easy access to the population. The services provided in the district include clinical care, reproductive health and child health services. Complicated cases are referred to the district hospital.

There are 40 static reproductive and child health clinics attached to the health facilities. Ninety six (96) outreach child health services are provided in the communities in the district every month. Community health nurses travel to these communities and organize EPI and child welfare sessions at a common and convenient place selected by the community. Growth monitoring to determine the nutritional status of children between 12-23 months is done at outreach child welfare stations.

**5.0 STUDY METHODOLOGY**

# 5.1 Study design

The main trial is a double blind, randomised; placebo controlled trial of IPTc with three treatment regimens AQ+AS every month, AQ+AS every two months or SP given every two months. The study will measure the impact of IPTc on Hb level and malaria morbidity among children aged less than five years.

A cohort of children aged between 3-59 months will be recruited and observed for 6 months during the high malaria transmission season from May to October. The key steps and the arms of the study are shown in figure 3.

The trial will have two subsidiary studies whose purpose is to support the main study. The first one will be conducted alongside the main study. This will be a study of the effectiveness of the drugs employed for IPTc using a modified WHO *in vivo* treatment study among children 3-59 months who will report to a health facility within the district with uncomplicated malaria. The second subsidiary study will investigate rebound effect in children who participated in the main study the previous year.

**Figure 3:** **The main study design**

**Contact 0**: 1. Assess for eligibility

2. Cross-sectional survey

**Contact 1** Random allocation to one of the study groups and administration of the first course of treatment.

M1 (May 2005)

M2-M6 (June-October 2005)

Group GW

AS+ AQ every month x6.

Passive surveillance at health facilities for clinical malaria, severe anaemia and malaria.

M7 (Nov. 2005

**Contact 2 to 6**: Continue with the intervention and home visit for adverse events on day 7.

Group GX As+AQ every 2 months x3 and placebo every 2 months x3

Group GY:

SP every 2 months x 3 and placebo every 2months x3

Group GZ:

Placebo every month x 6

**Contact 7**: Cross-sectional survey;

Collection of blood samples for Hb, blood film and filter paper.

Month 0 (M0)

(April 2005))

**Figure 4. Schedule for monitoring IPTc intervention**

**Passive surveillance**

**Drug administration**

Day 1 Day1 Day 1 Day1 Day 1 Day 1

D2 D3 D2 D3 D2 D3 D2 D3 D2 D3 D2 D3

**Cross-sectional survey Survey Survey Survey**

**Apr. May Jun. Jul. Aug. Sept. Oct. Nov. Dec. Jan. Feb. Mar. Apr. May Jun. Jul. Aug. Sept. Oct. Nov.**

**(2005) (2006)**

**5.2 Study population**

All children aged between 3 months and less than five years resident in the district will be eligible for inclusion in the study.

**5.3 Inclusion criteria**

- Children between the ages of 3-59 months in the selected communities;
- Children resident in the study area and likely to be available for follow-up for 6-12 months;
- Consent by parent /guardian of child;
- Absence of severe malnutrition, chronic diarrhoea or history of convulsions;
- Absence of prostration, extreme weakness (inability to stand or sit) at the time of enrollment;
- No history of AQ, SP or AS intake within the past two weeks;
- Absence of history of hypersensitivity to any of the study drugs.

Children will be excluded if they fail to meet any of the above criteria.

**5.4 Trial end-points**

*Primary end-point*

1. Incidence of Hb<8.0g/dl between placebo, monthly and every two months ITPc during the period of intervention.

*Secondary end-points*

1. Incidence of severe anaemia (Hb<5.0g/dl), moderate anemia (5 g/dl=<Hb<8.0g/dl) and mild (8 g/dl=< Hb < 11.0 g/dl) during the period of the intervention;
2. Incidence of severe and clinical malaria during the period of the intervention;
3. Prevalence of anaemia (Hb<11 g/dl), moderate and severe anaemia at the post intervention survey;
4. Prevalence of parasitaemia and gametocytes at the post intervention survey;
5. Prevalence of molecular markers of resistance to SP among children who have malaria at the post intervention survey.
6. Prevalence of G-6-PD deficiency and haemoglobin genotype C and S

#### 5.5 Calculation of sample size calculations

The sample size calculations using EPI info version 6

**5.5.1 Sample size estimates for measurement of prevalence of** Hb<8.0g/dl

A recent study in the Hohoe district by Kweku and others showed that SP given monthly reduced anaemia that required treatment (Hb<8.0g/dl) from 10.3% to 5.3%. This was reduced from 15.2% to13.3% in the control group. Haemoglobin Hb<7.0g/dl was reduced from 4.9% to 1% in the treatment group. This was also reduced from 8.9 to 4.1% in the control group

We therefore, assume that, children in the SP given bimonthly group in this study will also have their Hb<8.0g/dl reduced to 7.3% over that seen in the placebo group 13.3% at the end of the intervention. Artesunate plus AQ has a shorter half-life than SP and it is assumed that although high levels of parasite clearance will be achieved with this drug combination, this might not be sustained. We assume that if AQ+AS is given every two months, it will also reduce Hb<8.0g/dl to 4.0% and if it is given more frequently, every month, it will reduce Hb<8.0g/dl to 3%. We will therefore assume that the difference in the prevalence of Hb<8.0g/dl between AQ+AS monthly versus every two months will be 1% and the difference between AQ+AS given monthly versus SP will be 4.3%.

**Table 6a:** Sample size calculation for Placebo versus SP, AQ+AS monthly and every two months

| Study arm | Power | Significance  Level | number required  per arm | Allowing for 10%  loss to follow- up |
| --- | --- | --- | --- | --- |
| SP | 80% | 5% | 434 | 477 |
|  | 90% | 5% | 570 | 627 |
| AQ+AS monthly | 80% | 5% | 128 | 141 |
|  | 90% | 5% | 165 | 182 |
| AQ+AS bimonthly | 80% | 5% | 163 | 179 |
|  | 90% | 5% | 211 | 232 |

**Table 6b:** Sample size calculation for SP versus monthly and every two months dose of AQ+AS

| Study arm | Power | Significance  Level | Number required  per arm | Allowing for 10%  loss to follow- up |
| --- | --- | --- | --- | --- |
| AQ+AS) monthly | 80% | 5% | **459** | **505** |
|  | 90% | 5% | 599 | 659 |
| AQ+AS) every two months | 80% | 5% | 827 | 910 |
|  | 90% | 5% | 1086 | 1195 |

**Table 6c:** Sample size calculation for AQ+AS given every two months versus every month

| Power | Significance  level | Number required  per arm | Allowing for 10%  loss to follow- up |
| --- | --- | --- | --- |
| 80% | 5% | 5499 | 6050 |
| 90% | 5% | 7294 | 8023 |

Four hundred and fifty-nine (459) children aged between 3-59 months will be required in each arm to detect a minimum of 3.3% difference in the prevalence of Hb<8.0g/dl among children in the SP and AQ+AS study groups with 80% power at the 5% significance level. This sample size provides a power of 52% of detecting a significant difference in the prevalence of Hb<8.0g/dl between SP vs AQ+AS every two months and 9.4% power for a difference in prevalence of Hb<8.0g/dl between AQ+AS every month versus AQ+AS every two months.

**5. 5.2 Sample size estimate for peripheral parasitaemia**

A study carried out in the district showed the prevalence of parasitaemia in children aged between 9 to 23 months was 29.5% and this was reduced to 15% prevalence at the end of the intervention using SP. Assuming a prevalence of peripheral parasitaemia of 30%, in the placebo group, we predict that SP will reduce this to 15%, AQ+AS given every two months will reduce the prevalence to 10% and AQ+AS given every month will reduce the prevalence of parasitaemia to 7%.

The calculations using the equation (Peter G. smith and Richard H. Morrow): field trials of health interventions in developing countries (2^nd^ ed.).

Where:

Sample size n= 459

Significance level z1=0.05 (1.96)

Z2= power

Z2={√[n/(r1+r2)}( |r1-r2|)-z1

SP versus AQ+AS every month

Z2= {√[459/(0.15+0.07)} ( |0.15- 0.07|)-1.96

Z2= {√[459/(0.22)} ( |0.15-0.07)-1.96

Z2= {(45.7)} (|0.08|)-1.96

Z2= 3.65-1.96 =1.69

Z2= 1.69 = 95.5%

Corresponding value for power is 95.5%

The sample size (459) for the prevalence of peripheral parasitaemia will provides a power of 95.5% for SP versus AQ+AS every month, 57.9% for SP versus AQ+AS every two months and 34.5% for AQ+AS every month versus AQ+AS every two months.

**5.5.3 Sample size estimate for prevalence of anaemia**

The study carried out in the district also showed that the prevalence of anaemia was reduced in the SP group from 77% to 56%. Assuming the prevalence of anaemia in the district is 77% in the placebo group, we predict that it will be reduce to 56% using SP. This will further be reduced to 50% using AQ+AS given every two months and to 40% with AQ+AS given every month.

Using the same sample size, for the prevalence of anaemia provides a power of 93.3% for SP versus AQ+AS every month 24.2% for SP versus AQ+AS every two months and 61.8% for AQ+AS every month versus AQ+AS every two months. Although the sample size does not provide enough power in detecting a significant difference between SP versus AQ+AS every two months and between the two combination treatment regimens, the same sample size provides a power of 99.9% in detecting a significant difference between placebo versus AQ+AS every two months and AQ+AS every month.

This number will therefore be adequate for detecting a significant difference in a 50% reduction in the prevalence of parasitaemia and a 25% reduction in the prevalence of anaemia between SP versus AQ+AS every month.

For the comparison of the primary outcome measure, the highest number of children aged between 3-59 months needed for the comparison of incidence of Hb<8.0g/dl between SP AQ+AS every two months and AQ+AS every month groups is 459.

Allowing for loss to follow up, 505 children in each arm to detect a minimum of 3.3% difference in the incidence of Hb<8.0g/dl between SP, AQ+AS every two months and AQ+AS every month study group with 80% power at the 5% significance level. A total of 1836 children would be needed for the study. Allowing for 10% loss to follow-up 2020 (506 per arm) are required. This number will be adequate to detect a difference in a 50% reduction in the incidence of clinical malaria and a 25% reduction in the incidence of Hb<8.0g/dl every month and every two months treatment dose of AQ+AS and SP groups.

**5.6 Expected enrollment**

Assuming an average of 82 children will be found in each village selected, a total of 2460 children will be selected from the 30 villages.

**5.7 Population Sampling and allocation to treatment group**

This will be done by a random selection of five villages from each sub-district in the Hohoe district through balloting to get a total of 30 villages. All children aged 3–59 months in the selected communities will be screened for general health status in April 2005. Children in good general health will be selected from each of the villages. Children enrolled will later be randomly allocated to the to four treatment groups.

###### How randomisation will be done

The selection of the communities will take place at Hohoe the district capital by writing the names of all villages in each sub-district and by casting ballots. Later in the communities during enrollment, twelve numbers representing groups 1 to 12 will be written on pieces of paper, rolled and mixed up for the mothers/guardians to pick for their children. The child will belong to the group the mother or guardian has chosen throughout the study period.

- 1. Community acceptance

Once villages have been chosen, several meetings with all potential partners (health authorities, community leaders and care givers) will be held. Meetings will be held with the district health directorate staff and assembly members from the district administration. We will then hold meetings with the chiefs and elders, opinion leaders and parents in the villages selected. Explanations will be provided to opinion leaders and parents in local languages, emphasising the potential public health impact of the study for malaria control.

- 1. **Staff recruitment and training**

Prior to the start of the study, field workers and health staff including medical assistants, nurses and laboratory assistants working in the district will be identified. Parents in the communities will also assist in selecting volunteers who will serve as coordinators in each selected village. A two-week training session will be organised for the health staff and the coordinators to highlight the importance of their role in the study and how it relates to that of others. At the training, they will be encouraged to do their best to ensure high quality work. All health staff and coordinators will be paid an incentive throughout the study period. The nature of this will be discussed with them during the training.

- 1. **Cross-sectional surveys**

Two cross-sectional surveys will be undertaken in April and November 2005. The first survey will be the pre-intervention survey which will provide baseline information about the study children. Information to be collected will include the background information about the child and mother/guardian, history of allergy to any of the study drugs, bednet ownership and measurement of anthropometric indices The second one will be a post- intervention survey which will involve collection of finger prick or venous blood samples for determination of asexual and sexual parasitaemia, Hb, filter paper for resistance markers to SP, G-6-PD deficiency and HB C and S, measurement of temperature and anthropometric indices. Information gathered will be compared among the groups.

Data from the second cross-sectional survey of parasitaemia (parasite per micro litre) and fever (temperature ≥37.5°C) among the recruited children in all groups will be analysed. This will be used to determine the cut off point for case definition of malaria parasites in the study area.

- 1. **Enrolment and follow up procedures**

The research team will visit the selected villages in April 2005. Children aged 3-59 months who will reside in the communities within the district for at least 7months (**contact 0**) will be examined and history taken to assess their eligibility for enrolment into the study. The study objectives and procedures will be explained to caretakers of the children who are eligible and they will be given an information sheet to take home. After obtaining written consent, the following procedures will be carried out: (1) collection of ID card (see appendix 5); (2) completion of an enrolment form (see appendix 3) which will contain information on identification, address and other details useful for follow up at home, socio-economic status, use of bednets and other malaria prevention measures, clinical history and signs and anthropometric indicators.

The coordinators in the villages will visit the homes of these children and remind them about the date and venue of the next visit of the research team.

When the study children return for the next meeting in May 2005 (contact 1) the following procedures will be carried out: (1) randomized allocation of study participants into the four arms of the study through balloting; (2) administration of the first dose of the study drug and dispensation of the remaining two doses of drug to be given in the presence of a study coordinator; (3) advice given to the caretakers to bring their child to the health facility if the child becomes ill before the next scheduled visit.

When the study children meet the research team the following month (June) (**contact 2**) the following procedures will be carried out: (1) administration of the first dose of the second course of the study drugs and dispensation of the remaining two doses of drugs to be given in the presence of a study field coordinator; (2) advice given to the caretakers to bring their children to the health facility if the child becomes ill before the next scheduled visit.

When the study children attend the meeting in July (**contact 3**) the following procedures will be carried out: (1) administration of the first dose of the third course of the study drugs and dispensation of the remaining two doses of drugs to be given in the presence of a study field coordinator; (2) advice given to the caretakers to bring their children to the health facility if the child becomes ill.

When the research team meet the study children in August (**contact 4**) the following procedures will be carried out: (1) administration of the first dose of the fourth course of the study drugs and dispensation of the remaining two doses of drugs to be given in the presence of a study field coordinator; (2) advice given to the caretakers to bring their children to the health facility if the child becomes ill.

When the research team meet the study children in September (**contact 5**) the following procedures will be carried out: (1) administration of the first dose of the fifth course of the study drugs and dispensation of the remaining two doses of drugs to be given in the presence of a study field coordinator; (2) advice given to the caretakers to bring their children to the health facility if the child becomes ill.

When the study children turn up for the meeting in October (**contact 6**) the following procedures will be carried out: (1) administration of the first dose of the sixth course of the study drugs and dispensation of the remaining two doses of drugs to be given in the presence of a study field coordinator; (2) advice given to the caretakers to bring their children to the health facility if the child becomes ill.

When the study children meet the research team again in November (**contact 7**) the following procedures will be carried out: (1) a finger prick blood sample (400 μl) will be collected for determination of Hb, blood film for malaria parasites and filter paper for determining genetic markers for resistance to SP; (2) advice given to the caretakers to bring their children to the health facility if the child becomes ill.

- 1. **Randomisation and allocation of study groups**

The randomisation code will be computer generated after selecting the villages. The randomisation unit will be the child; the study code will be sealed in an envelope and stored securely by an experienced clinician who will be the clinical monitor or local safety monitor (LSM) for the trial in April 2005.

Children will be allocated to one of the study groups by balloting. Each study drug, including placebo, will be assigned 3 group codes; there will be 12 study groups in total in each study site or village. Neither parents nor investigators will be aware of study drug allocation. Active drugs or placebos will be identical in shape and colour. The tablets will be pre-packaged in small plastic bags according to randomisation group and weight of recipients. The code for the groups will be broken in November 2006 when follow up for rebound effect of the intervention is completed.

- 1. **Dose schedule and administration of study drugs**

Drug administration will be carried out in the communities. The research team will visit the selected communities every month to supply drugs to the coordinators and supervise administration of the first dose. Before the visit to the communities the coordinators will be informed about the day on which this is to take place. This will enable them organize the participants who will assemble at a common place.

All coordinators in the community will be supplied with drugs for their respective groups. Subsequent doses will be given and observed by the coordinators. The standard dose of AS (4mg/kg), AQ (10mg/kg) and SP [sulfadoxine (25 mg/kg) + pyrimethamine (1.25 mg/kg)] for infants and young children tablets will be crushed, mixed with water and administer orally to the children. AQ+AS will be given for three days. SP will be taken for one day and will be followed by placebo for two days. Those in the placebo group will take placebo for three days. One group will receive active drug every month for six months. Active drug will be administered in May, July and September for two groups and one group will receive placebo through out the six months of the transmission season.

The tables below are showing the dose schedule of study drugs throughout the intervention period in each treatment arm. Drugs for the intervention will be procured from a manufacturer with GMP in the country.

**Table 7a: Dosing schedule of study drugs for M1, M3 and M5**

| **Study**  **arms** | **Drug** | **Day 0** | **Day 1** | **Day 2** |
| --- | --- | --- | --- | --- |
| 1 | Placebo | PAQ+AS | PAQ+AS | PAQ+AS |
| 2 | SP (Sulphadoxine 500mg+ pyrimethamine 25mg tablets) | SP | PSP | PSP |
| 3 | AQ+AS (AS 50mg tablets+ AQ 150mg base tablets) every month | AQ+AS | AQ+AS | AQ+AS |
| 4 | AQ+AS (AS 50mg tablets+ AQ 150mg base tablets)  bimonthly | AQ+AS | AQ+AS | AQ+AS |

**Table 7b:** Dosing schedule of study drugs for M2, M4 and M6

| **Study arms** | **Drug** | **Day 0** | **Day 1** | **Day 2** |
| --- | --- | --- | --- | --- |
| 1 | Placebo | PAQ+AS | PAQ+AS | PAQ+AS |
| 2 | SP (Sulphadoxine 500mg+ pyrimethamine 25mg tablets)bimonths | PSP | PSP | PSP |
| 3 | AQ+AS (AS 50mg tablets+ AQ 150mg base tablets) every month | AQ+AS | AQ+AS | AQ+AS |
| 4 | AQ+AS (AS 50mg tablets+ AQ 150mg base tablets)  bimonthly | PAQ+AS | PAQ+AS | PAQ+AS |

**SP = Sulphadoxine-Pyrimethamine**

**AS = artesunate**

**AQ = amodiaquine**

**PSP = Placebo simulating SP**

**PAQ+AS =Placebo simulating AQ +AS**

- 1. **Monitoring compliance and safety**

Participants will be visited at home 7 to10 days after every drug administration by one of the 12 field workers from May to October 2005. During these visits, the field workers will find out those who could not complete or take their drugs. Mothers and care takers will be interviewed, asked about any adverse event after drug administration or any episode of illness during the past one week. An adverse event record form (see appendix 9) will be completed for all children who will report any form of reaction after drug administration for assessment by the study clinician. They will then be reminded to report to the nearest health facility if the child is sick after the visit.

The coordinators will visit participants in their groups once a week to collect information on history of fever and vomiting or any morbid episode and advice those who complain of fever or vomiting within the past 48 hours to go to the nearest health facility for examination and treatment (see appendix 8).

- 1. **Surveillance of clinical malaria**

All study children will be encouraged to attend the health facilities in their catchment areas for any illness. At these visits a health questionnaire will be completed and temperature recorded using an electronic thermometer. If the child has fever or any features suggestive of malaria, a finger prick blood sample will be collected for Hb and malaria parasite detection. Two slides of a thick blood film will be prepared for each child. At the health centre, one slide will be examined on the same day for deciding on appropriate treatment. The second slide will be kept and sent to the central laboratory for reading to provide a definitive diagnosis.

Children with proven or presumptive malaria will be treated with oral quinine according to the Ministry of Health (MoH)/ Ghana Health Service (GHS) treatment guidelines of malaria using a dosage of 10mg/kg body weight 8hrly for 7days.

Malaria clinical attacks will be documented and treatment for malaria and any other illness will be provided free of charge to study participants in all the health centres that are in the catchment area of the selected communities by medical assistants and qualified nurses as shown in figure 5.

**Figure 5:** Design for passive surveillance at the health facility

Fever, history of fever or vomiting.

M18 (Oct. 2006)

**Contact10:** Visit children in the communities for follow up for rebound effect

1. Collection of blood sample for Hb, blood film,& filter paper, and complete data forms

2. Complete data forms

.

Malaria parasites present.

Malaria parasites absent: give alternate treatment.

**No:**

Assess for other diseases and treat.

**Yes:**

Blood film for malaria parasites.

Malaria parasites present< 2000 per micro litre of blood: Treat with national recommended drug.

Malaria parasites present≥ 2000 per micro litre of blood and axillary temparature≥37.5°C: Recruit for ancillary 1 study.

- 1. **Laboratory Procedures**

***Blood slides***

The blood slides will be dried and sent to the laboratory at the health facility. The blood films will be stained with 3% Giemsa for 25 minutes and used for identification and quantification of malaria parasites. The prepared slides will be examined under oil immersion with a light microscope (Ocular magnification X 100). The thick film will be used for quantifying malaria parasites while thin film will be used for identifying the malaria species. Parasite densities will be estimated by counting the number of parasites per 200 white blood cells (WBCs) in a thick film.

A sample will be considered negative only after 200 high power fields have been read. Parasite counts will be converted to parasites per µl, with the assumption that there is an average of 8000 leukocytes per µl of blood. In cases where there is more than a 50% discrepancy between parasite counts or when there is a discrepancy qualitatively (negative vs positive), a third microscopist will read the slide. The two closest readings will be used in analysis of parasite density.

All blood slides will be stored in appropriately labeled slide boxes and kept at the Onchocerciasis chemotherapy research (OCRC) laboratory at the district hospital.

***Haemoglobin***

Haemoglobin will be estimated using Hemocue ® Photometer (Leo Diagnostics, Sweden).

Finger prick blood samples will be collected from all study children during the cross-sectional survey for measurement of Hb. However during the 18 months passive surveillance at the health facilities, blood samples for Hb will be collected from children with axillary temperature of ≥37.5°C, history of fever or vomiting for the past two days. Children found to have Hb<5.0g/dl will be referred to the district hospital to receive treatment for anaemia and those who will be transfused will be out of the study.

A laboratory technician from Noguchi Memorial Institute of Medical Research (NMIMR) working at the anti-malaria drug efficacy sentinel site in the district will monitor the quality of the slides and Hb readings through out the study period.

***Filter paper***

Filter paper samples will be collected from every study child during the cross-sectional survey. All filter paper samples will be air-dried in the field, placed in airtight plastic containers with desiccants and sent to the central laboratory for storage at room temperature. Filter paper samples for children found to have peripheral parasitaemia by microscopy at the cross-sectional survey will be sent to the NMIMR in Accra where Polymerase Chain Reaction (PCR) techniques will be used to screen the samples for molecular markers of resistance to SP. Individual data will be tabulated according to the randomisation group, and age.

#### 5.18 Nutritional assessment

The nutritional status of all study children will be assessed in the two cross-sectional surveys. Two well-trained nutrition officers who are currently involved in community nutrition and growth monitoring surveys will perform the anthropometric assessment as follows:

1. Children will be weighed naked on Seca weighing scales (Hamburg, Germany) to the nearest 10 grams;
2. The length of children aged less than 24 months will be measured using a “Harpenden infantometer” to the nearest mm;
3. Children aged 24 months or more will have their height measured while standing using a locally made measuring board precise to 1 mm;
4. Mid-upper arm circumference (MUAC) will be measured on the left arm to the nearest 1 mm using a non-stretchable tape;
5. Triceps and sub scapular skin fold measurements will be taken using Holtain skinfold callipers precise to the nearest 0.2mm.

Length, height and skinfold measurement will be taken twice, and the mean of the two measures used for analysis. In case of discrepancies (a difference between the two measurements in length/height>3mm or a difference between the two measurements of skinfold >0.4 mm), a third measurement will be taken and the outlier discarded.

The accuracy of measuring devices will be checked daily, and a test of intra-observer and inter-observer reliability conducted prior to the onset of the study.

Anthropometric indices (weight for age, weight for height and height for age) will be computed using the Anthro software (CDC, Atlanta) after data entry. Date of birth for all children will be known precisely, given by the computerized database.

**5.19** **Entomological assessment**

Entomological surveys will be carried out in collaboration with the entomological unit of the NMIMR. Entomological inoculation rates (EIRs) will be estimated using light traps. Indoor sampling by light traps fitted with incandescent bulbs and placed close to human volunteers sleeping under an untreated bednet in their usual sleeping place will be used to estimate anopheline biting rates. Light traps will operate from evening 6pm to morning 6am for two consecutive nights in each house and bags will be emptied every morning.

The population will be classified into four geographical clusters (i.e. people living in the same areas such as a village or hamlet in mountainous areas; marshy areas; forest areas close to water falls or along the main rivers or in the forest area and those along the Volta lake).

Two villages will be selected from each of the four ecological zones. Three (3) volunteers from each village (index people) will be recruited into the study (clusters sampled without replacement). This procedure will be repeated four times at an interval of three months to cover all the transmission seasons. The mosquitoes will be sent to NMIMR for speciation and for specialist to carry out an ELISA test for sporozoites.

- - 1. **Outcome measures for entomological survey**

The outcome measures for this study will be;

1. Anopheline biting rate in various geographical zones of the study area.
2. Seasonal variation in the anopheline biting rate.

5.20 Adverse events

- - 1. **Assessments of adverse events**

Adverse events will be defined as any signs or symptoms which first developed or increased in severity within seven days of intake of study medication. Following each drugs administration, an evaluation of the incidence of headache or fever, weakness, abdominal pain, anorexia, nausea, vomiting, diarrhoea, rash, itching and sleep disorder will be undertaken. All children will be visited at home by the research team members and the coordinators between Days 7 to 10 to assess whether they have had any adverse events due to the study drugs. Parents and care takers will be advised to report to the coordinators or to a health facility if they experience any untoward effect of the drugs prior to, or after this home visit.

At subsequent visits for IPTc, they will be asked about potential side effects of the study drugs such as occurrence of rashes since the last assessment.

**5.20.2 Severity**

The severity of any event will be classified as follows:

Mild –Transient or mild discomfort; no intervention or treatment required;

Moderate –Mild to moderate limitation in activity, no or minimal intervention or treatment required;

Severe – Treatment required and, despite treatment, normal daily activities are interrupted;

Serious – Death, hospitalization, prolongation of hospitalization, life-threatening condition, persistent or significant disability.

**5.20.3 Serious and unexpected adverse events reporting**

Forms will be filled out for any serious and unexpected adverse events (see appendix 9). In the light of the information obtained, the association between the event and any of the study drugs will be judged as per the WHO guidelines (WHO, 1999). All serious and adverse events will be reported to the Data Safety and Monitoring Board (DSMB) within 48 hours they have been detected, and this will be followed up with a written report which will fully document the event; any available laboratory information or clinical data, will be included in the report.

**5.21 Participants withdrawal**

Parents will be at liberty to withdraw their children from participating in the study at any time for any reason without prejudice. Parents and legal guardians will be informed that, on medical grounds, the principal investigator can decide to withdraw their ward. Reason(s) for withdrawal will be written in the participant’s record form. In this case individuals will continue to receive all advantages to be enjoyed by continuing participants.

**5.22 Operational definitions**

1. Anaemia – Haemoglobin concentration of less than 11.0g/dl.
2. Severe anaemia – Haemoglobin concentration of less than 5.0g/dl.
3. Moderate anaemia – Haemoglobin concentration less than 8.0g/dl but greater than 5.0g/dl.
4. Parasitaemia – The presence of asexual blood stage parasites of any *Plasmodium* species detectable in a thick blood smear.
5. Clinical malaria- history of fever within the past 2 days or axillary temperature >37.5ºC + presence of asexual parasitaemia of any density + absence of any other obvious causes.
6. Severe malaria - presence of asexual parasitaemia + repeated generalized convulsions or impaired consciousness, hyperpyrexia (axillary temperature >39.5°C) + Hb<5.0g/dl.

**6.0 DATA MANAGEMENT AND ANALYSIS**

**6.1 Data Collection**

Data from participants will be recorded on specified forms (see appendix 3), which will be controlled in the field by the supervisors and their assistants for consistency and accuracy of the information. All documents will be transferred to Hohoe where other verifications will be performed by the principal investigator and the data manager.

6.2 Data entry and statistical analysis

All data will be entered twice into a database using EPI Info software. The accuracy of data input will be checked and validated using a customized validation programme.

The cleaned database will be converted into a Stata version 8 file (Stata Corporation, Texas, USA) prior to analysis. Analysis will be carried out on an intention-to-treat basis.

6.3 Measurement of study end-points

A trial profile will be produced, documenting the number of informed consent forms obtained, the numbers of children randomised to receive IPTc and who completed the follow-up, and the proportion of children who received the first, second, third, fourth, fifth and sixth interventions. Then baseline characteristics at inclusion among the four arms will be compared. Variables which will be included in this analysis are sex, age at inclusion, Z-score for weight for age (using EPINUT tables version 6) and use of mosquito nets at any time before the survey

Differences in proportions will be analysed using analysis of variance or multiple regression models where appropriate. The statistical significance of differences in mean Hb will be assessed using t-test. The rates of incidence of severe anaemia in the four study arms will be compared. The comparison of the incidence of malaria, severe malaria and severe anaemia will be analysed using Poisson regression models. Geometric means will be used for parasite densities.

The proportion of children withdrawn from the study as a result of adverse events will be determined, and the mean duration of time to onset of reported events will be determined. Differences in proportions of occurrences of specific adverse events will be analysed using analysis of variance or multiple regression models.

About 14% of the population in the district own bed nets; it is possible that this is a self-selected group. Though there might be low statistical power, it will afford the opportunity to explore the combined effect of ITN and IPTc in the subset of children who use ITNs.

The effect of the intervention on anthropometric indices will be assessed by comparing the proportions of children in each group with a weight for age z-score < -2 and the proportion of children with a height for age z-score <-2. Z-score differences between the pre and post intervention measurements will be calculated by groups. Mean differences of body mass index before and after the trial will be worked out and compared between the groups using paired t- test.

**7.0 ANCILLARY STUDY ONE: THERAPEUTIC EFFICACY OF STUDY DRUGS**

**7.1 Rationale**

## The rationale of this study is to determine the sensitivity of parasites in the study area to the drugs being used for IPTc (AQ+AS and SP) so that the results of the IPTc study can be interpreted correctly.

**7.2 Objectives**

1. To determine the proportion of children with early treatment failure (ETF) in the two treatment arms.
2. To determine the proportion of children with late treatment failure (LTF) in the two treatment arms.
3. To determine the proportion of children with adequate clinical and parasitological response (ACPR) in the two treatment arms.

**7.3 Study site and characteristics**

The study will be carried out in the district hospital and selected health centres with laboratory facilities and conducted by well trained laboratory personnel who have been involved in national drug efficacy trial for the past four years.

**7.4 Study design and methods**

**7.4.1 Study design**

This study will be an open, randomized clinical trial with two arms comparing the therapeutic efficacy of SP with a combination of AQ+AS to treat uncomplicated *P. falciparum* malaria. The key steps and arms of the study are shown in Figure 5.

**7.4.2 Study population**

The study population will be children aged between 3-59 months who do not belong to the IPTc trial in the district. The children who are sick and seek treatment at the selected health facilities will be recruited and followed up on days 3, 7, 14, 21, and 28 after treatment.

**Figure 6:** Design for drug efficacy

**Contact 0**: Assess for eligibility and random allocation to one of the two study groups and administration of the first course of treatment.

**Day 0**

AS+AQ Group

SP Group

**Days 1-2**

**Contacts 1and 2**:

1. Clinical assessment;

2. Drug administration.

**Contacts 3, 5 & 7**: 1. Clinical assessment;

2. Collect blood samples for Hb, blood film and filter paper.

**Days 3, 14 and 28**

**Contacts 4 & 6**: 1. Clinical assessment;

2. Collect blood samples for blood film and filter paper.

**Days 7 & 21**

7.5 Inclusion criteria

- Children between the ages of 3-59 months who report at the selected health facilities with malaria;
- Children who do not belong to the IPTc trial;
- Mono infection with *P falciparum,* with a parasitaemia in the range of 2000 to 100,000 asexual parasites per µl;
- Axillary temperature of ≥37.5 °C and < 40°C at the time of visit;;
- Absence of a cause of fever other than malaria;
- Ability to come for the stipulated follow-up visit, and resident with easy access to the health facility;
- Absence of history of hypersensitivity reactions to sulfonamide or any other drug;
- Absence of skin conditions which could increase the risk of severe adverse reaction to the scheduled drugs e.g. eczema and pemphigoid exanthemas;
- Consent by parent /guardian of child;
- Absence of disease conditions (e.g severe anaemia Hb<6.0g/dl, chronic diarrhoea and malnutrition) that could complicate interpretation of the results of the study;
- No sign of severe malaria;

Any child who fails to meet the above criteria will be excluded.

**7.6 Sample size**

The sample size was calculated based on the following assumptions:

- The expected cure rate of 85% by day 28 using SP and we expect the farthest from this rate to be ± 5%;
- The cure rate of AQ+AS in the study area is 94% by day 28 and the farthest from this rate will be ± 5.

At 95%CI using EPI Info, 203 children would be needed in each group.

# Assuming that at day 28 follow up rate will be only 90% of those that start, a total of

# 223 children (196/0.90=218) will be recruited for each group.

For this study, two hundred and eighteen 223 children 3-59 months will be required for each treatment regimen. This number will be adequate to observe the proportion of clinical failures which is unacceptable according to the thresholds defined above for a decision of change to be made.

**7.7 Randomisation**

Enrolled children will be assigned to treatment groups using restricted randomisation. The randomisation will be computer-generated in small block sizes of four to allocate children to the two treatment regimen. A sealed envelop method will be used for treatment allocation. This will be done by an investigator who is not directly involved with the patients. All staff involved in the recruitment and follow-up of the patients and parents will be aware of the study drug allocation. One of the treatment regimen (AQ+AS) which will be pre packaged blister tablets will be given, once a day, every day over three days. Pre-packed SP blister tablets will be given as a start dose. The two treatment regimen will be available at each of the study health centre that children in the trial might attend.

**7.8 Methods**

**7.8.1 Recruitment**

Once a child is eligible, information will be provided to the parents in the local languages about the study. Consent of the mother or guardian will then be sought and the child will assigned to a group. A mark and the group number will be written on the road to health card to facilitate identification at every contact in the health facilities or in the community during defaulter tracing.

7.8.2 Screening and enrollment

Screening for eligibility and enrolment into the study will be carried out the first day children with uncomplicated malaria visit the health facility. Screening will include measurement of temperature, examination of the child to rule out any other cause of fever, weight, collection of blood samples for Hb and blood film and filter paper for genetic markers of drug resistant parasites. The study objectives and procedures will be explained to caretakers of the children who are eligible and informed consent obtained from them. After obtaining written informed consent (see appendix 1b), there will be completion of an enrolment form which will contain information as in the main study.

**7.8.3 Drug administration**

The drugs will be crushed and mixed with water at the time of administration.

A proportion of children will vomit and / or spit out given drugs. All patients will be observed for at least fifteen minutes after drug administration. If vomiting occurs the patient will wait for 30 minutes and treatment will be given again. If vomiting occurs on a second occasion, parenteral therapy will be given and the child withdrawn from the trial.

**7.8.3.1 Treatment dosage**

For uncomplicated clinical malaria and according to the randomisation group, the treatment will be given at the following doses:

**SP:** 25mg/kg body weight sulfadoxine and 1.25 mg/kg pyrimethamine, as a single dose;

**AS:** 4 mg/kg body weight daily over three days;

**AQ:** 10 mg/kg body weight for the first 2 days, followed by 5 mg/kg the third day.

7.8.4 Follow ups of study children

When the study children return after recruitment on Day 1, the following procedures will be carried out: (1) clinical assessment of the child; (2) measurement of axillary temperature; (3) administration of the second dose of the study drug; (4) completion of the record form; (5) advice given to the caretakers to return their children to the health facility immediately if they notice that the child’s condition is getting worse or if something unusual happens to the child after drug administration; (6) appointment given for the next visit.

Meeting with the child at the health facility the next day (Day 2) the following procedures to be carried are similar to day1.

When the children return for the next meeting the following day(Day 3) the following procedures will be carried out:(1) clinical assessment of the child; (2) measurement of axillary temperature; (3) collection of blood samples for blood film and filter paper for genetic markers of drug resistance parasites;. (4) completion of the record form; (5) advice given to the caretakers to return their children to the health facility immediately when they notice that the child’s condition is getting worse than before or something unusual is happening to the child; (6) appointment given for the next visit in four days.

Meeting with the child at the health facility after day 3 (Day 7) the following procedures will be carried out: (1) clinical assessment; (2) measurement of axillary temperature; (3) collection of blood samples for blood film and filter paper for genetic markers of drug resistance parasites;(4) completion of the record form; (5) advice given to the caretakers to return their children to the health facility if the child is not well; (6) appointment given for the next visit in one week.

Meeting with the child at the health facility on the day of appointment (Day 14) the following procedures will be carried out: (1) clinical assessment; (2) measurement of axillary temperature; collection of blood samples for Hb, blood film and filter paper for genetic markers of drug resistance parasites; (4) completion of the record form; (5) advice given to the caretakers to return their children to the health facility if the child is not well; (6) appointment given for the next visit in one week.

Meeting with the child at the health facility on the appointed day (Day 21) the following procedures will be carried out: (1) clinical assessment; (2) measurement axillary temperature; (3) collection of blood samples for blood film and filter paper for genetic markers of drug resistance parasites; (4) completion of the record form; (5) advice given to the caretakers to return their children to the health facility if the child is not well; (6) appointment given for the next visit in one week.

Meeting with the child at the health facility on the appointed day (Day 28) the following procedures will be carried out: (1) clinical assessment; (2) measurement of axillary temperature; (3) collection of blood samples for Hb, blood film and filter paper for genetic markers of drug resistance parasites; (4) completion of the record form; (5) Explain to the parent or guardian about the performance of the child in the study; (6) give advice on malaria prevention and the use of ITNs advice; (7) Explain to her that incase the child gets malaria again within the period of the study the child can still be recruited; (8) child discharged from the study.

7.8.4.1 Tracing of defaulters

Any study child who is not presented for review on the appointed day will be traced to the house by the laboratory assistant and the study nurse that same day or the following day for blood sample taking and examination.

- - 1. **Alternative treatment of drug failure**

Children who will fail to respond to AQ+AS or SP will be given oral quinine in a dose of 10mg/Kg/body weight daily for 7 days.

- - 1. **Drop-outs from the study**

Any child who is lost to follow-up despite fulfilling all inclusion criteria and without developing exclusion criteria during the follow-up period will be considered as a dropout from the study.

**7.8.7 Exclusions from the study**

The following conditions will be classified as exclusions:

- Occurrence, during follow-up, of concomitant disease that would interfere with the clear classification of treatment outcome;
- Movement of patient from the study site to a place outside the reach of active follow up

(the movement must be unrelated to the response to treatment);

- Failure to complete treatment due to withdrawal of consent;
- Anti-malarial treatment administered by a third party during the follow up period;
- Detection during the follow up, of a mixed malaria infection.

A laboratory technician from the NMIMR will monitor quality of the slides and Hb readings through out the study period.

**Table 8:** Schedule for assessing the therapeutic efficacy of AQ+AS and SP alone

| Day | 0 | 1 | 2 | 3 | 7 | 14 | 21 | 28 |
| --- | --- | --- | --- | --- | --- | --- | --- | --- |
| History | X |  |  |  |  |  |  |  |
| Drug administration | X | X | X |  |  |  |  |  |
| Examination | X | X | X | X | X | X | X | X |
| Temperature | X | X | X | X | X | X | X | X |
| Blood Film | X |  |  | X | X | X | X | X |
| Filter Paper | X |  |  |  | X | X | X | X |
| Haemoglobin | X |  |  |  |  | X |  | X |

**7.8.8 Blood slides and haemoglobin**

Finger prick blood will be used for the measurement of Hb and to prepare two blood smears for every child. The prepared slides will be examined and Hb will be measured using the same technique described in the main study.

**7.8.9 Participants records**

On the case record form, name, age, weight and sex of the recruited child will be recorded. The following information on the mother or care-taker will also be recorded: name, age, occupation, educational background and name of community, full address including house number and landmark. These will later be transferred into the register.

The following information about the child will also be entered into the case record form and the register in the consulting room during follow up visits:

- Date of visit;
- Drug administration;
- Axillary temperature;
- Weight;
- Hb;
- Blood film for malaria parasites;
- Filter paper for PCR;
- Drug retention;
- Any adverse effect and type.

**7.8.10 End-points**

***Primary end-point***

The primary study endpoints will be the prevalence of *P. falciparum* asexual stage parasitaemia on days 3, 7, 14,21 and 28 after treatment.

Secondary end-points

1. Proportion of children with ACPR;
2. Proportion of children with ETF and LTF;

**7.8.10.1 Definition of trial end points**

The therapeutic response will be classified as ETF, if the patient develops one of the following conditions during the first three days of follow up:

- Development of danger signs or severe malaria on Day 1, Day 2, or Day 3, in the presence of parasitaemia;
- Axillary temperature ≥37.5°C on day 2 with parasitaemia> that of day 0 count;
- Axillary temperature ≥37.5°C on day 3 in the presence of parasitaemia;
- Parasitaemia on Day 3≥ 25% of count on day 0.

The therapeutic response will be classified as LTF, if the patient develops one of the following conditions during the follow-up period from Day 4 to Day 28:

- Development of danger signs or severe malaria in the presence of parasitaemia on any day from Day 4 to Day 28, without previously meeting any of the criteria of early treatment failure;
- Axillary temperature ≥37.5°C in the presence of parasitaemia on any day from Day 4 to Day 28, without previously meeting any of the criteria of early treatment failure.

The response to treatment will be classified as an ACPR, if the patient meets one of the following criteria:

- Axillary temperature <37.5°C and absence of parasitaemia from Day 7 to day 28, without previously meeting any of the criteria of early or late treatment failure;

PCR analysis of the filter paper samples will be used to partially resolve the problem of whether persistent parasitaemia is due to a new infection or recrudescence.

7.9 Data entry and statistical analysis

All data will be entered twice into a database using SPSS software. The accuracy of data input will be checked and validated using a customized validation programme. The cleaned database will be converted into a Stata version 8 file (Stata Corporation, Texas, USA) prior to analysis.

The baseline characteristics at inclusion among the three arms will be compared. Proportions of ETF, LTF and ACR and occurrence of adverse events in the two study arms will be compared. Differences in proportions will be analysed using analysis of variance or multiple regression models. Geometric means will be used for parasite densities. The proportion of children withdrawn from the study as a result of adverse events will be determined, and the mean duration of time to onset of reported events will be determined.

**8.0 ANCILLARY STUDY TWO: INVESTIGATION FOR REBOUND EFFECT OF IPTc**

- 1. **Rationale**

Several studies have raised the possibility of increased susceptibility to malaria and anaemia in individuals who stop chemoprophylaxis, whereas others have found no such association. The purpose of this study is to assess the possibility of a rebound effect in malaria morbidity after IPTc in a region with long intense malaria transmission season.

- 1. **Hypothesis**

IPTc using AQ+AS given every month or every two months or SP alone given every two months does not interfere with the development of a protective immune response to malaria.

**8.3 Objectives**

1. To determine whether IPTc in children under five years of age given for one year leads to a rebound effect in the incidence of severe malaria or severe anaemia;
2. To determine the incidence of clinical malaria among children in the four treatment arms after one year of IPTc;
3. To determine the prevalence of anaemia, moderate and severe anaemia in the different treatment groups after one year of IPTc.

**8.4 Study design and methods**

**8.4.1 Study area**

The study area will still be Hohoe district and the methods of this study will be the same as those employed for the main study except that drug administration will not be done. All children enrolled in the study will be actively followed for six months in the high transmission season. The key steps are shown in figure 6.

# 8.4.2 Study population

This will comprise all children who took part in the IPTc trial in the study area in 2005. A total of 2460 children aged between 3 to 59 months who still resident in the study area and are willing to participate will be included. It is anticipated that a minimum of 2260 (90%) of these children will be available for evaluation in 2006; they will be aged between 15 to 71 months.

All children remaining in the trial will be visited again prior to the high transmission season.

**Figure 7: Design of ancillary study 2**

**Contact 0**: Assessment for eligibility according to study groups in the main study and Cross-sectional survey (Collect blood samples for Hb, blood film and filter paper).

**Month 0 (M0)**

**(April 2006))**

Group GY:

SP every 2 months x 3 and placebo every 2months x3

Group GX As+AQ every 2 months x3 and placebo every 2 months x3

Group GW

AS+ AQ every month x6

Group GZ:

Placebo every month x 6

**Contacts 1-6**:

Passive surveillance at health facilities for clinical malaria, incidence of severe malaria and anaemia.

**M1-M6 (May-Oct. 2006)**

**Contact 7:**

Cross-sectional survey.

**M7 (Nov. 2006)**

**8.5 Entry criterion**

- Participated in the IPTc trial
- Still resident in the in the study area
- Willing to participate.

**8.6 Cross-sectional surveys**

Two cross-sectional surveys will be carried out and the same indicators will be measured as was done for the main study. In this study however, there will be measurements of axillary temperature and finger prick blood samples will be taken for Hb and blood film in both surveys.

**8.7 Study procedures**

Malaria surveillance will be carried out using the same passive surveillance methods as in the initial trial. Field workers and coordinators will visit the participants once a week from May to October. At the same time, parents and guardians will be strongly advised to come directly to the health centres if their child develops any symptoms or signs of illness between the weekly home visits. Children who have features of malaria will be referred to the nearest health centres for examination and appropriate treatment using the national guideline for treating malaria.

**8.8 Clinical management of study patients**

Passive surveillance with laboratory support will be continued by the medical assistants and nurses based in the same selected health facilities in the district following the same procedures as in the main study.

**8.9 End-points**

***Primary end-point***

The primary endpoints will be the incidence rate of clinical malaria during the high transmission season (May to October) in the IPTc randomisation groups.

***Secondary end-points***

1. The incidence rate of severe malaria and severe anaemia throughout the follow up period and will be compared between the four study arms;
2. prevalence of Hb≤8.0g/dl level at the before and after the high transmission season will be compared between the groups;
3. Prevalence of parasitaemia before and after the high transmission season will be compared between the groups;
4. Prevalence of anaemia, moderate and severe anaemia before and after the high transmission season will be compared between the groups.

**8.10 Data analysis**

End-points will be assessed using the same statistical tests as in the main study.

**8.11 Participants Records**

For each study child a case record form will be completed with all information as for the main study.

**8.12 Sample size for follow up for rebound effect**

**Table 9:** Sample size for the risk of clinical malaria in the second year following IPTc

|  | **Sample size per arm needed for a study with 80% power and 95% significance** | | | |
| --- | --- | --- | --- | --- |
| **Study arm** | Placebo group | SP group | AQ+AS every two months groups | AQ+AS every month group |
| Expected rate/prevalence | 0.50/child/yr | 0.55/child/yr | 0.60/child/yr | 0.70/child/yr |
| Relative risk of: |  |  |  |  |
| 0.8 | 408 | **342** | 287 | 200 |
| 0.85 | 720 | 600 | 500 | 343 |
| 0.9 | 1605 | 1331 | 1101 | 742 |

The number of children per arm needed for a study with 80% power and 95% significance for the primary outcome measure in the high transmission season during follow up for rebound effect is shown in table 9. The require sample size was calculated using EPI Info version 6 and based on the following assumptions:

1. The incidence of malaria in the second year following IPTc would be similar to the previous year in the placebo group 25% of these infection will be severe;

2. Attack rate of 0.5/child/year in the placebo group;

3. Attack rate 0.55 child/year in the SP;

4. Attack rate 0.6 child/year in the AQ+AS every two months;

5. Attack rate of 0.70 child/year in the AQ+AS every month group.

Allowing for 10% loss to follow-up 376 children per arm will have 80% power to detect at least 25% increase in the incidence of malaria in children who previously received IPT (RR=0.8)

The number of children needed for the follow-up for rebound effect study with 80% power and 95% significance for the primary would be 1504. However, all children remaining in the study will be included.

**9.0 TRIAL MANAGEMENT**

**9.1 Responsibilities of the Principal Investigator**

The principal investigator is responsible for:

- Elaboration of the protocol and the standard operating procedures (SOPs);
- Ensuring that parents are fully informed of the potential risks and benefits of - participation in the study;
- Recruiting and training the field staff;
- Ensuring that all administrative requirements are obtained;
- Ensuring global supervision of the data collection and data management;
- Reporting adverse events;
- Performing the analyses and final reports.

**9.2 Data Safety and Monitoring Board (DSMB) and interim analysis of data**

The trial will be conducted under conditions of good clinical practice (GCP), following as closely as possible the International Conference on Harmonisation (ICH) guidelines. SOPs will be developed for all major operations. A DSMB will be established to look after the trial. Members will include an experienced clinician, a statistician and a person with local knowledge of the study area. Discussions will be held with the DSMB prior to the start of the trial to identify their requirements for adverse events reporting and these will be met. The DSMB will also be consulted about the advisability of an interim analysis, in particular for the SP group as this drug may prove to be ineffective. The DSMB will be asked to review the analysis plan.

**9.3 Protocol amendments**

After the protocol has been finalised, changes can only be permitted with the agreement of the supervisor or the supervisory committee. Any changes will be formally endorsed and attached to the original protocol.

**9.4 Compensation of study subjects**

Compensation to study subjects who will sustain injuries as a direct result of their participation in this trial will be covered by the London School of Hygiene and Tropical Medicine insurance scheme.

**9.5 Local safety monitor**

A local physician will be assigned to be the local safety monitor (LSM) at the study site. He or she will have access to the codes of the study drugs. All serious adverse events will be reported to the LSM by the project physician. In the event of a serious adverse event associated with a study drug, the local safety monitor will be empowered to break the code for that child and, if it is considered necessary to temporarily suspend the trial, prior to discussion with the DSMB.

**9.6 Time Table of activities**

**Table 10. 0**

After the protocol development in London, the SOP manual will be elaborated in Ghana in close collaboration with local partners. (health authorities, malaria national control programme, NMIMR, OCRC at Hohoe, colleagues and villagers particularly).

| **Activities** | **Duration** | **Start** | **2004** | | **2005** | | | | **2006** | | | | **2007** | | | | **2008** | | | |
| --- | --- | --- | --- | --- | --- | --- | --- | --- | --- | --- | --- | --- | --- | --- | --- | --- | --- | --- | --- | --- |
|  |  |  | Q3 | Q4 | Q1 | Q2 | Q3 | Q4 | Q1 | Q2 | Q3 | Q4 | Q1 | Q2 | Q3 | Q4 | Q1 | Q2 | Q3 | Q4 |
| Protocol development | 6 months | Nov. 04 |  |  |  |  |  |  |  |  |  |  |  |  |  |  |  |  |  |  |
| SOP/Field work (RCT) | 8 months | April 05 |  |  |  |  |  |  |  |  |  |  |  |  |  |  |  |  |  |  |
| Passive surveillance | 18 months | May 05 |  |  |  |  |  |  |  |  |  |  |  |  |  |  |  |  |  |  |
| SOP/Field work (Drug efficacy study) | 18 months | May 05 |  |  |  |  |  |  |  |  |  |  |  |  |  |  |  |  |  |  |
| Data management | 18 months | May 05 |  |  |  |  |  |  |  |  |  |  |  |  |  |  |  |  |  |  |
| Preliminary analyses | 1 month | Dec. 05 |  |  |  |  |  |  |  |  |  |  |  |  |  |  |  |  |  |  |
| Upgrading seminar | 1 month | Jan. 06 |  |  |  |  |  |  |  |  |  |  |  |  |  |  |  |  |  |  |
| SOP/Field work (Rebound) | 8 months | April 06 |  |  |  |  |  |  |  |  |  |  |  |  |  |  |  |  |  |  |
| Biochemical analyses | 3 months | Feb. 06 |  |  |  |  |  |  |  |  |  |  |  |  |  |  |  |  |  |  |
| Breaking the  code | 1 month | Dec. 06 |  |  |  |  |  |  |  |  |  |  |  |  |  |  |  |  |  |  |
| Final analysis and editing | 7 months | Jan. 07 |  |  |  |  |  |  |  |  |  |  |  |  |  |  |  |  |  |  |
| Final thesis | 9 months | Aug. 07 |  |  |  |  |  |  |  |  |  |  |  |  |  |  |  |  |  |  |

* Q1, Q2, Q3 and Q4- first, second, third and fourth quarters of the year.

**10.0 Limitations of the study**

The impact of the intermittent treatment on anaemia might be affected by the fact that iron deficiencies, intestinal helminthes infection, HIV, sickle cell disease, malnutrition make significant contributions to the pathogenesis of anaemia in many African countries. However, there is evidence to suggest that in areas endemic for malaria, malaria is a major underlying factor (WHO, 2002).

The fact that some children will receive placebo may tempt some parents or caretakers to self-medicate their wards. The effect of the above mentioned factors will be minimized through:

1. Randomization;
2. Double blinding;
3. Provision of free medical care to all participants through out the study period.

**11.0 Dissemination of results**

Findings from this study will be presented at local and international conferences as well as to the chiefs, elders and people of the district. Reports will be submitted to the National Health Research Unit of the Ghana Health Service and to the Gates Malaria Partnership. Papers on the study findings will also be submitted for publication in peer reviewed journals.

**12.0 Ethical issues**

Written approval will be obtained from ethical committee of the Ghana Health Service/Ministry of Health (GHS/MoH) and the London School of Hygiene and Tropical Medicine before the commencement of the study. The study will be conducted in accordance with accepted principles on Ethics in Human Experimentation and ICH/GCP. Before inclusion into the study a written individual informed consent will be obtained from each mother or guardian of the child using a standard consent form. (See appendix 1).

Mother or guardian of the child will be informed about the duration and purpose of the study. They will also be informed that they are free to withdraw from the study any time, and this will not affect their subsequent treatment at the hospital or health centre.

**12.1 Discussion of ethical aspects of the proposal**

Giving placebo to children in one arm of the study is needed for the following reasons: (1) a placebo arm is essential for comparing the differences between groups; (2) to determine the optimum number of dose in one high transmission season. Study children will be monitored closely and if they develop clinical malaria they will be identified and treated promptly.

Giving SP to one arm of the study, even though we anticipate that there will be some level of SP resistance in the study area is justified on the grounds that (1) some children in this arm will benefit from IPTc; (2) as these children are asymptomatic, there is not imminent danger of progression to clinical malaria. Study children will be monitored closely and if they develop clinical malaria they will be identified and treated promptly.

Giving AQ+AS every month to one of the study arms is justified as this is the most suitable alternative treatment regimen to SP which has a long duration of action. AQ+AS every month needs to be used with caution in children since this might lead to a rebound effect.

However, side effects with these combinations are rare events and may be balanced by the ability of IPTc with AQ+AS every month to reduce the burden of malaria in children in areas with SP resistance.

Giving AQ+AS every two months to one of the study arms is justified as this is the most ideal strategy since this might not lead to a rebound effect and will not be too demanding and very expensive to integrate into routine programmes.

Collecting blood samples causes minor discomfort. However, the results of this blood examination will be used to offer appropriate treatment. The project has earmarked a budget for drugs for treatment of study children.

Prior to the start of the study the nature of the trial will be discussed with the study population at a series of village meetings. Details of the trial will be discussed with health and civil authorities in the study area. The benefits and risks of taking part in the study will be explained to caretakers at an individual level and their written consent will be obtained before enrolling children into the study. Approval for the study will be obtained from the ethics committees of the Ghana Health Service (GHS) and the LSHTM.

**Refferences**

Adjuik M, Babiker A, Borrman S, Brasseur P, Cisse M, Diallo S, Faucher JF, Garner P, Gikunda S, Kremsner PG, Krishna S, Lell B, Loolpapit M, Matsiegui P-B, Missinou MA, Mwanza J, Ntoumi F, Olliaro P, Osimbo P, Rezbach P, Some E, Taylor WR. Amodiaquine-artesunate versus amodiaquine for uncomplicated, Plasmodium falciparum malaria in African children: a randomised, multicentre trial. Lancet. 2002; 359:1365-1372.

Allen SJ, Snow RW, Menon A, Greenwood BM. Compliance with malaria chemoprophylaxis over a five year period among children in rural area of the Gambia. J Trop Med Hyg. 1990; 93: 313-322.

Allin MH, Ashton M, Kihamia CM, Mtey GJ, Bjorkmam A. Clinical efficacy and pharmacokinetics of artemisinin monotherapy and in combination with mefloquine in patients with falciparum malaria. British Medical Journal of Pharmaclogy. 1996; 41:587-592.

Alonso PL, Lindsay SW, Armstrong JRM, Conteh M, Hill AG, David PH, Fegan G, de Francisco A, Hall AJ, Shenton FC, Cham K and Greenwood BM. The effect of insecticide treated bed nets on mortality of Gambian children. Lancet. 1991;.337: 1499-1502.

Amukoye E, Winstanley PA, Watkins W.M, Snow RW, Hatcher J, Mosobo M, Ngumbao E, Lowe B, Ton M, Minyiri G, Marsh K. Chlorproguanil-dapsone: effective treatment for uncomplicated falciparum malaria. Antimicrobial Agents and Chemotherapy. 1997; 41(10):2261-2264.

Appawu M, Owusu-Agyei S, Dadzie S, Asoala V, Anto F, Koram K, Rogers W, Nkrumah F, Hoffman SL, Fryauff DJ. Malaria transmission dynamics at a site in northern Ghana proposed for testing malaria vaccines. Trop Med Int Health. 2004; 9(1):164-70.

Barber MA, Rice JB, Brown JY. Malaria studies on the firestone rubber plantation in Liberia, West Africa. Am J Hyg. 1932; 15: 601-623.

Basco, L.K., Eldin de Pecoulas, P., Wilson, C.M.W., Le Bras, J., Mazabraud, A. Point mutations in the dihydrofolate reductase-thymidylate synthetase gene and pyremethamine and cycloguanil resistance in Plasmodium falciparum. Mol. Biochem. Parasitol. 1995; 69:135-138

Basco LK, Same-Ekobo A, Ngane VF, Ndounga M, Metoh T, Ringwald P, Soula G. Therapeutic efficacy of sulfadoxine-pyremethamine, amodiaquine and the sulphadoxine-pyremethamine-amodiaquine combination against uncomplicated Plasmodium falciparum malaria in young children in Cameroon. Bulletin of the World Health Organisation. 2002; 80(7):538-545.

Beach RF, Ruebush TK 2nd, Sexton JD, Bright PL, Hightower AW, Breman JG, Mount DL, Oloo AJ. Effectiveness of permethrin-impregnated bednets nad curtains for malaria control in holoendemic area of western Kenya. American Journal of tropical medicine and hyg. 1993; 49(3): 290-300.

Binka FN, Kubage A, Adjuik M, Williams LA, Lengeler C, Maude GH, Armah GE, Kajihara B, Adiamah JH, Smith PG. Impact of permethrin impregnated bednets on child mortality in Kassena-Nankana District, Ghana: a randomized controlled trial. Trop Med Int Health. 1996; 1(2):147-154.

Binka FN, Morris SS, Ross DA, Arthur P, Aryeetey ME. Patterns of malaria morbidity and mortality in children in northern Ghana. Trans R Soc Trop Med Hyg. 1994; 88:381-385.

Black RH. Malaria in the Australian army in South Vietnam: successful use of a proguanil-dapsone combination for chemoprophylaxis of chloroquine-resistant falciparum malaria. Med J Aust. 1973; 1(26):1265-70.

Bloland PB, Lackritz EM, Kazembe PN, Were JBO, Steketee R, Campbell CC. Beyond chloroquine: implications of drug resistance for evaluating malaria therapy efficacy and treatment policy in Africa. J Infect Dis. 1993; 167:932-937.

Bloland PB, Ruebush TK. Amodiaquine. Lancet. . 1996. 348:1659-60.

Bradley-Moore A, Greenwood B, Bradley A, Akintunde A, Attai ED, Fleming AF, Flynn FV, Kirkwood BR, Gilles HM. Malaria chemoprophylaxis with chloroquine in young Nigerian children. Its effect on haemoglobin measurements. Annals of Tropical Medicine and parasitology. 1985; 29:585-595.

Brasseur P, Guiguemde R, Diallo S, Guiyedi V, Kombila M, Ringwald P, Olliaro P. Amodiaquine remains effective for treating uncomplicated malaria in West and Central Africa. Trans R Soc Trop Med. 1999; 93:645-650.

Browne ENL, Maude GH, Binka F.N. The impact of insecticide-treated bed nets on malaria and anaemia in pregnancy in Kassena-Nankana district: a randomized controlled trial. Trop Med Int Health. 2001; 6(9):667-676.

Bruce-Chwatt LJ, Hutchinson DB. Agranulocytosis associated with Maloprim. Br Med J (Clin Res Ed). 1984 ; 288(6410):65-6.

Chandramohan D, Jaffar S, Greenwood BM. Use of clinical algorithms for diagnosing malaria. Trop Med Int Health. 2002; 7:45-52.

Cisse B, Cheikh S. Sokhna, Neal Alexander, Jean Francois Trape, Brian Greewood. A double blind, randomised placebo-conrolled trial to measure the potential of sIPT with artesunate plus SP to reduce the malaria burden in Niakhar, rural Senegal Absract ASTMH 53rd Annual meeting. 2004.

Curtis J, Duraisingh MT, Warhurst DC. In vivo selection for a specific genotype of dihydropteroate synthetase of Plasmodium falciparum by pyremethamine-sulfadoxine but not chlorproguanil-dapsone treatment. J Infect Dis. 1998; 177:1429-1433.

D'Alessandro U, Langerock P, Bennett S, Francis N, Cham K, Greenwood BM. The impact of a national impregnated bed net programme on the outcome of pregnancy in primigravidae in The Gambia. Trans R Soc Trop Med Hyg. 1996; 90: 487-492.

D'Alessandro U, Olayele BO, McGuire W, Thomson MC, Langerock P, Bennett S, Greenwood BM. Mortality and morbidity from malaria in Gambiam children after introduction of impregnated bednet programme. Lancet. 1995; 345: 479-483.

Dicko A, Issaka Sagara, Mahamadou S, Sissoko, Ousmane Guindo, Abdou Baki I, Mamady Kone, Mahamadou A. Thera, Massambou Sacko, Ogobara K Doumbo.. Impact of intermittent preventive treatment with sulphadoxine-pyrimethamine targeting the transmission season in the incidence of clinical malaria in children of 6 months to 10 years in Kambila Mali. Absract ASTMH 53rd Annual meeting 2004.

Dorsey G, Njama D, Kamya MR, Cattamanchi A, Kyabayinze D, Staedke SG, Gasasira A, Rosenthal PJ. Sulfadoxine/pyremethamine alone or with amodiaquine or artesunate for treatment of uncomplicated malaria: a longitudinal randomized trial. Lancet. 2002; 360:2031-2038.

Edstein MD, Looareesuwan S, Wilairatana P, Vanijanonta S, Kyle DE, Rieckmann KH. Disposition of proguanil in Thai patients with uncomplicated falciparum malaria. Am J Trop Med Hyg. 1997; 56(5):498-502.

Fleming AF, Akintunde A, Attai ED, Bradley-Moore AM, Greenwood BM, Bradley AK, Kirkwood BR, Gilles HM. Malaria and haemoglobin genotype in young northern Nigerian children. Ann Trop Med Parasitol. 1985; 79(1):1-5.

Fleming AF, Ghatoura GB, Harrisson KA, Briggs ND, Dunn DT. The prevention of anaemia in pregnancy in primigravidae in the guinea savanna of Nigeria. Ann Trop Med Parasitol. 1986; 80(2):211-33.

Friman G, Nystrom-Rosander, C., Jonsell, G., Bjorkman, A., Lekas, G., Svendsrup, B. Agranulocytosis associated with malaria prophylaxis with maloprim. Br Med J. 1983; 286(6373):1244-1245.

Garfield RM, Vermund SH. Changes in malaria incidence after mass dru administration in Nicaragua. Lancet ii: 1983; 500-503.

Geerlings P, Brabin B, Eggelte T. Analysis of the effects of malaria chemoprophylaxis in children on haematological responses, morbidity and mortality. Bull Wld Hlth Org. 2003; 81: 205-216.

Giglioli G, Rutten FJ Ramjattan S. Interruption of malaria transmission by chloroquinised salt in Gyyana with observations on chloroquine-resistant strain of plasmodium falciparum. Bull World Health Organ. 1967; 36: 283-301.

Greenwood BM, David PH, Otoo-Forbes L, Allen SJ, Alonso PL, Armstrong-Schellenberg JR, Byass P, Hurwitz M, Menon A, Snow RW. Mortality and morbidity from malaria after stopping chemoprophylaxis. Trans R Soc Trop Med Hyg. 1995; 89: 629-633.

Greenwood BM, Greenwood AM, Bradley AK, Snow RW, Byass P, Hayes RJ, N'Jie ABH. Comparison of two strategies for control of malaria within primary health care programme in The Gambia. Lancet i: 1988; 1121-1127.

Greenwood BM, Greenwood AM, Snow RW, Byass P, Bennett S and Hatib-N'jie AB. The effects of malaria chemoprophylaxis given by traditional birth attendants on the course and outcome of pregnancy. Trans R Soc Trop Med Hyg. 1989; 83: 589-594.

Habluetzel A, Diallo DA, Esposito F, Lamizana L, Pagnoni F, Lengeler C, Traore C, Cousens SN. Do insecticide-treated curtains reduce all-cause child mortality in Burkina-Faso? Trop Med Int Health. 1997; 2(9):855-862.

Harrison G. Mosquitoes,Malaria and Man; A history of the Hostilities since 1880..John Murrray. 1978.

Heymann DL, Steketee RW, Wirima JJ, McFarland DA, Khoromana CO, Campbell CC. Antenatal chloroquine chemoprophylaxis in Malawi: chloroquine resistance, compliance, protective efficacy and cost.Trans R Soc Trop Med Hyg. 1990; 84(4):496-8.

Holtz TH, Marum LH, Mkandala C, Chizani N, Roberts JM, Macheso A, Parise ME, Kachur SP. Insecticide-treated bednet use, anaemia, and malaria parasitaemia in Blantyre District, Malawi. Trop Med Int Health. 2002; 7(3):220-30.

Hutchinson DB, Whiteman PD, Farquhar JA. Agranulocytosis associated with maloprim: review of cases. Hum Toxicol. 1986; 5(4):221-227.

Kaneko A, Taleo G, Kalkoa M Yamar S Kobayakawa T, Björkman A. Malaria eradication of islands. Lancet. 2000; 356: 1560-1564.

Kanyok T Clinical development of Lapdap for malaria. Presentation at a meeting of the American Society of Tropical Medicine and Hygiene, Atlanta, November 12^th^, 2001.

Keuter M, van Eijk A, Hoogstrate M, Maarten R , van de Ree M, Ngwawe WA, Watkins Were JBO, Brandling-Bennett AD. Comparison of chloroquine, pyremethamine and sulfadoxine, and clorproguanil and dapsone as treatment fro falciparum malaria in pregnant and non-pregnant women, Kakamega district, Kenya. Br Med J. 1990; 301:466-470.

Kitua A, Smith T, Alosno PL, Urassa H, Massanja H, Kimarion J, Tanner M. The role of low level plasmodium Falciparum parasitaemia in anaemia among infants living in an area of intense and perennial transmission. Trop Med Int Health. 1997; 2:325-333.

Koram KA, Owusu-Agyei S, Utz G, Binka F.N, Hoffman S L, Nkrumah FK. Severe anaemia in young children after high and low malaria transmission seasons in the Kassena-Nankana district of northern Ghana. Am J Trop Med Hyg. 2000; 62: 670-674.

Kurtzhals JAL, Rodrigues O, Addae M, Commey JOO, Nkrumah FK and Hviid L. Reversible suppression of bone marrow response to erythropoietin in Plasmodium falciparum malaria. British Journal of Hematology. 1997; 97: 169-174.

Laing AB. The impact of malaria chemoprophylaxis in Africa with special reference to Madagascar, Cameroon, and Senegal. Bull World Health Organ. 1984; 62 Suppl:41-8.

Lee PS, Lau EYL. Risk of acute non-specific upper respiratory tract infections in healthy men taking dapsone-pyremethamine for prophylaxis against malaria. British Medical Journal. 1988; 296:893-895.

Lozoff B, Jimenez E, Wolf AW. Long-term developmental outcome of infants with iron deficiency. New Engl J Med 1991; 325:687-94.

Luxemburger C, Price R, Nosten F, Ter Kuile F, Chongsuphajaisiddhi, White N. mefloquine in infants and young children. Annals of Tropical Paediatrics. 1996; 16: 281-286.

MacArthur JR, Stennies GM, Macheso A, Kolczak MS, Green MD, Ali D, Barat LM, Kazembe PN, Ruebush TK. Efficacy of mefloquine and sulphadoxine-pyrimethamine for the treatment of uncomplicated Plasmodium falciparum infection in the Machinga District, Malawi. 1998. Am J Trop Med Hyg 2001;65:679-684.

Marbiah NT, Petersen E, David K, Magbity E, Lines J& Bradley DJ. A controlled trial of lambda-cyhalothrin-impregnated bednets and/or dapson/pyrimethamine for malaria control in Seirra Leone. Am J of trop Med and Hyg. 1998; 58, 1-6.

Massaga J, Kitua A, Lemnge M et al. Intermittent treatment with amodiaquine markedly reduces the incidence of anaemia and malaria fevers during the first year of life: a randomised placebo controlled trial in an area highly endemic for drug resistant malaria. Abstract number 161. 3rd Pan African Conference on Malaria. 2002; Arusha, Tanzania.

Massaga JJ, Kitua AY, Lemnge MM, Akida JA, Malle LN, Rønn AM, Theander TG, Bygbjerg IC. Effect of intermittent treatment with amodiaquine on anaemia and malarial fevers in infants in Tanzania: a randomized placebo-controlled trial. Lancet. 2003; 361(9372):1853-1860.

Maxwell CA, Myamba J, Njunwa KJ, Greenwood B.M, Curtis CF. Comparison of bednets impregnated with different pyrethroids for their impact on mosquitoes and on re-infection with malaria after clearance of pre-existing infections with chlorproguanil-dapsone. Trans R Soc Trop Med Hyg. 1999; 93:4-11.

McGregor IA, Gilles HM, Walter JH, Davies AH, Pearson FA, Effects of heavy and repeated malaria infections on Gambian infants and children. Effects of erythrocytic parasitization. BMJ ii. 1956; 686-692.

McIntosh HM, Greenwood BM. Chloroquine or amodiaquine combined with sulfadoxine-pyremethamine as a treatment for uncomplicated malaria - a systematic review. Ann Trop Med Parasitol. 1998; 93(3): 265-270.

McIntosh HM. 2002. Chloroquine or amodiaquine combined with sulfadoxine-pyremethamine for treating uncomplicated malaria(Cochrane Review). In: The Cochrane Library, Issue 4. 2002. Oxford:Updated Software.

Menendez C, Kahigwa E, Hirt R, Vounatsou P, Aponte JJ, Font F, Acosta CJ, Schellwnberg DM, Galindo CM, Kimario J, Urassa, Brabin B, Smith TA, Kitua AY, Tanner M, Alonso PL. Randomised placebo controlled trial of iron supplementation and malaria chemoprophylaxis for prevention of severe anaemia and malaria in Tanzanian infants. Lancet. 1997; 350:844-850.

Menendez C, Todd J, Alonso PL, Lulat S, Francis N, Greenwood BM. Malaria chemoprophylaxis, infection of the placenta and birth weight in Gambian primigravidae. J Trop Med Hyg. 1994; 97(4): p. 244-8.

Meshnick SR. Artemisinin: mechanism of action, resisitance and toxicity. International Journal for Parasitology. 2002; 32:1655-1660.

Meuwissen JHET. The use of medicated salt in an antimalaria campaign in West New Guinea. Trop Geoegr Med. 1964; 16: 245-255.

Mockenhaupt FP. Mefloquine resistance in Plasmodium falciparum. Parasitol Today 1995; 11:248-253.

Mockenhaupt FP, Eggelte TA, Bohme T, Thompson WN, Bienzle U. Plasmodium falciparum dihydrofolate reductase alleles and pyremethamine use in pregnant Ghanaian women. Am J Trop Med Hyg. 2001; 65(1):21-26.

MolineauxL, Gramiccia G. The Garki project. Geneva: 1980; World Health Organization.

Moyou-Somo R, Lehman LG, Awahmukalah S& Ayuk Enyong P. Deltametrin impregnated bednets for the control of urban malaria in Kumba Town, South-West Province of Cameroon. Jounal of Tropical Medicine and Hygine. 1995; 98, 319-324.

Muller O, van Hensbroek MB, Jaffar S, Drakeley C, Okorie C, Joof D, Pinder M, Greenwood B. A randomized trial of chloroquine, amodiaquine and pyremethamine-sulphadoxine in Gambian children with uncomplicated malaria. Trop Med Int Health. 1996; 1(1): 124-132.

Murphy SC, Breman JG. Gaps in the childhood malaria burden in Africa: cerebral malaria, neurological sequelae, anaemia, respiratory distress, hypoglycaemia and complications of pregnancy. Am J Trop Med Hyg 2001; 64 (1-2 suppl) 57-67.

Mutabingwa TK, Eling WM, Kitinya JN, Malle LN, de Geus A. Malaria chemosuppression in pregnancy. V. Placenta malarial changes among three different prophylaxis groups.Trop Geogr Med. 1993; 45(6):274-9.

Mutabingwa T, Nzila A, Mberu E, Nduati E, Winstanley P, Hills E, Watkins W. Chlorproguanil-dapsone for treatment of drug-resistant falciparum malaria in Tanzania. Lancet. 2001; 358(9289):1218-1223.

Nahlen BL, Akintunde A, Alakija T, Nguyen-Dinh P, Ogunbode O, Edungbola LD, Adetoro O, Breman JG. Lack of efficacy of pyrimethamine prophylaxis in pregnant Nigerian women. Lancet. 1989; 2(8667):830-4.

Ndounga M, Basco LK. Rapid clearance of Plasmodium falciparum hyperparasitaemia after oral amodiaquine treatment in patients with uncomplicated malaria. Acta Tropic. 2003; 88(1):27-32.

Nevill CG, Some ES, Mung'ala VO, Mutemi W, New L, Marsh K, Lengeler C, Snow RW. Insecticide-treated bednets reduce mortality and severe morbidity from malaria among children on the Kenyan coast. Trop Med Int Health. 1996; 1(2):137-138.

Njugi IK, Chiguzo AN, Guyatt HL. A cost analysis of employer-based bednet programme in Coastal and Western Kenya. Health Policy and Planning. 2004; 19 (2): 111-119.

Nosten F, Brasseur P. Combination therapy for malaria. Drugs. 2002; 62(9):1315-1329.

Nzila AM, Nduarti E, Mberu EK, Hopkins-Sibley C, Monks SA, Winstanley PA, Watkins W.M. Molecular evidence of greater selective pressure from drug resistance exerted by the long-acting antifolate pyremethamine/sulfadoxine compared with the shorter-acting chloproguanil/dapsone on Kenyan Plasmodium falciparum. The Journal of Infectious Diseases. 2000; 181:2023-2028.

Olliaro P, Nevill C, LeBras J, Ringwald P, Mussano P, Garner P, Brasseur P. Systematic review of amodiaquine treatment in uncomplicated malaria. Lancet. 1996; 348:1196-1201.

Olliaro P, Mussano P. Amodiaquine for treating malaria (Cochrane Review). 2002; IN: The Cochrane Library, Issue 4. Oxford: Update Software.

Oppenheimer SJ. Iron and malaria. Parsitol Today. 1989; 5(3): 11-9.

Owusu A, Fryauff D, Chandramohan D Koram KA, Binka FN, Nkrumah Fk, Utz GC, Hoffman SL. Characteristics of severe anaemia and its association with malaria in young children of the Kassena-Nankana district of northern Ghana. Am J Trop Med Hyg. 2002; 67: 371-377.

Payne D. 1988. Did medicated salt hasten the spread of chloroquine resistance in plasmodium falciparum? Parasitol Today 4: 112-115.

Price RN, Nosten F, Luxemberger C, ter Kuile FO, Paiphun L, Chongsuphajaisiddhi T, White NJ. Effect of artemisinins derivatives on malaria transmissibility. Lancet. 1996; 347:1654-1658.

Prissen Geerlings PD, Brabin BJ, Eggelte TA. Analysis of the effect of malaria chemoprophylaxis in children on haematological response, morbidity and mortality. Bull World Health Organ. 2003; 81: 206-216.

Ringwald P, Keudjian A, Same-Ekobo A, Basco LK. Chemoresistance of Plasmodium falciparum in the urban areas of Yaoundé, Cameroon. Part 2: Evaluation of the efficacy of amodiaquine and sulfadoxine-pyremethamine combination in the treatment of uncomplicated Plasmodium falciparum malaria in Yaoundé, Cameroon. Trop Med Int Health. 2000; .5: 620-627.

Rogerson SJ, Chaluluka E, Kanjala M, Mkundika P, Mhango C, Molyneux ME. Intermittent sulfadoxine-pyrimethamine in pregnancy: effectiveness against malaria morbidity in Blantyre, Malawi, in 1997-99. Trans R Soc Trop Med Hyg. 2000; .94 (5): 549-53.

Schellenberg D, Kahigwa E, Drakeley C, Malende A, Wigayi J, Msokame C, Aponte JJ, Tanner M, Mashinda H, Menendez C, Alonso PL. The safety and efficacy of sulphadoxine-pyrimethamine, amodiaquine and their combination in the treatment of uncomplicated Plasmodium falciparum malaria. Amer J Trop Med Hyg. 2002 ; 67:17-23.

Schellenberg D, Menendez C, Kashigwa E, Aponte JJ, Vidal J, Tanner M , Mshinda H, Alonso P. Intermittent treatment for malaria and anaemia control at time of routine vaccination in Tanzanian infants; a randomised, placebo-controlled trial. Lancet. 2001; 357: 1471-1477.

Schultz LJ, Steketee RW, Macheso A, Kazembe P, Chitsulo L, Wirima JJ. The efficacy of antimalarial regimens containing sulfadoxine-pyremethamine and/or chloroquine in preventing peripheral and placental Plasmodium falciparum infection among pregnant women in Malawi. Am J Trop Med Hyg. 1994; 51(5):515-522.

Sexton JD, RuebushTK 2nd, Brandling-Bennet AD, Breman JG, Roberts JM, Odera JS, Were JB. Permethrine-impregnated curtains and bednets prevent malaria in western Kenya Ame j of Trop Med and Hyg. 1990; 43, 11-18.

Shanks GD, Edstein MD, Suriyamongkol V, Timsaad S, Webster HK. Malaria chemoprophylaxis using proguanil-dapsone combinations in the Thai-cambodian border. Am J Trop Med Hyg. 1992; 46(6):643-648.

Shulman CE, Dorman EK, Cutts F, Kawuondo K, Bulmer J.N, Peshu N, Marsh K. Intermittent sulphadoxine-pyremethmine to prevent severe anaemia secondary to malaria in pregnancy: a randomized placebo-controlled trial. Lancet. 1999; 353:632-636.

Shulman CE, Dorman EK, Talisuma AO, Lowe BS, Nevill C, Snow RW, Jilo H, Peshu N, Bulma JN, Graham S, Marsh K. A community randomized controlled trial of insecticide- treated bednets for the prevention of malaria and anaemia among primigravid women on the Kenyan Coast. Trop Med Int Health. 1998; 3:197-204.

Shultz LJ, Steketee RW, Macheso A, Kazembe P, Chitsulo L, Wirma JJ. The efficacy of anti-malarial regimens containing sulphadoxine-pyrimethamine and /or chloroquine in preventing peripheral and placental Plasmodium falciparum infection among pregnant women in Malawi. Am J Trop Med Hyg. 1994; 51: 515-522.

Smith A, Hendrickse R, Harrison C, Hayes R, Greenwood B. Iron deficiency anaemia and its response to oral iron: report of a study in rural Gambian children treated at home by their mothers. Ann Trop Peadiatr. 1989; 9:6-16.

Snow R, Omumbo J, Lowe B, Molyneux CS, Obiero JO, Palmer A, Weber MW, Pinder M, Nahlen B, Obonyo C, Newbold C, Gupta S, Marsh K. Relation between severe malaria morbidity in children and level of plasmodium falciparum transmission in Africa. Lancet. 1997; 349:1650-54.

Sowunmi A, Oduola AM. Open comparison of mefloquine, sulfadoxine/pyrimethamine, and chloroquine in acute uncomplicated falciparum malaria in children. Trans R Soc Trop Med Hyg. 1995; 89:303-305.

Sowunmi A, Ayede AI, Falade AG, Ndikum VN, Sowunmi CO, Adedeji AA, Falade CO, Happi TC, Oduola AM. Randomized comparison of chloroquine and amodiaquine in the treatment of acute uncomplicated Plasmodium falciparum malaria in children. Ann Trop Med Parasitol. 2001; 95(6):549-558.

Staedke SG, Kamya MR, Dorsey G, Gasasira A, Ndeezi G, Charlebois ED, Rosenthal PJ. Amodiaquine, sulfadoxine/pyrimethamine, and combination therapy for treatment of uncomplicated falciparum malaria in Kampala, Uganda: a randomised trial. Lancet. 2001; 358:368-374.

Steketee RW. Recent findings in perinatal malaria. Bull Int Pediatr Assoc. 1989; 10:418-433.

Steketee RW, Wirima JJ, Slutsker L, Khoromana CO, Heymann DL, Breman JG. Malaria treatment and prevention in pregnancy: indications for use and adverse events associated with use of chloroquine or mefloquine. Am J Trop Med Hyg. 1996; 55(1):50-56.

Sulo J, Chimpeni P, Hatcher J, Kublin JG, Plowe CV, Molyneux ME, Marsh K, Taylor TE, Watkins WM, Winstanley PA. Chloroproguanil-dapsone versus sulfadoxine-pyrimethamine for sequential episodes of uncomplicated falciparum malaria in Kenya and Malawi: a randomised clinical trial. Lancet. 2002; 360:1136-1143.

Targett G, Drakeley C, Jawara M, von Seidlein L, Coleman R, Deen J, Pinder M, Doherty T, Sutherland C, Walraven G, Milligan P. Artesunate reduces but does not prevent posttreatment transmission of Plasmodium falciparum to Anopheles gambiae. The Journal of Infectious Diseases. 2001; 183:1254-1259.

ter Kuile FO, Terlouw DJ, Phillips-Howard PA, Hawley WA, Friedman JF, Kolczak MS, Kariuki SK, Shi YP, Kwena AM, Vulule JM, Nahlen BL. Impact of permethrin-treated bed nets on malaria and all-cause morbidity in young children in an area of intense perennial malaria transmission in western Kenya: cross-sectional survey. Am J Trop Med Hyg. 2003; 68(4 Suppl):100-7.

Trans R Soc Trop Med Hyg. 84(4):496-8.

Trigg JK, Mbwana H, Chambo O, Hills E, Watkins W, Curtis C.F. Resistance to pyremethamine/sufadoxine in Plasmodium falciparum in 12 villages in north east Tanzania and a test of chlorproguanil/dapsone. Acta Tropica. 1997; 63:185-189.

Von Seidlein L, Walraven G, Milligan PLM, Alexander N, Manneh F, Deen JL, Coleman R, Jawara M, Lindsay SW, Drakeley C, De Martin S, Olliaro P, Bennett S, Schimm M, Okunoye K, Targett GAT, McAdam KPWJ,, Doherty JF, Greewood BM, Pinder M. The effect of mass drug administration of pyrimethamine/sulphadoxine combined with artesunate on malaria transmission: a double blind, community randomised, and placebo controlled trial in The Gambia. Trans R Soc Trop Med Hyg . 2003; 97: 217-225.

von Seidlein L, Milligan P, Pinder M, Bojang K, Anyalebechi C, Gosling R, Coleman R, Ude JI, Sadiq A, Duraisingh M, Warhurst D, Alloueche A, Targett G, MacAdam K, Greenwood B, Walraven G, Olliaro P, Doherty T. Efficacy of artesunate plus pyrimethamine-sulphadoxine for uncomplicated malaria in Gambian children: a double-blind, randomised, controlled trial. Lancet. 2000; 355(9201): p. 352-7.

Watkins WM, Mberu EK, Winstanley PA, Plowe C. The efficacy of antofolate combinations in Africa: a predictive model based on pharmacodynamics and pharmacokinetic analyses. Parasitology Today. 1997; 13:459-464.

Watkins WM, Sixsmith DG, Spencer HC, Boriga DA, Kariuku DM, Ripingor T, Koech DK. Effectiveness of amodiaquine as treatment for chloroquine resistant Plasmodium falciparum infections in Kenya. Lancet. 1984; 1(8373): 357-359.

White NJ. Delaying antimalarial drug resistance with combination chemotherapy. Parassitologia. 1999; 41: 301-308.

White NJ, Nosten F, Looareesuwan S. 1999. Averting a malaria disaster. Lancet. 353:1965-67.

White NJ. Can amodiaquine be resurrected? Lancet. 1996; 348:1184-1185.

Winstanley P. Chlorproguanil-dapsone (LAPDAP) for uncomplicated falciparum malaria. Trop Med Int Health. 2001, 6(11):952-4.

Winstanley PA, Ward SA, Snow RW. Clinical status and implications of antimalarial drug resistance: Microbes Infect. 2002; 4(2):157-64.

World Health Organization. African Summit on Roll Back Malaria, Abuja Nigeria,WHO, Geneva. 2000;WHO/CDS/RBM/2000.1.7.

World Health Organization.. Ad Hoc Committee on Health Research Relating Future Intervention Options. Investing in health research and development. Geneva: 1996;WHO, Document TDR/Gen/96.1.

World Health Organization. Meeting on goal of malaria treatment policy in the Africa Region. 2003; 14-15 August 2003, Harare, Zimbabwe.

World Health Organization. Expert Committee on Malaria, 18th Report. 1986; Technical Report Series No. 735.

World Health Organization. The use of antimalarial drugs. 2001; WHO/CDS/RBM/2001.33.

**Appendix 1a: Hohoe district intermittent preventive treatment in children study**

**INFORMATION SHEET AND CONSENT FORM**

In Ghana and in other parts of Africa, malaria and anaemia are important health problems in young children. Studies undertaken recently in Senegal and Mali have shown that these conditions can be partially prevented by giving children treatment with anti-malarial drugs on several occasions during the high transmission season, whether or not they are sick with malaria at that time. This way of trying to prevent some of the bad effects of malaria is called intermittent preventive treatment in children (IPTc).

The drugs that have been used so far for IPTc are sulphadoxine/pyrimethamine, usually called SP or Fansidar, and a combination of SP with artesunate (SP+AS). Unfortunately, the malaria parasite is becoming resistant to SP in Ghana and in many other parts of Africa. We need to find a safe and effective alternative to this drug for use in IPTc and we would like you to help us to do this by enrolling your child in a study which will be conducted by staff from the District health Directorate (DHD), district hospital and health centres within Hohoe district and the London School of Tropical Medicine. This trial, which has the approval of the authorities in Ghana, is being conducted with support from the Bill and Melinda Gates Foundation.

We want to compare the usefulness in IPTc of three drugs. These are;

1. SP. SP is the drug currently recommended for IPT and, even though the parasite is becoming resistant to it, SP may still provide some protection.
2. AQ+AS. This drug combination is currently recommended for treatment of malaria in Ghana and is registered for use in the country.

- The advantage of AQ is that it is still more effective than chloroquine (CQ). The standard doses of AQ used for treatment are generally similar to CQ. It can sometimes cause nausea, vomiting, abdominal pain, diarrhoea and itching. There is some evidence that itching may be less common with AQ than CQ;
- The advantage of AS is that it is one of the most potent and rapidly acting anti-malarial drug. There is evidence that use of artemisinins based combinations can greatly retard development of resistance to the partner drug.

If you agree that your child can take part in the trial, this is what will happen.

1. We will register your child and give him/her an ID card;
2. We will visit the community every month during the rainy season from April to October this year and he/she will be given a course of treatment with one of the anti-malarial drugs described above or with a placebo tablet. It is necessary that some children receive the placebo tablet so that we can tell if the anti-malarial tables are working properly or causing any side effects;
3. We will follow the progress of your child until s/he for eighteen months. Whenever your child is sick please bring him/her to the nearest health centre or hospital and show the staff the ID card;
4. In order to determine if the anti-malarial drugs are protecting your child against malaria and anaemia we will need to collect some small samples of blood by pricking the child’s finger on three occasions at an interval of six months to confirm that the treatment has worked;
5. Your child will be followed up next year to determine whether any of the treatment regimens will lead to an increase in malaria attacks in the year following IPTc. When your child is seen next year his/her blood will be examined for malaria parasites in April and in November and any child who is found to have malaria will be treated with one of the drugs mentioned above.

You are completely free to choose whether you would like your child to participate in this study or not and you are free to withdraw your child at any time. If you do not participate or withdraw your child he/she will receive all their usual vaccinations and treatment.

The information obtained on your child during the course of the study will be available only to the staff helping with the trial.

Do you have any questions about the study? If you want any more information at any time during the study please contact:

1. The principal investigator Dr Margaret Kweku at the district hospital;
2. The Doctor, Medical Assistant or the nurse of the hospital/health centre ;
3. The District Director of Health Services;
4. Coordinator in your community.

If you agree to participate, please sign or thump print the consent form.

___________________________________________________________________

**CONSENT FORM**

I have understood the explanation given to me and I agree to take part in the study.

Signature: Date:----------------------------

Name------------------------------------------------

I certify that I have explained the above to -----------------------------------------------------mother/guardian of -------------------------------------------And that S/he understood what I said and has agreed to take part in the study.

Signature: Date:----------------------------

Name-----------------------------------------------

**Appendix 1b:**

**Therapeutic efficacy of drugs**

**INFORMATION SHEET AND CONSENT FORM**

The Ministry of Health and the Ghana Health service are interested in knowing how well the drugs we are using for IPTc for malaria in the communities in Hohoe district are working. To do this, we are carrying out a study in which we are treating a group of children for malaria and then following them for 28 days to see if their infection is cured. This is not a new treatment formulation as the test drugs are sulphadoxine-pyrimethamine SP (Fansidar) alone or a combination of amodiaquine (AQ) and artesunate (AS).

If you agree to participate in this study, we would like you to bring your child/ward to the clinic 7 or more times over the next 4 weeks, so that we can monitor the progress of the treatment. It is very important that we see your child/ward on these days, so if you feel you will not be able to return on these days, please let us know now. At each visit your child/ward will receive a full medical examination and on 5 of these visits we will take a small amount of blood by finger prick to make blood smears to see if your child still has malaria parasites.

Your participation is a completely voluntary. If you do not want your child/ward to participate in this study, s/he will receive treatment as usual at this clinic. Participation in this study will not cost you or your family anything. You may also withdraw your child/ward from the study at any time and for any reason.

Your child/ward will benefit from participating in this study because s/he will be closely followed over the next 28 days. If your child/ward continues to suffer from malaria, s/he will receive an alternative treatment which will cure the illness. There will be someone here at the clinic every day so that, even on days between scheduled visits and on week ends you may bring your child in for a check-up if you feel that he is ill

Do you have any questions about the study?

If you agree to participate, please sign or thump print the consent form.

___________________________________________________________________

**CONSENT FORM**

I have understood the explanation given to me and I agree to take part in the study.

Signature: Date:----------------------------

Name------------------------------------------------

I certify that I have explained the above to -----------------------------------------------------mother/guardian of -------------------------------------------And that S/he understood what I said and has agreed to take part in the study.

Signature: Date:----------------------------

Name-----------------------------------------------

Appendix 2: Screening & inclusion questionnaire

Sub-District ………………Community………………… date / / / /

**Child**

First Name…………………………….Last Name……………………………………

Date of birth[ ] Sex [ ] ]Weight(Kg) [ ]

**Mother/Guardian’s**

First Name………………………………Last Name…………………………………..

Age (in yrs [ ] Occupation…………………..

House No………Landmark………………………………………………………………….

|  | **Inclusion criteria** | **Yes** | **No** |
| --- | --- | --- | --- |
| 1 | Age between 3 to 59 months |  |  |
| 2 | Resident in the community for at least the next six months |  |  |
| 3 | Has parent or guardian given consent |  |  |
|  | **Exclusion criteria** |  |  |
| **1** | Child unable to take or retain medication |  |  |
| 2 | Existence of respiratory distress |  |  |
| 3 | Existence of serious or chronic illness |  |  |
| 4 | History of drug allergy |  |  |

**Is child eligible? [Yes] [No]**

Anti malarial treatment received (*within the past two weeks*) [Yes] [No]

**Onset of treatment Duration of treatment**

Chloroquine / / / / / / / /

Amodiaquine / / / / / / / /

Fansidar / / / / / / / /

Quinine / / / / / / / /

**Enrolment status**

| Enrolled | Drug code number | Study number |
| --- | --- | --- |
| Not enrolled |  | |

**Appendix 3: Case record form for intermittent treatment**

District………………….Sub-district……………………Community…………… First Name…………………………….Last Name…………………………………

Study Code [ ] Study ID No [ ] Age (in mths) [ ] Sex [ ]

House No………Landmark…………………………………………………………..

**Mother/Guardian’s**

First Name…………………………Last Name………………………Age (in yrs [ ] Occupation…………………..Educational Background………………………………

| Month | **April.** | **May** | **June** | **July** | **Aug.** | **Sept.** | **Oct.** | **Nov.** |
| --- | --- | --- | --- | --- | --- | --- | --- | --- |
| Date of visit |  |  |  |  |  |  |  |  |
| History of Fever or symptoms of malaria [48hrs]Y/N |  |  |  |  |  |  |  |  |
| Axillary temp.( ^o^C ) |  |  |  |  |  |  |  |  |
| Drug administration at home/health facility Y/N |  |  |  |  |  |  |  |  |
| HB(g/dl) |  |  |  |  |  |  |  |  |
| MPs/Per 200WBC |  |  |  |  |  |  |  |  |
| Weight (Kg) |  |  |  |  |  |  |  |  |
| Height/length |  |  |  |  |  |  |  |  |
| MUAC |  |  |  |  |  |  |  |  |
| Vomit (Y/N) |  |  |  |  |  |  |  |  |
| Action taken if vomited |  |  |  |  |  |  |  |  |
| Own bed net(Y/N) |  |  |  |  |  |  |  |  |
| If yes is it treated or been treated within 6 months |  |  |  |  |  |  |  |  |
| Sleep under it last night (Y/N |  |  |  |  |  |  |  |  |

COMMENT:……………………………………………………………………………

**Appendix 4a**

**Drug sensitivity Screening & inclusion form**

Sub-District ………………Community………………… date / / / /

**Child**

First Name…………………………….Last Name……………………………………

Date of birth[ ] Sex [ ] ]Weight(Kg) [ ]

**Mother/Guardian’s**

First Name………………………………Last Name…………………………………..

Age (in yrs [ ] Occupation…………………..

House No………Landmark………………………………………………………………….

|  | **Inclusion criteria** | **Yes** | **No** |
| --- | --- | --- | --- |
| 1 | Age between 3 to 59 months |  |  |
| 2 | Resident in the community for at least the next six months |  |  |
| 3 | Has parent or guardian given consent |  |  |
| 4 | Temperature≥37.5°C |  |  |
| 5 | Malaria parasite count ≥50 per WBCs |  |  |
| 6 | Haemoglobin >6.0g/dl |  |  |
|  | **Exclusion criteria** |  |  |
| 1 | Persistent vomiting (Child unable to take or retain medication) |  |  |
| 2 | Any danger signs |  |  |
| 3 | Existence of respiratory distress |  |  |
| 4 | Existence of serious or chronic illness |  |  |
| 5 | History of drug allergy |  |  |

**Is child eligible? [Yes] [No]**

Anti malarial treatment received (*within the past two weeks*) [Yes] [No]

**Onset of treatment Duration of treatment**

Chloroquine / / / / / / / /

Amodiaquine / / / / / / / /

Fansidar / / / / / / / /

Quinine / / / / / / / /

**Enrolment status**

| Enrolled | Drug code number | Study number |
| --- | --- | --- |
| Not enrolled |  | |

**Appendix 4b: Case record form for therapeutic efficacy study**

District ………………….Facility……………………Community…………………

First Name…………………………….Last Name………………………………………

Study Group [ ] Study No [ ] Age (in mths) [ ] Sex [ ] ]Weight(Kg) [ ]

House No………Landmark………………………………………………..

**Mother/Guardian’s**

First Name………………………………Last Name…………………………………..

Age (in yrs [ ] Occupation…………………..

Educational Background……………………………

|  | **Day 0** | **1** | **2** | **3** | **7** | **14** | **21** | **28** |
| --- | --- | --- | --- | --- | --- | --- | --- | --- |
| Date of visit |  |  |  |  |  |  |  |  |
| Danger signs |  |  |  |  |  |  |  |  |
| History of fever within the last 24hrs Y/N |  |  |  |  |  |  |  |  |
| Axillary temp.( ^o^C ) |  |  |  |  |  |  |  |  |
| Previous medication Y/N |  |  |  |  |  |  |  |  |
| Hb(g/dl) |  |  |  |  |  |  |  |  |
| Malaria parasite count/Per 200WBC |  |  |  |  |  |  |  |  |
| Filter paper |  |  |  |  |  |  |  |  |
| Treatment (No. of tabs) |  |  |  |  |  |  |  |  |
| Vomit Drug (Y/N) |  |  |  |  |  |  |  |  |
| If yes, action taken if |  |  |  |  |  |  |  |  |
| Concomitant treatment |  |  |  |  |  |  |  |  |
| Reasons for exclusion |  |  |  |  |  |  |  |  |

COMMENT:……………………………………………………………………………….………………………………………………………………………………………………………..

| **Overall assessment** | **ETF** | **LTF** | **ACR** | **Excluded** | **Loss to f/up** |
| --- | --- | --- | --- | --- | --- |

**Appendix 5: The Identification card**

IMPACT OF INTERMITTENT PREVENTIVE TREATMENT IN CHILDREN WITH ARTESUNATE PLUS AMODIAQUINE VERSUS SP ALONE ON HAEMOGLOBIN LEVELS AND MALARIA MORBIDITY IN THE HOHOE DISTRICT

District……………………..Sub-District………………Community……………..…

Last name………………………….First name……………………………..

Age (in months) [ ] Sex [ ]

Drug code number_ _______ Study number_________

**THE BEARER OF THIS CARD IS A PARTICIPANT IN THE ABOVE NAMED STUDY.**

**EXAMINE AND MANAGE HIM OR HER AND TREAT WITH QUININE IF YOU SUSPECT MALARIA AND DO NOT ASK HER TO PAY FOR TREATMENT. KINDLY KEEP RECORDS IN THE REGISTER PROVIDED INCLUDING THE DIAGNOSIS AND THE BILL FOR REIMBURSEMENT**

**APPENDIX 6: Register for Microscopy results**

**Name of health facility:**

| **ID** | **name** | **Date** | **Pf asexual** | **Pf gametocytes** | **Other asexual *P* species** | **Other *P.* species gametocytes** | **Date comments** |
| --- | --- | --- | --- | --- | --- | --- | --- |
|  |  |  |  |  |  |  |  |
|  |  |  |  |  |  |  |  |
|  |  |  |  |  |  |  |  |
|  |  |  |  |  |  |  |  |
|  |  |  |  |  |  |  |  |
|  |  |  |  |  |  |  |  |
|  |  |  |  |  |  |  |  |
|  |  |  |  |  |  |  |  |
|  |  |  |  |  |  |  |  |
|  |  |  |  |  |  |  |  |

**APPENDIX 7: Passive surveillance at the health facility**

Name of staff responsible:

| **ID** | **Name** | **Date of reference** | **Date of arrival** | **Temp** | **Diagnosis** | **Treatment given** | **Comments** |
| --- | --- | --- | --- | --- | --- | --- | --- |
|  |  |  |  |  |  |  |  |
|  |  |  |  |  |  |  |  |
|  |  |  |  |  |  |  |  |
|  |  |  |  |  |  |  |  |
|  |  |  |  |  |  |  |  |
|  |  |  |  |  |  |  |  |
|  |  |  |  |  |  |  |  |
|  |  |  |  |  |  |  |  |
|  |  |  |  |  |  |  |  |

**APPENDIX 8: Form for morbidity episodes in the community**

Name Field worker/ coordinator:

| **ID number** | **Name of participant** | **Mother/guardian’s name** | **DOB** | **Fever/**  **vomiting** | **Date of referral** | **Comment** |
| --- | --- | --- | --- | --- | --- | --- |
|  |  |  |  |  |  |  |
|  |  |  |  |  |  |  |
|  |  |  |  |  |  |  |
|  |  |  |  |  |  |  |
|  |  |  |  |  |  |  |
|  |  |  |  |  |  |  |
|  |  |  |  |  |  |  |
|  |  |  |  |  |  |  |
|  |  |  |  |  |  |  |

### APPENDIX 9a: Form for adverse reactions

Village number |___|___| Compound number|___|___|___|

Mother name Mother’s ID Number|___|___|___|___|___|

Child’s name Child’s ID Number: |___|___|___|___|___|

**Notified adverse reactions** **Y** **N**

If yes,

Side effects Date onset Time onset End time Severity[[1]](#footnote-2)1

- |__________| |__________| |___________| |_____|

- |__________| |__________| |___________| |_____|

- |__________| |__________| |___________| |_____|

- |__________| |__________| |___________| |_____|

**APPENDIX 9b: HOHOE IPTc STUDY : ADVERSE EVENTS INVESTIGATION FORM**

|  | 1. AE serial number | | |  |  |  |  |  | |
| --- | --- | --- | --- | --- | --- | --- | --- | --- | --- |
|  | | | | | | | | | |
|  | 2. IDNO |  |  |  |  |  | pin | | |
|  | | | | | | | | |  |
| 3. Initials of the study child (first two letters of the first and last names) | | |  |  |  |  | initi | |  |

| **4.** | The study drug code |  |  |  |  |  |  |  |
| --- | --- | --- | --- | --- | --- | --- | --- | --- |

| **5.** Date on study drug started (day/month/year) ........................ | | | | | | | | | | | | | | | |  | | |  | |  | | |  |  |  | | **DDST** | | | |
| --- | --- | --- | --- | --- | --- | --- | --- | --- | --- | --- | --- | --- | --- | --- | --- | --- | --- | --- | --- | --- | --- | --- | --- | --- | --- | --- | --- | --- | --- | --- | --- |
|  | | | | | | | | | | | | | | | | | | | | | | | | | | | | | | | |
| **6.** Date on study drug stopped (if not stopped, enter 99/99/99) ....... | | | | | | | | | | | | | | | |  | | |  | |  | | |  |  |  | | **DDEN** | | | |
|  | | | | | | | | | | | | | | | | | | | | | | | | | | | | | | | |
| **Details of reaction experienced by participant (please use additional sheet if necessary)** | | | | | | | | | | | | | | | | | | | | | | | | | | | |  | | | |
|  | | | | | | | | | | | | | | | | | | | | | | | | | | | | | | | |
| **7.** Date on which adverse event started (dd/mm/yy) .......................................... | | | | | | | | | | | | | | | |  | | |  | |  | | |  |  |  | | **DAST** | | | |
|  | | | | | | | | | | | | | | | | | | | | | | | | | | | | | | | |
| **8.** Date on reaction stopped (if not stopped, enter 99/99/99) ................... | | | | | | | | | | | | | | | |  | | |  | |  | | |  |  |  | | **DAEN** | | | |
|  | | | | | | | | | | | | | | | | | | | | | | | | | | | | | | | |
| **9.** Was the patient admitted in the hospital .............................. | | | | | | | | | | | | | | | | | | | yes | | 1 | | |  | no | | 2 | **ip** | | | |
|  | | | | | | | | | | | | | | | | | | | | | | | | | | | | | | | |
| **10.** if admitted in hospital, number of days in hospital ....................................... | | | | | | | | | | | | | | | | | | | | | | | | |  |  | | **ipda** | | | |
|  | | | | | | | | | | | | | | | | | | | | | | | | | | | | | | | |
| **11. Describe in detail the nature & timing of the AE/SAE and resolution if known (attach additional pages if necessary):** | | | | | | | | | | | | | | | | | | | | | | | | | | | |  | | | |
| **12. Significant laboratory test results, other clinical conditions, follow-up data (if available). Use separate sheet if necessary:** | | | | | | | | | | | | | | | | | | | | | | | | | | | |  | | | |
|  | | | | | | | | | | | | | | | | | | | | | | | | | | | |  | | | |
| 13. Grade of AE | <grade 3 | | 2 | |  | | Grade 3 | | | | | 3 |  | Grade 4 | | | | | | 4 | |  | SAE | | | 5 | | |  |  | |
|  | | | | | | | | | | | | | | | | | | | | | | | | | | | | | | |  |
| 14. category of SAE | Death | | 1 | |  | | Life threatening | | | | | 2 |  | hospitalisation | | | | | | 3 | |  | disability | | | 4 | | **cat** | | |  |
|  | | | | | | | | | | | | | | | | | | | | | | | | | | | | | | |  |
|  | | Most probably | | | | | | | **1** |  | Probably | | | | **2** | | |  | | possibly | | | | | | **3** | | | **like** | |  |
| **15.** Likelihood that the study  drug caused the AE/SAE | |  | | | | | | |  |  |  | | | | | | | | | | | | | | | | | | | |  |
|  | | Unlikely | | | | | | | **4** |  | Not related | | | | **5** | | |  | | Unclassifiable | | | | | | **6** | | |  | |  |
|  | | | | | | | | | | | | | | | | | | | | | | | | | | | | | | |  |
| **15.** Date on grade 3 or 4 or SAE became known to project physician | | | | | | | | | | | | | | | |  | | |  | |  | | |  |  |  | | **dkno** | | |  |
|  | | | | | | | | | | | | | | | | | | | | | | | | | | | | | | |  |
| **16. Physician’s initials** | |  | |  | |  | | 17. Date reported to DSMB | | | | | | | | |  | |  | |  | | |  |  |  | | **Drep** | | |  |

**Appendix 10– Blister pack of drugs for IPTc courses**

PAQ+AS

AQ+AS

SP

AQ+AS

AQ+AS

AQ+AS

PAQ+AS

PSP

PSP

PAQ+AS

AQ+AS

AQ+AS

Day 1

Day 2

**Placebo arm**

Day 3

**SP arm**

**AQ+AS monthly**

**AQ+AS bimonthly**

**M1, M3 and M5**

**SP=Sulphadoxine-Pyrimethamine**

**AS= artesunate**

**AQ=amodiaquine**

**PSP=Placebo simulating SP**

**PAQ+AS=Placebo simulating AQ +AS**

1. ^1^ Severity: 1=Mild, 2=Moderate, 3=Severe [↑](#footnote-ref-2)
